# Supplementary figures and images for: A survey of HK, HPt, and RR domains and their organization in two-component systems and phosphorelay proteins of organisms with fully sequenced genomes
Source: PeerJ. 2015 Aug 13;3:e1183. doi: 10.7717/peerj.1183 (PMC4558063; doi:10.7717/peerj.1183)

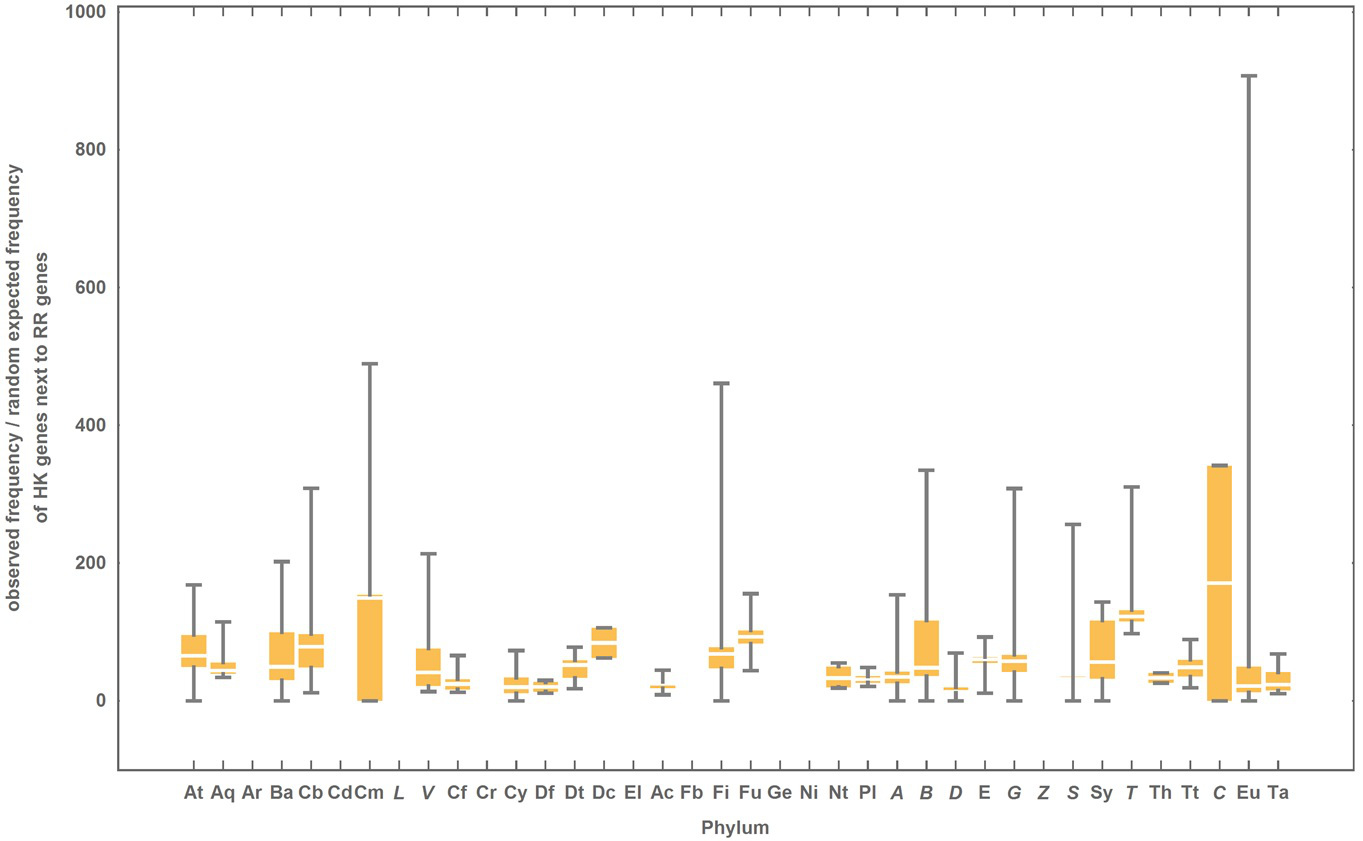

Supplement: Figure S1 — Phylum abbreviations are explained in Table 1. The colored box represents the range of percentage values comprised between the 25% and the 75% quantiles, and the edges of the vertical bar denotes the upper and lower percentage values for each phylum. [file peerj-03-1183-s001.jpg]

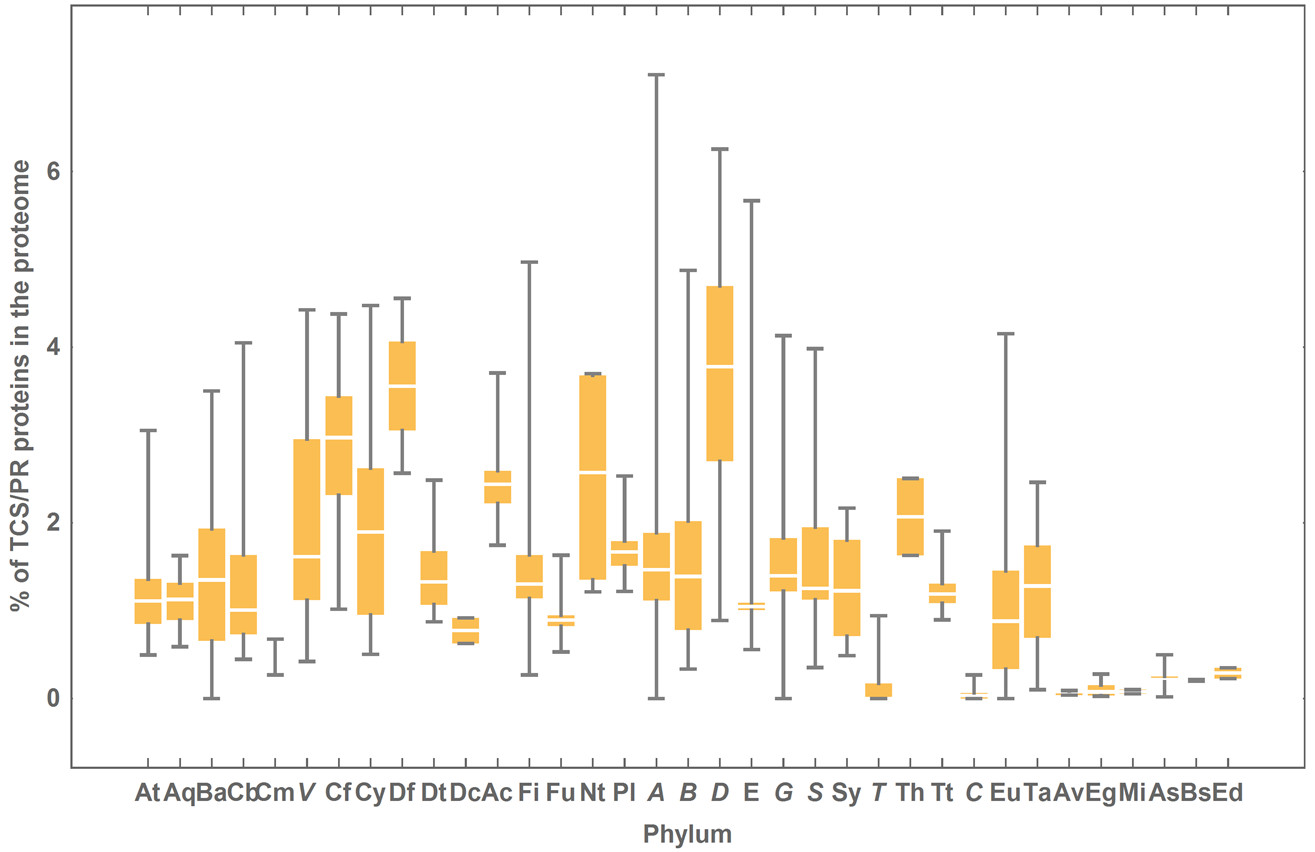

Supplement: Appendix S1 — File including all figures and tables redone to include hypothetical proteins. Results are similar to those obtained for the dataset where these proteins are excluded. [file peerj-03-1183-s011.zip › plus hypothetical and partial/Figure 3.jpg]

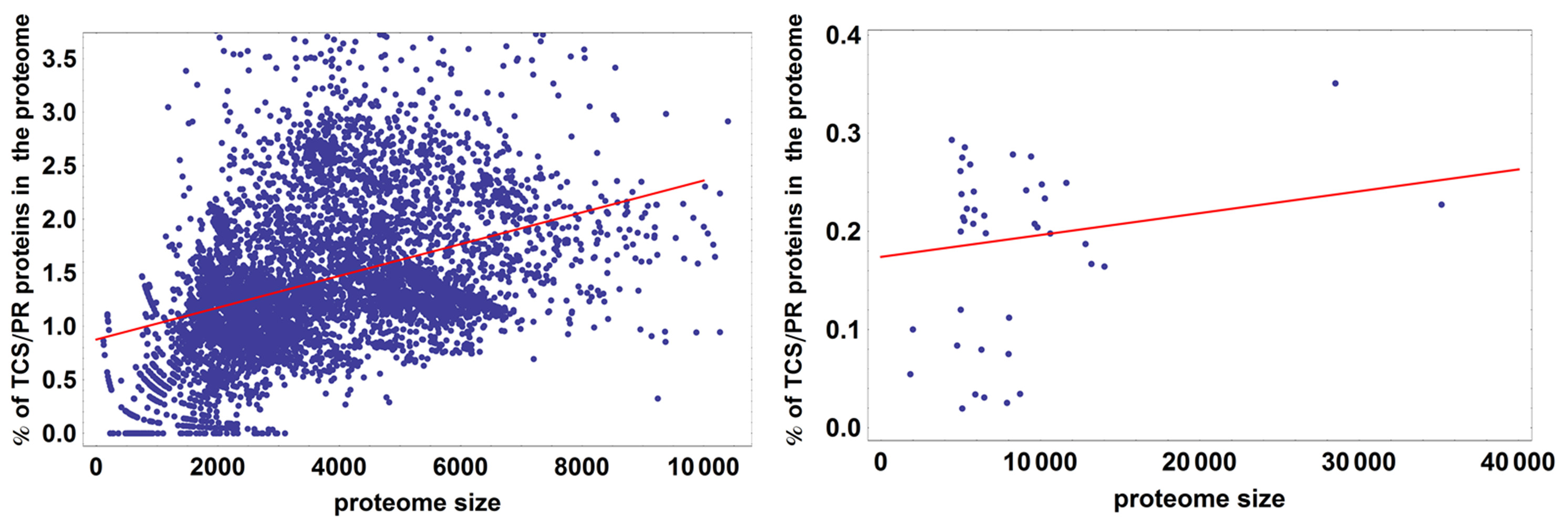

Supplement: Appendix S1 — File including all figures and tables redone to include hypothetical proteins. Results are similar to those obtained for the dataset where these proteins are excluded. [file peerj-03-1183-s011.zip › plus hypothetical and partial/Figure 4.jpg]

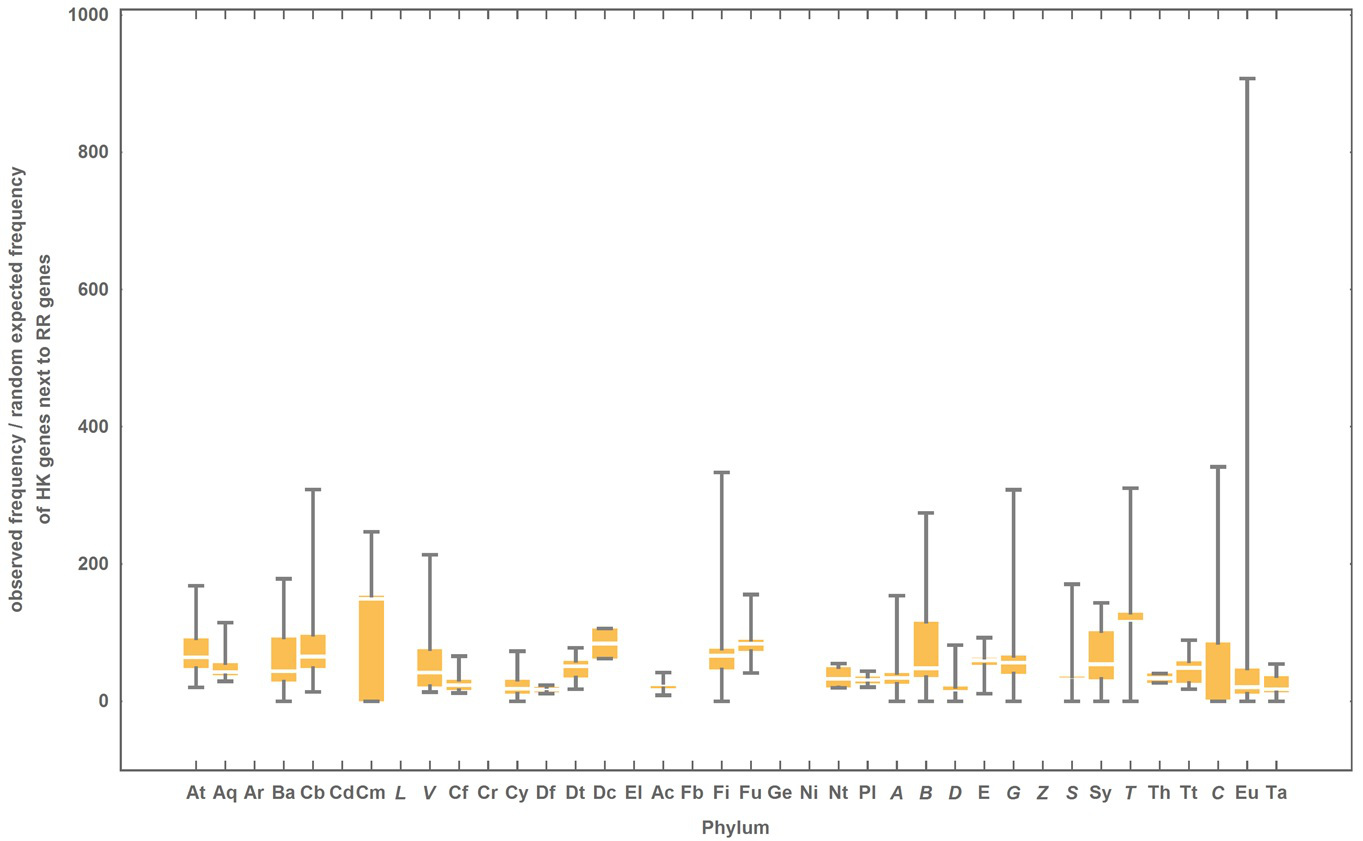

Supplement: Appendix S1 — File including all figures and tables redone to include hypothetical proteins. Results are similar to those obtained for the dataset where these proteins are excluded. [file peerj-03-1183-s011.zip › plus hypothetical and partial/Supplementary Figure 1.jpg]
