# Supplementary material for: A survey of HK, HPt, and RR domains and their organization in two-component systems and phosphorelay proteins of organisms with fully sequenced genomes
Source: PeerJ. 2015 Aug 13;3:e1183. doi: 10.7717/peerj.1183 (PMC4558063; doi:10.7717/peerj.1183)
Supplement: Table S1 [file peerj-03-1183-s002.docx]

| Actinobacteria | Acidimicrobidae bacterium YM16 304,Acidimicrobium ferrooxidans DSM 10331,Acidothermus cellulolyticus 11B,Actinoplanes friuliensis DSM 7358,Actinoplanes missouriensis 431,Actinoplanes N902 109,Actinoplanes SE50 110,Actinosynnema mirum DSM 43827,Amycolatopsis mediterranei RB,Amycolatopsis mediterranei S699,Amycolatopsis mediterranei U32,Amycolatopsis orientalis HCCB10007,Amycolicicoccus subflavus DQS3 9A1,Arcanobacterium haemolyticum DSM 20595,Arthrobacter arilaitensis Re117,Arthrobacter aurescens TC1,Arthrobacter chlorophenolicus A6,Arthrobacter FB24,Arthrobacter nitroguajacolicus Rue61a,Arthrobacter phenanthrenivorans Sphe3,Atopobium parvulum DSM 20469,Beutenbergia cavernae DSM 12333,Bifidobacterium 12 1 47BFAA,Bifidobacterium adolescentis ATCC 15703,Bifidobacterium adolescentis L2 32,Bifidobacterium angulatum DSM 20098,Bifidobacterium animalis ATCC 25527,Bifidobacterium animalis lactis AD011,Bifidobacterium animalis lactis ATCC 27673,Bifidobacterium animalis lactis B420,Bifidobacterium animalis lactis BB 12,Bifidobacterium animalis lactis Bi 07,Bifidobacterium animalis lactis Bl12,Bifidobacterium animalis lactis Bl 04,Bifidobacterium animalis lactis BLC1,Bifidobacterium animalis lactis BS 01,Bifidobacterium animalis lactis CNCM I 2494,Bifidobacterium animalis lactis DSM 10140,Bifidobacterium animalis lactis HN019,Bifidobacterium animalis lactis V9,Bifidobacterium asteroides PRL2011,Bifidobacterium bifidum BGN4,Bifidobacterium bifidum IPLA 20015,Bifidobacterium bifidum LMG 13195,Bifidobacterium bifidum NCIMB 41171,Bifidobacterium bifidum PRL2010,Bifidobacterium bifidum S17,Bifidobacterium breve ACS 071 V Sch8b,Bifidobacterium breve CECT 7263,Bifidobacterium breve DSM 20213,Bifidobacterium breve UCC2003,Bifidobacterium catenulatum DSM 16992,Bifidobacterium dentium ATCC 27678,Bifidobacterium dentium ATCC 27679,Bifidobacterium dentium Bd1,Bifidobacterium dentium JCVIHMP022,Bifidobacterium gallicum DSM 20093,Bifidobacterium longum 1 6B,Bifidobacterium longum 2 2B,Bifidobacterium longum 35B,Bifidobacterium longum 44B,Bifidobacterium longum BBMN68,Bifidobacterium longum DJO10A,Bifidobacterium longum F8,Bifidobacterium longum infantis 157F,Bifidobacterium longum infantis ATCC 15697,Bifidobacterium longum infantis ATCC 55813,Bifidobacterium longum infantis CCUG 52486,Bifidobacterium longum JCM 1217,Bifidobacterium longum JDM301,Bifidobacterium longum KACC 91563,Bifidobacterium longum NCC2705,Bifidobacterium pseudocatenulatum DSM 20438,Bifidobacterium thermophilum RBL67,Blastococcus saxobsidens DD2,Brachybacterium faecium DSM 4810,Brachybacterium paraconglomeratum LC44,Brachybacterium squillarum M 6 3,Catenulispora acidiphila DSM 44928,Cellulomonas fimi ATCC 484,Cellulomonas flavigena DSM 20109,Cellvibrio gilvus ATCC 13127,Clavibacter michiganensis NCPPB 382,Clavibacter michiganensis nebraskensis NCPPB 2581,Clavibacter michiganensis sepedonicus,Conexibacter woesei DSM 14684,Coriobacterium glomerans PW2,Corynebacterium accolens ATCC 49725,Corynebacterium accolens ATCC 49726,Corynebacterium ammoniagenes DSM 20306,Corynebacterium amycolatum SK46,Corynebacterium argentoratense DSM 44202,Corynebacterium aurimucosum ATCC 700975,Corynebacterium bovis DSM 20582,Corynebacterium callunae DSM 20147,Corynebacterium casei,Corynebacterium diphtheriae 241,Corynebacterium diphtheriae 31A,Corynebacterium diphtheriae BH8,Corynebacterium diphtheriae bv intermedius NCTC 5011,Corynebacterium diphtheriae C7 beta,Corynebacterium diphtheriae CDCE 8392,Corynebacterium diphtheriae HC01,Corynebacterium diphtheriae HC02,Corynebacterium diphtheriae HC03,Corynebacterium diphtheriae HC04,Corynebacterium diphtheriae INCA 402,Corynebacterium diphtheriae NCTC 13129,Corynebacterium diphtheriae PW8,Corynebacterium diphtheriae VA01,Corynebacterium durum F0235,Corynebacterium efficiens YS 314,Corynebacterium genitalium ATCC 33030,Corynebacterium glucuronolyticum ATCC 51866,Corynebacterium glucuronolyticum ATCC 51867,Corynebacterium glutamicum ATCC 13032,Corynebacterium glutamicum ATCC 14067,Corynebacterium glutamicum MB001,Corynebacterium glutamicum R,Corynebacterium glutamicum S9114,Corynebacterium glutamicum SCgG1,Corynebacterium glutamicum SCgG2,Corynebacterium halotolerans YIM 70093 DSM 44683,Corynebacterium jeikeium ATCC 43734,Corynebacterium jeikeium K411,Corynebacterium kroppenstedtii DSM 44385,Corynebacterium lipophiloflavum DSM 44291,Corynebacterium maris DSM 45190,Corynebacterium matruchotii ATCC 14266,Corynebacterium matruchotii ATCC 33806,Corynebacterium nuruki S6 4,Corynebacterium pseudogenitalium ATCC 33035,Corynebacterium pseudotuberculosis 1002,Corynebacterium pseudotuberculosis 1 06 A,Corynebacterium pseudotuberculosis 258,Corynebacterium pseudotuberculosis 267,Corynebacterium pseudotuberculosis 31,Corynebacterium pseudotuberculosis 316,Corynebacterium pseudotuberculosis 3 99 5,Corynebacterium pseudotuberculosis 42 02 A,Corynebacterium pseudotuberculosis C231,Corynebacterium pseudotuberculosis CIP 52 97,Corynebacterium pseudotuberculosis Cp162,Corynebacterium pseudotuberculosis FRC41,Corynebacterium pseudotuberculosis I19,Corynebacterium pseudotuberculosis P54B96,Corynebacterium pseudotuberculosis PAT10,Corynebacterium resistens DSM 45100,Corynebacterium striatum ATCC 6940,Corynebacterium terpenotabidum Y 11,Corynebacterium tuberculostearicum SK141,Corynebacterium ulcerans 0102,Corynebacterium ulcerans 809,Corynebacterium ulcerans BR AD22,Corynebacterium urealyticum DSM 7109,Corynebacterium urealyticum DSM 7111,Corynebacterium variabile DSM 44702,Cryptobacterium curtum DSM 15641,Eggerthella 1 3 56FAA,Eggerthella HGA1,Eggerthella lenta DSM 2243,Eggerthella YY7918,Frankia alni ACN14a,Frankia CcI3,Frankia CN3,Frankia EAN1pec,Frankia EuI1c,Frankia EUN1f,Frankia QA3,Frankia symbiont of Datisca glomerata,Gardnerella vaginalis 00703Bmash,Gardnerella vaginalis 00703C2mash,Gardnerella vaginalis 00703Dmash,Gardnerella vaginalis 0288E,Gardnerella vaginalis 1400E,Gardnerella vaginalis 1500E,Gardnerella vaginalis 284V,Gardnerella vaginalis 315 A,Gardnerella vaginalis 409 05,Gardnerella vaginalis 55152,Gardnerella vaginalis 5 1,Gardnerella vaginalis 6119V5,Gardnerella vaginalis 6420B,Gardnerella vaginalis 6420LIT,Gardnerella vaginalis 75712,Gardnerella vaginalis AMD,Gardnerella vaginalis ATCC 14018,Gardnerella vaginalis ATCC 14019,Gardnerella vaginalis HMP9231,Geodermatophilus obscurus DSM 43160,Gordonia aichiensis NBRC 108223,Gordonia alkanivorans NBRC 16433,Gordonia amarae NBRC 15530,Gordonia amicalis NBRC 100051,Gordonia araii NBRC 100433,Gordonia bronchialis DSM 43247,Gordonia effusa NBRC 100432,Gordonia hirsuta DSM 44140 NBRC 16056,Gordonia KTR9,Gordonia malaquae NBRC 108250,Gordonia namibiensis NBRC 108229,Gordonia neofelifaecis NRRL B 59395,Gordonia otitidis NBRC 100426,Gordonia paraffinivorans NBRC 108238,Gordonia polyisoprenivorans NBRC 16320,Gordonia polyisoprenivorans VH2,Gordonia rhizosphera NBRC 16068,Gordonia rubripertincta NBRC 101908,Gordonia sihwensis NBRC 108236,Gordonia soli NBRC 108243,Gordonia sputi NBRC 100414,Gordonia terrae NBRC 100016,Gordonibacter pamelaeae 7 10 1 b,Intrasporangium calvum DSM 43043,Isoptericola variabilis 225,Jonesia denitrificans DSM 20603,Kineococcus radiotolerans SRS30216,Kitasatospora setae KM 6054,Kocuria palustris TAGA27,Kocuria rhizophila DC2201,Kribbella flavida DSM 17836,Kytococcus sedentarius DSM 20547,Leifsonia xyli CTCB07,Leifsonia xyli cynodontis DSM 46306,Microbacterium laevaniformans OR221,Microbacterium testaceum StLB037,Micrococcus luteus NCTC 2665,Micrococcus luteus SK58,Microlunatus phosphovorus NM 1,Micromonospora ATCC 39149,Micromonospora aurantiaca ATCC 27029,Micromonospora L5,Micromonospora lupini Lupac 08,Mobiluncus curtisii ATCC 35241,Mobiluncus curtisii ATCC 43063,Mobiluncus curtisii ATCC 51333,Mobiluncus curtisii holmesii ATCC 35242,Mobiluncus mulieris 28 1,Mobiluncus mulieris ATCC 35239,Mobiluncus mulieris ATCC 35243,Mobiluncus mulieris FB024 16,Modestobacter marinus,Mycobacterium abscessus,Mycobacterium abscessus 3A 0119 R,Mycobacterium abscessus 3A 0122 R,Mycobacterium abscessus 3A 0122 S,Mycobacterium abscessus 3A 0731,Mycobacterium abscessus 3A 0810 R,Mycobacterium abscessus 3A 0930 R,Mycobacterium abscessus 3A 0930 S,Mycobacterium abscessus 47J26,Mycobacterium abscessus 4S 0116 R,Mycobacterium abscessus 4S 0116 S,Mycobacterium abscessus 4S 0206,Mycobacterium abscessus 4S 0303,Mycobacterium abscessus 4S 0726 RA,Mycobacterium abscessus 4S 0726 RB,Mycobacterium abscessus 5S 0304,Mycobacterium abscessus 5S 0421,Mycobacterium abscessus 5S 0422,Mycobacterium abscessus 5S 0708,Mycobacterium abscessus 5S 0817,Mycobacterium abscessus 5S 0921,Mycobacterium abscessus 5S 1212,Mycobacterium abscessus 5S 1215,Mycobacterium abscessus 6G 0125 R,Mycobacterium abscessus 6G 0125 S,Mycobacterium abscessus 6G 0212,Mycobacterium abscessus 6G 0728 R,Mycobacterium abscessus 6G 0728 S,Mycobacterium abscessus 6G 1108,Mycobacterium abscessus bolletii 50594,Mycobacterium abscessus bolletii BD,Mycobacterium abscessus M93,Mycobacterium abscessus M94,Mycobacterium africanum GM041182,Mycobacterium avium 104,Mycobacterium avium ATCC 25291,Mycobacterium avium paratuberculosis K 10,Mycobacterium avium paratuberculosis MAP4,Mycobacterium avium paratuberculosis S397,Mycobacterium avium paratuberculosis S5,Mycobacterium bovis AF2122 97,Mycobacterium bovis BCG Korea 1168P,Mycobacterium bovis BCG Mexico,Mycobacterium bovis BCG Pasteur 1173P2,Mycobacterium bovis BCG Tokyo 172,Mycobacterium canettii CIPT 140010059,Mycobacterium canettii CIPT 140060008,Mycobacterium canettii CIPT 140070008,Mycobacterium canettii CIPT 140070010,Mycobacterium canettii CIPT 140070017,Mycobacterium chubuense NBB4,Mycobacterium colombiense CECT 3035,Mycobacterium fortuitum DSM 46621,Mycobacterium gilvum PYR GCK,Mycobacterium gilvum Spyr1,Mycobacterium H4Y,Mycobacterium hassiacum DSM 44199,Mycobacterium indicus pranii MTCC 9506,Mycobacterium intracellulare ATCC 13950,Mycobacterium intracellulare MOTT 02,Mycobacterium intracellulare MOTT 64,Mycobacterium JDM601,Mycobacterium JLS,Mycobacterium kansasii ATCC 12478,Mycobacterium KMS,Mycobacterium leprae Br4923,Mycobacterium leprae TN,Mycobacterium liflandii 128FXT,Mycobacterium marinum M,Mycobacterium massiliense 1S 151 0930,Mycobacterium massiliense 1S 152 0914,Mycobacterium massiliense 1S 153 0915,Mycobacterium massiliense 1S 154 0310,Mycobacterium massiliense 2B 0107,Mycobacterium massiliense 2B 0307,Mycobacterium massiliense 2B 0626,Mycobacterium massiliense 2B 0912 R,Mycobacterium massiliense 2B 0912 S,Mycobacterium massiliense 2B 1231,Mycobacterium massiliense CCUG 48898,Mycobacterium massiliense GO 06,Mycobacterium MCS,Mycobacterium MOTT36Y,Mycobacterium parascrofulaceum ATCC BAA 614,Mycobacterium phlei RIVM601174,Mycobacterium rhodesiae JS60,Mycobacterium rhodesiae NBB3,Mycobacterium smegmatis JS623,Mycobacterium smegmatis MC2 155,Mycobacterium smegmatis MKD8,Mycobacterium thermoresistibile ATCC 19527,Mycobacterium tuberculosis,Mycobacterium tuberculosis 02 1987,Mycobacterium tuberculosis 210,Mycobacterium tuberculosis 94 M4241A,Mycobacterium tuberculosis 98 R604 INH RIF EM,Mycobacterium tuberculosis Beijing NITR203,Mycobacterium tuberculosis C,Mycobacterium tuberculosis CAS NITR204,Mycobacterium tuberculosis CCDC5079,Mycobacterium tuberculosis CCDC5180,Mycobacterium tuberculosis CDC1551,Mycobacterium tuberculosis CDC1551A,Mycobacterium tuberculosis CPHL A,Mycobacterium tuberculosis CTRI 2,Mycobacterium tuberculosis EAI5,Mycobacterium tuberculosis EAI5 NITR206,Mycobacterium tuberculosis EAS054,Mycobacterium tuberculosis Erdman ATCC 35801,Mycobacterium tuberculosis F11,Mycobacterium tuberculosis GM 1503,Mycobacterium tuberculosis H37Ra,Mycobacterium tuberculosis H37Rv,Mycobacterium tuberculosis Haarlem,Mycobacterium tuberculosis Haarlem3 NITR202,Mycobacterium tuberculosis K85,Mycobacterium tuberculosis KZN 1435,Mycobacterium tuberculosis KZN 4207,Mycobacterium tuberculosis KZN 605,Mycobacterium tuberculosis KZN R506,Mycobacterium tuberculosis KZN V2475,Mycobacterium tuberculosis NCGM2209,Mycobacterium tuberculosis RGTB327,Mycobacterium tuberculosis RGTB423,Mycobacterium tuberculosis SUMu001,Mycobacterium tuberculosis SUMu002,Mycobacterium tuberculosis SUMu003,Mycobacterium tuberculosis SUMu004,Mycobacterium tuberculosis SUMu005,Mycobacterium tuberculosis SUMu006,Mycobacterium tuberculosis SUMu007,Mycobacterium tuberculosis SUMu008,Mycobacterium tuberculosis SUMu009,Mycobacterium tuberculosis SUMu010,Mycobacterium tuberculosis SUMu011,Mycobacterium tuberculosis SUMu012,Mycobacterium tuberculosis T17,Mycobacterium tuberculosis T46,Mycobacterium tuberculosis T85,Mycobacterium tuberculosis T92,Mycobacterium tuberculosis UT205,Mycobacterium tuberculosis W 148,Mycobacterium tusciae JS617,Mycobacterium ulcerans Agy99,Mycobacterium vaccae ATCC 25954,Mycobacterium vanbaalenii PYR 1,Mycobacterium VKM Ac 1815D,Mycobacterium xenopi RIVM700367,Mycobacterium yongonense 05 1390,Nakamurella multipartita DSM 44233,Nocardia brasiliensis ATCC 700358,Nocardia cyriacigeorgica GUH 2,Nocardia farcinica IFM 10152,Nocardioides JS614,Nocardiopsis alba ATCC BAA 2165,Nocardiopsis dassonvillei DSM 43111,Olsenella oral taxon 809 F0356,Olsenella uli DSM 7084,Propionibacterium 409 HC1,Propionibacterium 434 HC2,Propionibacterium 5 U 42AFAA,Propionibacterium acidipropionici ATCC 4875,Propionibacterium acnes 266,Propionibacterium acnes 6609,Propionibacterium acnes ATCC 11828,Propionibacterium acnes C1,Propionibacterium acnes FZ1 2 0,Propionibacterium acnes HL001PA1,Propionibacterium acnes HL002PA1,Propionibacterium acnes HL002PA2,Propionibacterium acnes HL002PA3,Propionibacterium acnes HL005PA1,Propionibacterium acnes HL005PA2,Propionibacterium acnes HL005PA3,Propionibacterium acnes HL005PA4,Propionibacterium acnes HL007PA1,Propionibacterium acnes HL013PA1,Propionibacterium acnes HL013PA2,Propionibacterium acnes HL020PA1,Propionibacterium acnes HL025PA1,Propionibacterium acnes HL025PA2,Propionibacterium acnes HL027PA1,Propionibacterium acnes HL027PA2,Propionibacterium acnes HL030PA1,Propionibacterium acnes HL030PA2,Propionibacterium acnes HL036PA1,Propionibacterium acnes HL036PA2,Propionibacterium acnes HL036PA3,Propionibacterium acnes HL037PA1,Propionibacterium acnes HL037PA2,Propionibacterium acnes HL037PA3,Propionibacterium acnes HL038PA1,Propionibacterium acnes HL043PA1,Propionibacterium acnes HL043PA2,Propionibacterium acnes HL044PA1,Propionibacterium acnes HL045PA1,Propionibacterium acnes HL046PA1,Propionibacterium acnes HL046PA2,Propionibacterium acnes HL050PA1,Propionibacterium acnes HL050PA2,Propionibacterium acnes HL050PA3,Propionibacterium acnes HL053PA1,Propionibacterium acnes HL053PA2,Propionibacterium acnes HL056PA1,Propionibacterium acnes HL059PA1,Propionibacterium acnes HL059PA2,Propionibacterium acnes HL060PA1,Propionibacterium acnes HL063PA1,Propionibacterium acnes HL063PA2,Propionibacterium acnes HL067PA1,Propionibacterium acnes HL072PA1,Propionibacterium acnes HL072PA2,Propionibacterium acnes HL074PA1,Propionibacterium acnes HL078PA1,Propionibacterium acnes HL082PA1,Propionibacterium acnes HL082PA2,Propionibacterium acnes HL083PA1,Propionibacterium acnes HL083PA2,Propionibacterium acnes HL086PA1,Propionibacterium acnes HL087PA1,Propionibacterium acnes HL087PA2,Propionibacterium acnes HL087PA3,Propionibacterium acnes HL092PA1,Propionibacterium acnes HL096PA1,Propionibacterium acnes HL096PA2,Propionibacterium acnes HL096PA3,Propionibacterium acnes HL097PA1,Propionibacterium acnes HL099PA1,Propionibacterium acnes HL103PA1,Propionibacterium acnes HL110PA1,Propionibacterium acnes HL110PA2,Propionibacterium acnes HL110PA3,Propionibacterium acnes HL110PA4,Propionibacterium acnes J139,Propionibacterium acnes J165,Propionibacterium acnes KPA171202,Propionibacterium acnes PRP 38,Propionibacterium acnes SK137,Propionibacterium acnes SK182,Propionibacterium acnes SK182B JCVI,Propionibacterium acnes SK187,Propionibacterium acnes TypeIA2 P acn17,Propionibacterium acnes TypeIA2 P acn31,Propionibacterium acnes TypeIA2 P acn33,Propionibacterium avidum 44067,Propionibacterium avidum ATCC 25577,Propionibacterium CC003 HC2,Propionibacterium freudenreichii shermanii CIRM BIA1,Propionibacterium humerusii P08,Propionibacterium propionicum F0230a,Pseudonocardia dioxanivorans CB1190,Pseudonocardia P1,Renibacterium salmoninarum ATCC 33209,Rhodococcus AW25M09,Rhodococcus equi 103S,Rhodococcus equi ATCC 33707,Rhodococcus erythropolis CCM2595,Rhodococcus erythropolis PR4,Rhodococcus erythropolis SK121,Rhodococcus imtechensis RKJ300,Rhodococcus jostii RHA1,Rhodococcus JVH1,Rhodococcus opacus B4,Rhodococcus opacus M213,Rhodococcus opacus PD630,Rhodococcus P14,Rhodococcus pyridinivorans AK37,Rhodococcus pyridinivorans SB3094,Rhodococcus qingshengii BKS 20 40,Rhodococcus ruber BKS 20 38,Rhodococcus triatomae BKS 15 14,Rhodococcus wratislaviensis IFP 2016,Rothia aeria F0474,Rothia dentocariosa ATCC 17931,Rothia dentocariosa M567,Rothia mucilaginosa,Rothia mucilaginosa ATCC 25296,Rothia mucilaginosa M508,Rubrobacter xylanophilus DSM 9941,Saccharomonospora azurea SZMC 14600,Saccharomonospora cyanea NA 134,Saccharomonospora glauca K62,Saccharomonospora paurometabolica YIM 90007,Saccharomonospora viridis DSM 43017,Saccharomonospora xinjiangensis XJ 54,Saccharopolyspora erythraea NRRL 2338,Saccharopolyspora spinosa NRRL 18395,Saccharothrix espanaensis DSM 44229,Salinispora arenicola CNS 205,Salinispora tropica CNB 440,Sanguibacter keddieii DSM 10542,Segniliparus rotundus DSM 44985,Segniliparus rugosus ATCC BAA 974,Slackia CM382,Slackia exigua ATCC 700122,Slackia heliotrinireducens DSM 20476,Slackia piriformis YIT 12062,Stackebrandtia nassauensis DSM 44728,Streptomyces AA4,Streptomyces acidiscabies 84 104,Streptomyces ACT 1,Streptomyces albus J1074,Streptomyces auratus AGR0001,Streptomyces avermitilis MA 4680,Streptomyces bingchenggensis BCW 1,Streptomyces bottropensis ATCC 25435,Streptomyces C,Streptomyces cattleya NRRL 8057,Streptomyces cattleya NRRL 8057 DSM 46488,Streptomyces chartreusis NRRL 12338,Streptomyces clavuligerus ATCC 27064,Streptomyces coelicoflavus ZG0656,Streptomyces coelicolor A3 2,Streptomyces collinus Tu 365,Streptomyces davawensis JCM 4913,Streptomyces e14,Streptomyces flavogriseus ATCC 33331,Streptomyces fulvissimus DSM 40593,Streptomyces gancidicus BKS 13 15,Streptomyces ghanaensis ATCC 14672,Streptomyces globisporus C 1027,Streptomyces griseoaurantiacus M045,Streptomyces griseoflavus Tu4000,Streptomyces griseus NBRC 13350,Streptomyces hygroscopicus ATCC 53653,Streptomyces hygroscopicus jinggangensis 5008,Streptomyces hygroscopicus jinggangensis TL01,Streptomyces lividans TK24,Streptomyces Mg1,Streptomyces mobaraensis NBRC 13819,Streptomyces PAMC26508,Streptomyces pristinaespiralis ATCC 25486,Streptomyces rapamycinicus NRRL 5491,Streptomyces rimosus ATCC 10970,Streptomyces roseosporus NRRL 11379,Streptomyces roseosporus NRRL 15998,Streptomyces S4,Streptomyces SA3 actF,Streptomyces SA3 actG,Streptomyces scabiei 87 22,Streptomyces SirexAA E,Streptomyces SM8,Streptomyces somaliensis DSM 40738,Streptomyces SPB74,Streptomyces SPB78,Streptomyces sviceus ATCC 29083,Streptomyces tsukubaensis NRRL18488,Streptomyces Tu6071,Streptomyces turgidiscabies Car8,Streptomyces venezuelae ATCC 10712,Streptomyces violaceusniger Tu 4113,Streptomyces viridochromogenes DSM 40736,Streptomyces viridochromogenes Tue57,Streptomyces W007,Streptomyces zinciresistens K42,Streptosporangium roseum DSM 43021,Thermobifida fusca YX,Thermobispora bispora DSM 43833,Thermomonospora curvata DSM 43183,Tropheryma whipplei TW08 27,Tropheryma whipplei Twist,Tsukamurella paurometabola DSM 20162,Verrucosispora maris AB 18 032,Xylanimonas cellulosilytica DSM 15894,Adlercreutzia equolifaciens DSM 19450,Brevibacterium linens BL2,Brevibacterium mcbrellneri ATCC 49030,Citricoccus CH26A,Collinsella aerofaciens ATCC 25986,Collinsella intestinalis DSM 13280,Collinsella stercoris DSM 13279,Collinsella tanakaei YIT 12063,Dermacoccus Ellin185,Dietzia alimentaria 72,Dietzia cinnamea P4,Janibacter hoylei PVAS 1,Janibacter HTCC2649,Kineosphaera limosa NBRC 100340,Leucobacter chromiiresistens JG 31,Mobilicoccus pelagius NBRC 104925,Nesterenkonia F,Nocardioidaceae bacterium Broad 1,Parascardovia denticolens DSM 10105,Parascardovia denticolens F0305,Parascardovia denticolens IPLA 20019,Patulibacter I11,Salinibacterium PAMC 21357,Scardovia inopinata F0304,Scardovia wiggsiae F0424,Serinicoccus profundi MCCC 1A05965,Turicella otitidis ATCC 51513,Candidatus Aquiluna IMCC13023,marine actinobacterium PHSC20C1 |
| --- | --- |
| Aquificae | Hydrogenivirga 128 5 R1 1, Aquifex aeolicus VF5,Desulfurobacterium thermolithotrophum DSM 11699,Hydrogenobacter thermophilus TK 6,Hydrogenobaculum HO,Hydrogenobaculum SN,Hydrogenobaculum Y04AAS1,Persephonella marina EX H1,Sulfurihydrogenibium azorense Az Fu1,Sulfurihydrogenibium yellowstonense SS 5,Sulfurihydrogenibium YO3AOP1,Thermocrinis albus DSM 14484,Thermovibrio ammonificans HB 1 |
| Armatimonadetes | Chthonomonas calidirosea T49 |
| Bacteroidetes | Aequorivita sublithincola DSM 14238,Alistipes finegoldii DSM 17242,Alistipes shahii WAL 8301,Bacteroides CF50,Bacteroides fragilis 638R,Bacteroides fragilis NCTC 9343,Bacteroides fragilis YCH46,Bacteroides helcogenes P 36 108,Bacteroides salanitronis DSM 18170,Bacteroides thetaiotaomicron VPI 5482,Bacteroides vulgatus ATCC 8482,Bacteroides vulgatus EK4 2,Bacteroides vulgatus PC510,Bacteroides xylanisolvens CL03T12C04,Bacteroides xylanisolvens SD CC 1b,Bacteroides xylanisolvens XB1A,Belliella baltica DSM 15883,Blattabacterium Blaberus giganteus,Blattabacterium Blatta orientalis Tarazona,Blattabacterium Blattella germanica Bge,Blattabacterium Cryptocercus punctulatus Cpu,Blattabacterium Mastotermes darwiniensis MADAR,Blattabacterium Nauphoeta cinerea,Blattabacterium Panesthia angustipennis spadica BPAA,Blattabacterium Periplaneta americana BPLAN,Candidatus Amoebophilus asiaticus 5a2,Candidatus Azobacteroides pseudotrichonymphae genomovar CFP2,Candidatus Sulcia muelleri CARI,Candidatus Sulcia muelleri DMIN,Candidatus Sulcia muelleri GWSS,Candidatus Sulcia muelleri Hc Homalodisca coagulata,Candidatus Sulcia muelleri SMDSEM,Candidatus Sulcia muelleri Sulcia ALF,Candidatus Uzinura diaspidicola ASNER,Capnocytophaga canimorsus Cc5,Capnocytophaga CM59,Capnocytophaga gingivalis ATCC 33624,Capnocytophaga ochracea DSM 7271,Capnocytophaga ochracea F0287,Capnocytophaga ochracea Holt 25,Capnocytophaga oral taxon 324 F0483,Capnocytophaga oral taxon 326 F0382,Capnocytophaga oral taxon 329 F0087,Capnocytophaga oral taxon 332 F0381,Capnocytophaga oral taxon 335 F0486,Capnocytophaga oral taxon 338 F0234,Capnocytophaga oral taxon 380 F0488,Capnocytophaga oral taxon 412 F0487,Capnocytophaga sputigena ATCC 33612,Cardinium endosymbiont cEper1 of Encarsia pergandiella,Cellulophaga algicola DSM 14237,Cellulophaga lytica DSM 7489,Chitinophaga pinensis DSM 2588,Croceibacter atlanticus HTCC2559,Cyclobacterium marinum DSM 745,Cytophaga hutchinsonii ATCC 33406,Dyadobacter fermentans DSM 18053,Echinicola vietnamensis DSM 17526,Emticicia oligotrophica DSM 17448,Fibrella aestuarina BUZ 2,Flavobacterium branchiophilum FL 15,Flavobacterium CF136,Flavobacterium columnare ATCC 49512,Flavobacterium F52,Flavobacterium frigoris PS1,Flavobacterium indicum GPTSA100 9,Flavobacterium johnsoniae UW101,Flavobacterium psychrophilum JIP02 86,Flexibacter litoralis DSM 6794,Fluviicola taffensis DSM 16823,Gramella forsetii KT0803,Haliscomenobacter hydrossis DSM 1100,Krokinobacter 4H 3 7 5,Lacinutrix 5H 3 7 4,Leadbetterella byssophila DSM 17132,Marivirga tractuosa DSM 4126,Muricauda ruestringensis DSM 13258,Niastella koreensis GR20 10,Odoribacter laneus YIT 12061,Odoribacter splanchnicus DSM 20712,Ornithobacterium rhinotracheale DSM 15997,Owenweeksia hongkongensis DSM 17368,Paludibacter propionicigenes WB4,Parabacteroides D13,Parabacteroides D25,Parabacteroides distasonis ATCC 8503,Parabacteroides distasonis CL03T12C09,Parabacteroides distasonis CL09T03C24,Parabacteroides goldsteinii CL02T12C30,Parabacteroides johnsonii CL02T12C29,Parabacteroides johnsonii DSM 18315,Parabacteroides merdae ATCC 43184,Parabacteroides merdae CL03T12C32,Parabacteroides merdae CL09T00C40,Pedobacter agri PB92,Pedobacter BAL39,Pedobacter heparinus DSM 2366,Pedobacter saltans DSM 12145,Polaribacter irgensii 23 P,Polaribacter MED152,Porphyromonas asaccharolytica DSM 20707,Porphyromonas asaccharolytica PR426713P I,Porphyromonas catoniae F0037,Porphyromonas endodontalis ATCC 35406,Porphyromonas gingivalis ATCC 33277,Porphyromonas gingivalis TDC60,Porphyromonas gingivalis W50,Porphyromonas gingivalis W83,Porphyromonas oral taxon 279 F0450,Porphyromonas uenonis 60 3,Prevotella amnii CRIS 21A A,Prevotella bergensis DSM 17361,Prevotella bivia DSM 20514,Prevotella bivia JCVIHMP010,Prevotella bryantii B14,Prevotella buccae ATCC 33574,Prevotella buccae D17,Prevotella buccalis ATCC 35310,Prevotella C561,Prevotella copri DSM 18205,Prevotella dentalis DSM 3688,Prevotella denticola CRIS 18C A,Prevotella denticola F0289,Prevotella disiens FB035 09AN,Prevotella histicola F0411,Prevotella intermedia 17,Prevotella maculosa OT 289,Prevotella marshii DSM 16973,Prevotella melaninogenica ATCC 25845,Prevotella melaninogenica D18,Prevotella micans F0438,Prevotella MSX73,Prevotella multiformis DSM 16608,Prevotella multisaccharivorax DSM 17128,Prevotella nigrescens ATCC 33563,Prevotella nigrescens F0103,Prevotella oral taxon 299 F0039,Prevotella oral taxon 302 F0323,Prevotella oral taxon 306 F0472,Prevotella oral taxon 317 F0108,Prevotella oral taxon 472 F0295,Prevotella oral taxon 473 F0040,Prevotella oralis ATCC 33269,Prevotella oris C735,Prevotella oris F0302,Prevotella oulorum F0390,Prevotella pallens ATCC 700821,Prevotella ruminicola 23,Prevotella saccharolytica F0055,Prevotella salivae DSM 15606,Prevotella stercorea DSM 18206,Prevotella tannerae ATCC 51259,Prevotella timonensis CRIS 5C B1,Prevotella veroralis F0319,Psychroflexus torquis ATCC 700755,Rhodothermus marinus DSM 4252,Rhodothermus marinus SG0 5JP17 172,Riemerella anatipestifer ATCC 11845 DSM 15868,Riemerella anatipestifer RA CH 1,Riemerella anatipestifer RA CH 2,Riemerella anatipestifer RA GD,Riemerella anatipestifer RA YM,Robiginitalea biformata HTCC2501,Runella slithyformis DSM 19594,Salinibacter ruber DSM 13855,Salinibacter ruber M8,Saprospira grandis DSM 2844,Saprospira grandis Lewin,Solitalea canadensis DSM 3403,Sphingobacterium 21,Sphingobacterium spiritivorum ATCC 33300,Sphingobacterium spiritivorum ATCC 33861,Spirosoma linguale DSM 74,Tannerella 6 1 58FAA CT1,Tannerella forsythia ATCC 43037,Weeksella virosa DSM 16922,Zobellia galactanivorans,Zunongwangia profunda SM A87,Bacteroidetes oral taxon 274 F0058,Elizabethkingia anophelis Ag1,Elizabethkingia anophelis R26,Flavobacteriaceae bacterium 3519 10,Flavobacteriaceae bacterium HQM9,Flavobacteriaceae bacterium S85,Flavobacteriales bacterium ALC 1,Flavobacteriales bacterium HTCC2170,Barnesiella intestinihominis YIT 11860,Bergeyella zoohelcum ATCC 43767,Bergeyella zoohelcum CCUG 30536,Bizionia argentinensis JUB59,Cecembia lonarensis LW9,Cesiribacter andamanensis AMV16,Dysgonomonas gadei ATCC BAA 286,Dysgonomonas mossii DSM 22836,Fibrisoma limi,Fulvivirga imtechensis AK7,Galbibacter ck I2 15,Gillisia CBA3202,Gillisia limnaea DSM 15749,Imtechella halotolerans K1,Indibacter alkaliphilus LW1,Joostella marina DSM 19592,Kordia algicida OT 1,Leeuwenhoekiella blandensis MED217,Marinilabilia AK2,Marinilabilia salmonicolor JCM 21150,Mariniradius saccharolyticus AK6,Mesoflavibacter zeaxanthinifaciens S86,Microscilla marina ATCC 23134,Mucilaginibacter paludis DSM 18603,Myroides injenensis M09 0166,Myroides odoratimimus CCUG 10230,Myroides odoratimimus CCUG 12901,Myroides odoratimimus CCUG 3837,Myroides odoratimimus CIP 101113,Myroides odoratimimus CIP 103059,Myroides odoratus DSM 2801,Niabella soli DSM 19437,Nitritalea halalkaliphila LW7,Paraprevotella clara YIT 11840,Paraprevotella xylaniphila YIT 11841,Pontibacter BAB1700,Oribacterium ACB1,Oribacterium ACB7,Chryseobacterium CF314,Chryseobacterium gleum ATCC 35910,Persicivirga dokdonensis DSW 6,Flavobacteria bacterium BAL38,Flavobacteria bacterium BBFL7,Flavobacteria bacterium MS024 2A,Flavobacteria bacterium MS024 3C,Oribacterium ACB8,Oribacterium oral taxon 078 F0262,Oribacterium oral taxon 108 F0425,Oribacterium sinus F0268 |
| Chlorobi | Chlorobaculum parvum NCIB 8327,Chlorobium chlorochromatii CaD3,Chlorobium ferrooxidans DSM 13031,Chlorobium limicola DSM 245,Chlorobium luteolum DSM 273,Chlorobium phaeobacteroides BS1,Chlorobium phaeobacteroides DSM 266,Chlorobium phaeovibrioides DSM 265,Chlorobium tepidum TLS,Chloroherpeton thalassium ATCC 35110,Ignavibacterium album JCM 16511,Melioribacter roseus P3M,Pelodictyon phaeoclathratiforme BU 1,Prosthecochloris aestuarii DSM 271 |
| Caldiserica | Caldisericum_exile_AZM16c01 |
| Chlamydiae | Chlamydia muridarum MopnTet14,Chlamydia muridarum Nigg,Chlamydia muridarum Weiss,Chlamydia pecorum P787,Chlamydia pecorum PV3056 3,Chlamydia pecorum W73,Chlamydia psittaci 01DC12,Chlamydia psittaci 84 55,Chlamydia psittaci GR9,Chlamydia psittaci M56,Chlamydia psittaci MN,Chlamydia psittaci VS225,Chlamydia psittaci WC,Chlamydia psittaci WS RT E30,Chlamydia trachomatis,Chlamydia trachomatis 434 Bu,Chlamydia trachomatis 6276,Chlamydia trachomatis 6276s,Chlamydia trachomatis 70,Chlamydia trachomatis 70s,Chlamydia trachomatis A2497,Chlamydia trachomatis A 363,Chlamydia trachomatis A 5291,Chlamydia trachomatis A HAR 13,Chlamydia trachomatis B Jali20 OT,Chlamydia trachomatis B TZ1A828 OT,Chlamydia trachomatis C TW 3,Chlamydia trachomatis D EC,Chlamydia trachomatis D LC,Chlamydia trachomatis D s 2923,Chlamydia trachomatis D SotonD1,Chlamydia trachomatis D SotonD5,Chlamydia trachomatis D SotonD6,Chlamydia trachomatis D UW 3 CX,Chlamydia trachomatis E 11023,Chlamydia trachomatis E 150,Chlamydia trachomatis E Bour,Chlamydia trachomatis E SotonE8,Chlamydia trachomatis E SW3,Chlamydia trachomatis F SW4,Chlamydia trachomatis F SW5,Chlamydia trachomatis G 11074,Chlamydia trachomatis G 11222,Chlamydia trachomatis G 9301,Chlamydia trachomatis G 9768,Chlamydia trachomatis G SotonG1,Chlamydia trachomatis Ia SotonIa1,Chlamydia trachomatis IU824,Chlamydia trachomatis IU888,Chlamydia trachomatis J 6276tet1,Chlamydia trachomatis K SotonK1,Chlamydia trachomatis L1 115,Chlamydia trachomatis L1 224,Chlamydia trachomatis L1 440 LN,Chlamydia trachomatis L2 25667R,Chlamydia trachomatis L2 434 Bu f,Chlamydia trachomatis L2 434 Bu i,Chlamydia trachomatis L2b 795,Chlamydia trachomatis L2b 8200 07,Chlamydia trachomatis L2b Ams1,Chlamydia trachomatis L2b Ams2,Chlamydia trachomatis L2b Ams3,Chlamydia trachomatis L2b Ams4,Chlamydia trachomatis L2b Ams5,Chlamydia trachomatis L2b Canada1,Chlamydia trachomatis L2b Canada2,Chlamydia trachomatis L2b CV204,Chlamydia trachomatis L2b UCH 1 proctitis,Chlamydia trachomatis L2b UCH 2,Chlamydia trachomatis L2c,Chlamydia trachomatis L2tet1,Chlamydia trachomatis L3 404 LN,Chlamydia trachomatis RC F 69,Chlamydia trachomatis RC F s 342,Chlamydia trachomatis RC F s 852,Chlamydia trachomatis RC J 943,Chlamydia trachomatis RC J 953,Chlamydia trachomatis RC J 966,Chlamydia trachomatis RC J 971,Chlamydia trachomatis RC J s 122,Chlamydia trachomatis RC L2 55,Chlamydia trachomatis RC L2 s 3,Chlamydia trachomatis RC L2 s 46,Chlamydia trachomatis Sweden2,Chlamydophila abortus LLG,Chlamydophila abortus S26 3,Chlamydophila caviae GPIC,Chlamydophila felis Fe C 56,Chlamydophila pecorum E58,Chlamydophila pneumoniae AR39,Chlamydophila pneumoniae CWL029,Chlamydophila pneumoniae J138,Chlamydophila pneumoniae LPCoLN,Chlamydophila pneumoniae TW 183,Chlamydophila psittaci 01DC11,Chlamydophila psittaci 02DC15,Chlamydophila psittaci 08DC60,Chlamydophila psittaci 6BC,Chlamydophila psittaci C19 98,Chlamydophila psittaci Cal10,Chlamydophila psittaci CP3,Chlamydophila psittaci Mat116,Chlamydophila psittaci NJ1,Chlamydophila psittaci RD1,Parachlamydia acanthamoebae Hall s coccus,Parachlamydia acanthamoebae UV7,Simkania negevensis Z,Waddlia chondrophila WSU 86 1044 |
| Lentisphaerae | Lentisphaera araneosa HTCC2155 |
| Verrucomicrobia | Akkermansia muciniphila ATCC BAA 835,Coraliomargarita akajimensis DSM 45221,Methylacidiphilum fumariolicum,Methylacidiphilum infernorum V4,Opitutus terrae PB90 1,Chthoniobacter flavus Ellin428,Verrucomicrobium spinosum DSM 4136,Opitutaceae bacterium TAV1,Opitutaceae bacterium TAV5,Verrucomicrobiae bacterium DG1235 |
| Chloroflexi | Ktedonobacter racemifer DSM 44963,Oscillochloris trichoides DG6,Nitrolancetus hollandicus Lb,Anaerolinea thermophila UNI 1,Caldilinea aerophila DSM 14535 NBRC 104270,Chloroflexus aggregans DSM 9485,Chloroflexus aurantiacus J 10 fl,Chloroflexus Y 400 fl,Dehalococcoides BAV1,Dehalococcoides CBDB1,Dehalococcoides ethenogenes 195,Dehalococcoides GT,Dehalococcoides mccartyi BTF08,Dehalococcoides mccartyi DCMB5,Dehalococcoides mccartyi GY50,Dehalococcoides VS,Dehalogenimonas lykanthroporepellens BL DC 9,Herpetosiphon aurantiacus DSM 785,Roseiflexus castenholzii DSM 13941,Roseiflexus RS 1,Sphaerobacter thermophilus DSM 20745,Thermomicrobium roseum DSM 5159, Thermobaculum terrenum ATCC BAA 798* |
| Chrysiogenetes | Desulfurispirillum indicum S5 |
| Cyanobacteria | Acaryochloris marina MBIC11017,Anabaena 90,Anabaena cylindrica PCC 7122,Anabaena variabilis ATCC 29413,Arthrospira platensis NIES 39,Calothrix PCC 6303,Calothrix PCC 7507,Chamaesiphon PCC 6605,Chroococcidiopsis thermalis PCC 7203,Crinalium epipsammum PCC 9333,Cyanobacterium PCC 10605,Cyanobacterium stanieri PCC 7202,Cyanobium gracile PCC 6307,Cyanobium PCC 7001,Cyanothece ATCC 51142,Cyanothece ATCC 51472,Cyanothece CCY0110,Cyanothece PCC 7424,Cyanothece PCC 7425,Cyanothece PCC 7822,Cyanothece PCC 8801,Cyanothece PCC 8802,Cylindrospermum stagnale PCC 7417,Dactylococcopsis salina PCC 8305,Geitlerinema PCC 7407,Gloeobacter JS,Gloeobacter violaceus PCC 7421,Gloeocapsa PCC 73106,Gloeocapsa PCC 7428,Halothece PCC 7418,Leptolyngbya PCC 6406,Leptolyngbya PCC 7375,Leptolyngbya PCC 7376,Leptolyngbya valderiana BDU 20041,Microcoleus chthonoplastes PCC 7420,Microcoleus PCC 7113,Microcoleus vaginatus FGP 2,Microcystis aeruginosa DIANCHI905,Microcystis aeruginosa NIES 843,Microcystis aeruginosa PCC 7941,Microcystis aeruginosa PCC 9432,Microcystis aeruginosa PCC 9443,Microcystis aeruginosa PCC 9701,Microcystis aeruginosa PCC 9717,Microcystis aeruginosa PCC 9806,Microcystis aeruginosa PCC 9807,Microcystis aeruginosa PCC 9808,Microcystis aeruginosa PCC 9809,Microcystis aeruginosa TAIHU98,Microcystis T1 4,Nostoc azollae 0708,Nostoc PCC 7107,Nostoc PCC 7120,Nostoc PCC 7524,Nostoc punctiforme PCC 73102,Oscillatoria acuminata PCC 6304,Oscillatoria PCC 6506,Oscillatoria PCC 7112,Pleurocapsa PCC 7327,Prochlorococcus marinus AS9601,Prochlorococcus marinus CCMP1375,Prochlorococcus marinus MIT 9202,Prochlorococcus marinus MIT 9211,Prochlorococcus marinus MIT 9215,Prochlorococcus marinus MIT 9301,Prochlorococcus marinus MIT 9303,Prochlorococcus marinus MIT 9312,Prochlorococcus marinus MIT 9313,Prochlorococcus marinus MIT 9515,Prochlorococcus marinus NATL1A,Prochlorococcus marinus NATL2A,Prochlorococcus marinus pastoris CCMP1986,Pseudanabaena biceps PCC 7429,Pseudanabaena PCC 7367,Rivularia PCC 7116,Stanieria cyanosphaera PCC 7437,Synechococcus BL107,Synechococcus CB0101,Synechococcus CB0205,Synechococcus CC9311,Synechococcus CC9605,Synechococcus CC9902,Synechococcus elongatus PCC 6301,Synechococcus elongatus PCC 7942,Synechococcus JA 2 3B a 2 13,Synechococcus JA 3 3Ab,Synechococcus PCC 6312,Synechococcus PCC 7002,Synechococcus PCC 7335,Synechococcus PCC 7502,Synechococcus RCC307,Synechococcus RS9916,Synechococcus RS9917,Synechococcus WH 5701,Synechococcus WH 7803,Synechococcus WH 7805,Synechococcus WH 8016,Synechococcus WH 8102,Synechococcus WH 8109,Synechocystis PCC 6803,Synechocystis PCC 6803 substr GT I,Synechocystis PCC 6803 substr PCC N,Synechocystis PCC 7509,Thermosynechococcus elongatus BP 1,Thermosynechococcus NK55,Trichodesmium erythraeum IMS101,Crocosphaera watsonii WH 0003,Crocosphaera watsonii WH 8501,Cylindrospermopsis raciborskii CS 505,Fischerella JSC 11,Lyngbya majuscula 3L,Lyngbya PCC 8106,Nodularia spumigena CCY9414,Oscillatoriales cyanobacterium JSC 12,Raphidiopsis brookii D9,Richelia intracellularis HH01,Richelia intracellularis HM01,Xenococcus PCC 7305 |
| Deferribacteres | Calditerrivibrio nitroreducens DSM 19672,Deferribacter desulfuricans SSM1,Denitrovibrio acetiphilus DSM 12809,Flexistipes sinusarabici DSM 4947 |
| Deinococcus-Thermus | Deinococcus deserti VCD115,Deinococcus geothermalis DSM 11300,Deinococcus gobiensis I 0,Deinococcus maricopensis DSM 21211,Deinococcus peraridilitoris DSM 19664,Deinococcus proteolyticus MRP,Deinococcus radiodurans R1,Marinithermus hydrothermalis DSM 14884,Meiothermus ruber DSM 1279,Meiothermus silvanus DSM 9946,Oceanithermus profundus DSM 14977,Thermus aquaticus Y51MC23,Thermus CCB US3 UF1,Thermus oshimai JL 2,Thermus RLM,Thermus scotoductus SA 01,Thermus thermophilus HB8,Thermus thermophilus JL 18,Thermus thermophilus SG0 5JP17 16,Truepera radiovictrix DSM 17093 |
| Dictyoglomi | Dictyoglomus thermophilum H 6 12, Dictyoglomus turgidum DSM 6724 |
| Elusimicrobia | Elusimicrobium minutum Pei191 |
| Acidobacteria | Acidobacterium capsulatum ATCC 51196,Acidobacterium MP5ACTX9,Candidatus Chloracidobacterium thermophilum B,Candidatus Koribacter versatilis Ellin345,Candidatus Solibacter usitatus Ellin6076,Granulicella mallensis MP5ACTX8,Terriglobus roseus DSM 18391,Terriglobus saanensis SP1PR4,Holophaga foetida DSM 6591 |
| Fibrobacteres | Fibrobacter succinogenes S85 |
| Firmicutes | Acetobacterium woodii DSM 1030,Acetohalobium arabaticum DSM 5501,Acidaminococcus fermentans DSM 20731,Acidaminococcus intestini RyC MR95,Aerococcus urinae ACS 120 V Col10a,Alicyclobacillus acidocaldarius DSM 446,Alicyclobacillus acidocaldarius Tc 4 1,Alkaliphilus metalliredigens QYMF,Alkaliphilus oremlandii OhILAs,Ammonifex degensii KC4,Amphibacillus xylanus NBRC 15112,Anaerococcus prevotii DSM 20548,Anoxybacillus flavithermus WK1,Bacillus 1NLA3E,Bacillus amyloliquefaciens CC178,Bacillus amyloliquefaciens DSM 7,Bacillus amyloliquefaciens FZB42,Bacillus amyloliquefaciens IT 45,Bacillus amyloliquefaciens LFB112,Bacillus amyloliquefaciens LL3,Bacillus amyloliquefaciens plantarum AS43 3,Bacillus amyloliquefaciens plantarum CAU B946,Bacillus amyloliquefaciens plantarum NAU B3,Bacillus amyloliquefaciens plantarum UCMB5033,Bacillus amyloliquefaciens plantarum UCMB5036,Bacillus amyloliquefaciens plantarum UCMB5113,Bacillus amyloliquefaciens plantarum YAU B9601 Y2,Bacillus amyloliquefaciens TA208,Bacillus amyloliquefaciens XH7,Bacillus amyloliquefaciens Y2,Bacillus anthracis Ames Ancestor,Bacillus anthracis A0248,Bacillus anthracis Ames,Bacillus anthracis CDC 684,Bacillus anthracis H9401,Bacillus anthracis Sterne,Bacillus atrophaeus 1942,Bacillus cellulosilyticus DSM 2522,Bacillus cereus 03BB102,Bacillus cereus AH187,Bacillus cereus AH820,Bacillus cereus ATCC 10987,Bacillus cereus ATCC 14579,Bacillus cereus B4264,Bacillus cereus biovar anthracis CI,Bacillus cereus E33L,Bacillus cereus F837 76,Bacillus cereus FRI 35,Bacillus cereus G9842,Bacillus cereus NC7401,Bacillus cereus Q1,Bacillus clausii KSM K16,Bacillus coagulans 2 6,Bacillus coagulans 36D1,Bacillus cytotoxicus NVH 391 98,Bacillus halodurans C 125,Bacillus infantis NRRL B 14911,Bacillus JS,Bacillus licheniformis 9945A,Bacillus licheniformis ATCC 14580,Bacillus licheniformis DSM 13 ATCC 14580,Bacillus megaterium DSM319,Bacillus megaterium QM B1551,Bacillus megaterium WSH 002,Bacillus pseudofirmus OF4,Bacillus pumilus SAFR 032,Bacillus selenitireducens MLS10,Bacillus subtilis 168,Bacillus subtilis 6051 HGW,Bacillus subtilis BAB 1,Bacillus subtilis BSn5,Bacillus subtilis BSP1,Bacillus subtilis natto BEST195,Bacillus subtilis PY79,Bacillus subtilis QB928,Bacillus subtilis RO NN 1,Bacillus subtilis spizizenii TU B 10,Bacillus subtilis spizizenii W23,Bacillus subtilis XF 1,Bacillus thuringiensis Al Hakam,Bacillus thuringiensis BMB171,Bacillus thuringiensis Bt407,Bacillus thuringiensis HD 771,Bacillus thuringiensis HD 789,Bacillus thuringiensis MC28,Bacillus thuringiensis serovar chinensis CT 43,Bacillus thuringiensis serovar finitimus YBT 020,Bacillus thuringiensis serovar IS5056,Bacillus thuringiensis serovar konkukian 97 27,Bacillus thuringiensis serovar kurstaki HD73,Bacillus thuringiensis YBT 1518,Bacillus toyonensis BCT 7112,Bacillus weihenstephanensis KBAB4,Brevibacillus agri BAB 2500,Brevibacillus BC25,Brevibacillus brevis NBRC 100599,Brevibacillus CF112,Brevibacillus laterosporus GI 9,Brevibacillus laterosporus LMG 15441,Butyrivibrio crossotus DSM 2876,Butyrivibrio fibrisolvens,Butyrivibrio proteoclasticus B316,Caldicellulosiruptor bescii DSM 6725,Caldicellulosiruptor hydrothermalis 108,Caldicellulosiruptor kristjanssonii 177R1B,Caldicellulosiruptor kronotskyensis 2002,Caldicellulosiruptor lactoaceticus 6A,Caldicellulosiruptor obsidiansis OB47,Caldicellulosiruptor owensensis OL,Caldicellulosiruptor saccharolyticus DSM 8903,Candidatus Arthromitus SFB 1,Candidatus Arthromitus SFB 2,Candidatus Arthromitus SFB 3,Candidatus Arthromitus SFB 4,Candidatus Arthromitus SFB 5,Candidatus Arthromitus SFB co,Candidatus Arthromitus SFB mouse Japan,Candidatus Arthromitus SFB mouse NYU,Candidatus Arthromitus SFB mouse SU,Candidatus Arthromitus SFB mouse Yit,Candidatus Arthromitus SFB rat Yit,Candidatus Desulforudis audaxviator MP104C,Carboxydothermus hydrogenoformans Z 2901,Carnobacterium 17 4,Carnobacterium AT7,Carnobacterium maltaromaticum ATCC 35586,Carnobacterium maltaromaticum LMA28,Carnobacterium WN1359,Clostridiales bacterium 1 7 47FAA,Clostridiales bacterium OBRC5 5,Clostridiales genomosp BVAB3 UPII9 5,Clostridium 7 2 43FAA,Clostridium 7 3 54FAA,Clostridium sticklandii,Clostridium acetobutylicum ATCC 824,Clostridium acetobutylicum DSM 1731,Clostridium acetobutylicum EA 2018,Clostridium acidurici 9a,Clostridium arbusti SL206,Clostridium asparagiforme DSM 15981,Clostridium autoethanogenum DSM 10061,Clostridium bartlettii DSM 16795,Clostridium beijerinckii NCIMB 8052,Clostridium BNL1100,Clostridium bolteae ATCC BAA 613,Clostridium botulinum A2 Kyoto,Clostridium botulinum A3 Loch Maree,Clostridium botulinum A ATCC 19397,Clostridium botulinum A ATCC 3502,Clostridium botulinum A Hall,Clostridium botulinum B1 Okra,Clostridium botulinum B Eklund 17B,Clostridium botulinum Ba4 657,Clostridium botulinum Bf,Clostridium botulinum BKT015925,Clostridium botulinum C Eklund,Clostridium botulinum C Stockholm,Clostridium botulinum CFSAN001627,Clostridium botulinum CFSAN001628,Clostridium botulinum D 1873,Clostridium botulinum E1 BoNT E Beluga,Clostridium botulinum E3 Alaska E43,Clostridium botulinum F 230613,Clostridium botulinum F Langeland,Clostridium botulinum H04402 065,Clostridium botulinum NCTC 2916,Clostridium butyricum 5521,Clostridium butyricum E4 BoNT E BL5262,Clostridium carboxidivorans P7,Clostridium celatum DSM 1785,Clostridium cellulolyticum H10,Clostridium cellulovorans 743B,Clostridium cf saccharolyticum K10,Clostridium citroniae WAL 17108,Clostridium clariflavum DSM 19732,Clostridium clostridioforme 2 1 49FAA,Clostridium D5,Clostridium difficile 002 P50 2011,Clostridium difficile 050 P50 2011,Clostridium difficile 630,Clostridium difficile 70 100 2010,Clostridium difficile ATCC 43255,Clostridium difficile BI1,Clostridium difficile CD196,Clostridium difficile CIP 107932,Clostridium difficile NAP07,Clostridium difficile NAP08,Clostridium difficile QCD 23m63,Clostridium difficile QCD 32g58,Clostridium difficile QCD 37x79,Clostridium difficile QCD 63q42,Clostridium difficile QCD 66c26,Clostridium difficile QCD 76w55,Clostridium difficile QCD 97b34,Clostridium difficile R20291,Clostridium DL VIII,Clostridium hathewayi DSM 13479,Clostridium hathewayi WAL 18680,Clostridium HGF2,Clostridium hiranonis DSM 13275,Clostridium hylemonae DSM 15053,Clostridium JC122,Clostridium kluyveri DSM 555,Clostridium kluyveri NBRC 12016,Clostridium L2 50,Clostridium lentocellum DSM 5427,Clostridium leptum DSM 753,Clostridium ljungdahlii DSM 13528,Clostridium M62 1,Clostridium Maddingley MBC34 26,Clostridium methylpentosum DSM 5476,Clostridium MSTE9,Clostridium nexile DSM 1787,Clostridium novyi NT,Clostridium papyrosolvens DSM 2782,Clostridium pasteurianum BC1,Clostridium pasteurianum DSM 525,Clostridium perfringens 13,Clostridium perfringens ATCC 13124,Clostridium perfringens B ATCC 3626,Clostridium perfringens C JGS1495,Clostridium perfringens CPE F4969,Clostridium perfringens D JGS1721,Clostridium perfringens E JGS1987,Clostridium perfringens F262,Clostridium perfringens NCTC 8239,Clostridium perfringens SM101,Clostridium perfringens WAL 14572,Clostridium phytofermentans ISDg,Clostridium ramosum DSM 1402,Clostridium saccharobutylicum DSM 13864,Clostridium saccharolyticum WM1,Clostridium saccharoperbutylacetonicum ATCC 27021,Clostridium scindens ATCC 35704,Clostridium spiroforme DSM 1552,Clostridium sporogenes ATCC 15579,Clostridium sporogenes PA 3679,Clostridium SS2 1,Clostridium stercorarium DSM 8532,Clostridium SY8519,Clostridium symbiosum WAL 14163,Clostridium symbiosum WAL 14673,Clostridium termitidis CT1112,Clostridium tetani 12124569,Clostridium tetani E88,Clostridium thermocellum AD2,Clostridium thermocellum ATCC 27405,Clostridium thermocellum DSM 1313,Clostridium thermocellum DSM 2360,Clostridium thermocellum JW20,Clostridium thermocellum YS,Clostridium ultunense Esp,Coprococcus ART55 1,Coprococcus catus GD 7,Coprococcus comes ATCC 27758,Coprococcus eutactus ATCC 27759,Coprothermobacter proteolyticus DSM 5265,Dehalobacter 11DCA,Dehalobacter CF,Desulfitobacterium dehalogenans ATCC 51507,Desulfitobacterium dichloroeliminans LMG P 21439,Desulfitobacterium hafniense DCB 2,Desulfitobacterium hafniense DP7,Desulfitobacterium hafniense Y51,Desulfitobacterium metallireducens DSM 15288,Desulfosporosinus acidiphilus SJ4,Desulfosporosinus meridiei DSM 13257,Desulfosporosinus orientis DSM 765,Desulfosporosinus OT,Desulfosporosinus youngiae DSM 17734,Desulfotomaculum acetoxidans DSM 771,Desulfotomaculum carboxydivorans CO 1 SRB,Desulfotomaculum gibsoniae DSM 7213,Desulfotomaculum hydrothermale Lam5 DSM 18033,Desulfotomaculum kuznetsovii DSM 6115,Desulfotomaculum nigrificans DSM 574,Desulfotomaculum reducens MI 1,Desulfotomaculum ruminis DSM 2154,Enterococcus 7L76,Enterococcus C1,Enterococcus casseliflavus ATCC 12755,Enterococcus casseliflavus EC10,Enterococcus casseliflavus EC20,Enterococcus casseliflavus EC30,Enterococcus durans FB129 CNAB 4,Enterococcus durans IPLA 655,Enterococcus faecalis 599,Enterococcus faecalis ARO1 DG,Enterococcus faecalis ATCC 29200,Enterococcus faecalis ATCC 29212,Enterococcus faecalis ATCC 4200,Enterococcus faecalis CH188,Enterococcus faecalis D32,Enterococcus faecalis D6,Enterococcus faecalis DAPTO 512,Enterococcus faecalis DAPTO 516,Enterococcus faecalis DS5,Enterococcus faecalis E1Sol,Enterococcus faecalis ERV103,Enterococcus faecalis ERV116,Enterococcus faecalis ERV129,Enterococcus faecalis ERV25,Enterococcus faecalis ERV31,Enterococcus faecalis ERV37,Enterococcus faecalis ERV41,Enterococcus faecalis ERV62,Enterococcus faecalis ERV63,Enterococcus faecalis ERV65,Enterococcus faecalis ERV68,Enterococcus faecalis ERV72,Enterococcus faecalis ERV73,Enterococcus faecalis ERV81,Enterococcus faecalis ERV85,Enterococcus faecalis ERV93,Enterococcus faecalis Fly1,Enterococcus faecalis HH22,Enterococcus faecalis HIP11704,Enterococcus faecalis JH1,Enterococcus faecalis M7,Enterococcus faecalis Merz96,Enterococcus faecalis OG1RF,Enterococcus faecalis OG1X,Enterococcus faecalis PC1 1,Enterococcus faecalis R508,Enterococcus faecalis R712,Enterococcus faecalis S613,Enterococcus faecalis Symbioflor 1,Enterococcus faecalis T1,Enterococcus faecalis T11,Enterococcus faecalis T2,Enterococcus faecalis T3,Enterococcus faecalis T8,Enterococcus faecalis TUSoD Ef11,Enterococcus faecalis TX0012,Enterococcus faecalis TX0017,Enterococcus faecalis TX0027,Enterococcus faecalis TX0031,Enterococcus faecalis TX0043,Enterococcus faecalis TX0102,Enterococcus faecalis TX0104,Enterococcus faecalis TX0109,Enterococcus faecalis TX0309A,Enterococcus faecalis TX0309B,Enterococcus faecalis TX0312,Enterococcus faecalis TX0411,Enterococcus faecalis TX0470,Enterococcus faecalis TX0630,Enterococcus faecalis TX0635,Enterococcus faecalis TX0645,Enterococcus faecalis TX0855,Enterococcus faecalis TX0860,Enterococcus faecalis TX1302,Enterococcus faecalis TX1322,Enterococcus faecalis TX1341,Enterococcus faecalis TX1342,Enterococcus faecalis TX1346,Enterococcus faecalis TX1467,Enterococcus faecalis TX2134,Enterococcus faecalis TX2137,Enterococcus faecalis TX2141,Enterococcus faecalis TX4000,Enterococcus faecalis TX4244,Enterococcus faecalis TX4248,Enterococcus faecalis V583,Enterococcus faecalis X98,Enterococcus faecium 1 141 733,Enterococcus faecium 1 230 933,Enterococcus faecium 1 231 408,Enterococcus faecium 1 231 410,Enterococcus faecium 1 231 501,Enterococcus faecium 1 231 502,Enterococcus faecium 503,Enterococcus faecium 504,Enterococcus faecium 505,Enterococcus faecium 506,Enterococcus faecium 509,Enterococcus faecium 510,Enterococcus faecium 511,Enterococcus faecium 513,Enterococcus faecium 514,Enterococcus faecium 515,Enterococcus faecium Aus0004,Enterococcus faecium Aus0085,Enterococcus faecium C1904,Enterococcus faecium C497,Enterococcus faecium C621,Enterococcus faecium C68,Enterococcus faecium Com12,Enterococcus faecium Com15,Enterococcus faecium D344SRF,Enterococcus faecium DO,Enterococcus faecium E0045,Enterococcus faecium E0120,Enterococcus faecium E0164,Enterococcus faecium E0269,Enterococcus faecium E0333,Enterococcus faecium E0679,Enterococcus faecium E0680,Enterococcus faecium E0688,Enterococcus faecium E1007,Enterococcus faecium E1039,Enterococcus faecium E1050,Enterococcus faecium E1071,Enterococcus faecium E1133,Enterococcus faecium E1162,Enterococcus faecium E1185,Enterococcus faecium E1258,Enterococcus faecium E1321,Enterococcus faecium E1392,Enterococcus faecium E1552,Enterococcus faecium E1573,Enterococcus faecium E1574,Enterococcus faecium E1575,Enterococcus faecium E1576,Enterococcus faecium E1578,Enterococcus faecium E1590,Enterococcus faecium E1604,Enterococcus faecium E1613,Enterococcus faecium E1620,Enterococcus faecium E1622,Enterococcus faecium E1623,Enterococcus faecium E1626,Enterococcus faecium E1627,Enterococcus faecium E1630,Enterococcus faecium E1634,Enterococcus faecium E1636,Enterococcus faecium E1644,Enterococcus faecium E1679,Enterococcus faecium E1731,Enterococcus faecium E1861,Enterococcus faecium E1904,Enterococcus faecium E1972,Enterococcus faecium E2039,Enterococcus faecium E2071,Enterococcus faecium E2134,Enterococcus faecium E2297,Enterococcus faecium E2369,Enterococcus faecium E2560,Enterococcus faecium E2620,Enterococcus faecium E2883,Enterococcus faecium E3083,Enterococcus faecium E3346,Enterococcus faecium E3548,Enterococcus faecium E417,Enterococcus faecium E4215,Enterococcus faecium E422,Enterococcus faecium E4389,Enterococcus faecium E4452,Enterococcus faecium E4453,Enterococcus faecium E6012,Enterococcus faecium E6045,Enterococcus faecium E980,Enterococcus faecium ERV1,Enterococcus faecium ERV102,Enterococcus faecium ERV161,Enterococcus faecium ERV165,Enterococcus faecium ERV168,Enterococcus faecium ERV26,Enterococcus faecium ERV38,Enterococcus faecium ERV69,Enterococcus faecium ERV99,Enterococcus faecium NRRL B 2354,Enterococcus faecium P1123,Enterococcus faecium P1137,Enterococcus faecium P1139,Enterococcus faecium P1140,Enterococcus faecium P1190,Enterococcus faecium P1986,Enterococcus faecium R446,Enterococcus faecium R494,Enterococcus faecium R496,Enterococcus faecium R497,Enterococcus faecium R499,Enterococcus faecium R501,Enterococcus faecium S447,Enterococcus faecium TC 6,Enterococcus faecium TX0082,Enterococcus faecium TX0133A,Enterococcus faecium TX0133a01,Enterococcus faecium TX0133a04,Enterococcus faecium TX0133B,Enterococcus faecium TX0133C,Enterococcus faecium TX1330,Enterococcus faecium TX1337RF,Enterococcus faecium U0317,Enterococcus faecium V689,Enterococcus gallinarum EG2,Enterococcus GMD1E,Enterococcus GMD2E,Enterococcus GMD3E,Enterococcus GMD4E,Enterococcus GMD5E,Enterococcus hirae ATCC 9790,Enterococcus italicus DSM 15952,Enterococcus mundtii QU 25,Enterococcus saccharolyticus 30 1,Erysipelothrix rhusiopathiae ATCC 19414,Erysipelothrix rhusiopathiae Fujisawa,Erysipelothrix rhusiopathiae SY1027,Ethanoligenens harbinense YUAN 3,Eubacterium 3 1 31,Eubacterium AS15,Eubacterium biforme DSM 3989,Eubacterium cellulosolvens 6,Eubacterium cylindroides T2 87,Eubacterium dolichum DSM 3991,Eubacterium eligens ATCC 27750,Eubacterium hadrum DSM 3319,Eubacterium hallii DSM 3353,Eubacterium infirmum F0142,Eubacterium limosum KIST612,Eubacterium rectale,Eubacterium rectale ATCC 33656,Eubacterium saburreum DSM 3986,Eubacterium saburreum F0468,Eubacterium saphenum ATCC 49989,Eubacterium siraeum,Eubacterium siraeum DSM 15702,Eubacterium siraeum V10Sc8a,Eubacterium ventriosum ATCC 27560,Eubacterium yurii margaretiae ATCC 43715,Exiguobacterium antarcticum B7,Exiguobacterium AT1b,Exiguobacterium MH3,Exiguobacterium sibiricum 255 15,Faecalibacterium cf prausnitzii KLE1255,Faecalibacterium prausnitzii,Faecalibacterium prausnitzii A2 165,Faecalibacterium prausnitzii L2 6,Faecalibacterium prausnitzii M21 2,Filifactor alocis ATCC 35896,Finegoldia magna ACS 171 V Col3,Finegoldia magna ATCC 29328,Finegoldia magna ATCC 53516,Finegoldia magna BVS033A4,Finegoldia magna SY403409CC001050417,Geobacillus C56 T3,Geobacillus G11MC16,Geobacillus HH01,Geobacillus JF8,Geobacillus kaustophilus HTA426,Geobacillus thermodenitrificans NG80 2,Geobacillus thermoglucosidans TNO 09 020,Geobacillus thermoglucosidasius C56 YS93,Geobacillus thermoleovorans CCB US3 UF5,Geobacillus WCH70,Geobacillus Y412MC52,Geobacillus Y412MC61,Geobacillus Y4 1MC1,Halanaerobium hydrogeniformans,Halanaerobium praevalens DSM 2228,Halanaerobium saccharolyticum DSM 6643,Halobacillus BAB 2008,Halobacillus halophilus DSM 2266,Halobacteroides halobius DSM 5150,Halothermothrix orenii H 168,Heliobacterium modesticaldum Ice1,Kyrpidia tusciae DSM 2912,Lactobacillus 66c,Lactobacillus 7 1 47FAA,Lactobacillus acidipiscis KCTC 13900,Lactobacillus acidophilus 30SC,Lactobacillus acidophilus ATCC 4796,Lactobacillus acidophilus La 14,Lactobacillus acidophilus NCFM,Lactobacillus amylolyticus DSM 11664,Lactobacillus amylovorus GRL1118,Lactobacillus amylovorus GRL 1112,Lactobacillus animalis KCTC 3501,Lactobacillus antri DSM 16041,Lactobacillus brevis ATCC 367,Lactobacillus brevis gravesensis ATCC 27305,Lactobacillus brevis KB290,Lactobacillus buchneri,Lactobacillus buchneri ATCC 11577,Lactobacillus buchneri NRRL B 30929,Lactobacillus casei 12A,Lactobacillus casei 21 1,Lactobacillus casei 32G,Lactobacillus casei A2 362,Lactobacillus casei ATCC 334,Lactobacillus casei BD II,Lactobacillus casei BL23,Lactobacillus casei CRF28,Lactobacillus casei LC2W,Lactobacillus casei Lc 10,Lactobacillus casei LOCK919,Lactobacillus casei Lpc 37,Lactobacillus casei M36,Lactobacillus casei T71499,Lactobacillus casei UCD174,Lactobacillus casei UW1,Lactobacillus casei UW4,Lactobacillus casei W56,Lactobacillus casei Zhang,Lactobacillus catenaformis OT 569,Lactobacillus coleohominis 101 4 CHN,Lactobacillus coryniformis CECT 5711,Lactobacillus coryniformis KCTC 3167,Lactobacillus coryniformis torquens KCTC 3535,Lactobacillus crispatus 125 2 CHN,Lactobacillus crispatus 214 1,Lactobacillus crispatus CTV 05,Lactobacillus crispatus FB049 03,Lactobacillus crispatus FB077 07,Lactobacillus crispatus JV V01,Lactobacillus crispatus MV 1A US,Lactobacillus crispatus MV 3A US,Lactobacillus crispatus ST1,Lactobacillus curvatus CRL 705,Lactobacillus delbrueckii bulgaricus 2038,Lactobacillus delbrueckii bulgaricus ATCC 11842,Lactobacillus delbrueckii bulgaricus ATCC BAA 365,Lactobacillus delbrueckii bulgaricus CNCM I 1519,Lactobacillus delbrueckii bulgaricus CNCM I 1632,Lactobacillus delbrueckii bulgaricus ND02,Lactobacillus delbrueckii bulgaricus PB2003 044 T3 4,Lactobacillus delbrueckii lactis DSM 20072,Lactobacillus equicursoris CIP 110162,Lactobacillus farciminis KCTC 3681,Lactobacillus fermentum 28 3 CHN,Lactobacillus fermentum ATCC 14931,Lactobacillus fermentum CECT 5716,Lactobacillus fermentum F6,Lactobacillus fermentum IFO 3956,Lactobacillus florum 2F,Lactobacillus fructivorans KCTC 3543,Lactobacillus gasseri 202 4,Lactobacillus gasseri 224 1,Lactobacillus gasseri ATCC 33323,Lactobacillus gasseri CECT 5714,Lactobacillus gasseri JV V03,Lactobacillus gasseri MV 22,Lactobacillus gastricus PS3,Lactobacillus gigeriorum CRBIP 24 85,Lactobacillus helveticus CNRZ32,Lactobacillus helveticus DPC 4571,Lactobacillus helveticus DSM 20075,Lactobacillus helveticus H10,Lactobacillus helveticus MTCC 5463,Lactobacillus helveticus R0052,Lactobacillus hilgardii ATCC 8290,Lactobacillus hominis CRBIP 24 179,Lactobacillus iners AB 1,Lactobacillus iners ATCC 55195,Lactobacillus iners DSM 13335,Lactobacillus iners LactinV 01V1 a,Lactobacillus iners LactinV 03V1 b,Lactobacillus iners LactinV 09V1 c,Lactobacillus iners LactinV 11V1 d,Lactobacillus iners LEAF 2052A d,Lactobacillus iners LEAF 2053A b,Lactobacillus iners LEAF 2062A h1,Lactobacillus iners LEAF 3008A a,Lactobacillus iners SPIN 1401G,Lactobacillus iners SPIN 2503V10 D,Lactobacillus iners UPII 143 D,Lactobacillus iners UPII 60 B,Lactobacillus jensenii 1153,Lactobacillus jensenii 115 3 CHN,Lactobacillus jensenii 269 3,Lactobacillus jensenii 27 2 CHN,Lactobacillus jensenii JV V16,Lactobacillus jensenii SJ 7A US,Lactobacillus johnsonii ATCC 33200,Lactobacillus johnsonii DPC 6026,Lactobacillus johnsonii FI9785,Lactobacillus johnsonii N6 2,Lactobacillus johnsonii NCC 533,Lactobacillus johnsonii pf01,Lactobacillus kefiranofaciens ZW3,Lactobacillus kisonensis F0435,Lactobacillus malefermentans KCTC 3548,Lactobacillus mali KCTC 3596,Lactobacillus mali KCTC 3596 DSM 20444,Lactobacillus mucosae LM1,Lactobacillus oris F0423,Lactobacillus oris PB013 T2 3,Lactobacillus paracasei 8700 2,Lactobacillus paracasei ATCC 25302,Lactobacillus parafarraginis F0439,Lactobacillus pasteurii CRBIP 24 76,Lactobacillus pentosus KCA1,Lactobacillus plantarum 16,Lactobacillus plantarum ATCC 14917,Lactobacillus plantarum JDM1,Lactobacillus plantarum NC8,Lactobacillus plantarum P8,Lactobacillus plantarum ST III,Lactobacillus plantarum UCMA 3037,Lactobacillus plantarum WCFS1,Lactobacillus plantarum ZJ316,Lactobacillus reuteri 100 23,Lactobacillus reuteri ATCC 53608,Lactobacillus reuteri CF48 3A,Lactobacillus reuteri DSM 20016,Lactobacillus reuteri I5007,Lactobacillus reuteri JCM 1112,Lactobacillus reuteri MM2 3,Lactobacillus reuteri MM4 1A,Lactobacillus reuteri SD2112,Lactobacillus reuteri TD1,Lactobacillus rhamnosus ATCC 21052,Lactobacillus rhamnosus ATCC 8530,Lactobacillus rhamnosus GG,Lactobacillus rhamnosus HN001,Lactobacillus rhamnosus Lc 705,Lactobacillus rhamnosus LMS2 1,Lactobacillus rhamnosus LOCK900,Lactobacillus rhamnosus LOCK908,Lactobacillus rhamnosus LRHMDP2,Lactobacillus rhamnosus LRHMDP3,Lactobacillus rhamnosus MTCC 5462,Lactobacillus rhamnosus R0011,Lactobacillus ruminis ATCC 25644,Lactobacillus ruminis ATCC 27782,Lactobacillus ruminis SPM0211,Lactobacillus sakei 23K,Lactobacillus salivarius ACS 116 V Col5a,Lactobacillus salivarius ATCC 11741,Lactobacillus salivarius CECT 5713,Lactobacillus salivarius GJ 24,Lactobacillus salivarius NIAS840,Lactobacillus salivarius SMXD51,Lactobacillus salivarius UCC118,Lactobacillus sanfranciscensis TMW 1 1304,Lactobacillus suebicus KCTC 3549,Lactobacillus ultunensis DSM 16047,Lactobacillus vaginalis ATCC 49540,Lactobacillus versmoldensis KCTC 3814,Lactobacillus vini DSM 20605,Lactobacillus zeae KCTC 3804,Lactococcus garvieae ATCC 49156,Lactococcus garvieae DCC43,Lactococcus garvieae IPLA 31405,Lactococcus garvieae Lg2,Lactococcus lactis cremoris A76,Lactococcus lactis cremoris CNCM I 1631,Lactococcus lactis cremoris KW2,Lactococcus lactis cremoris MG1363,Lactococcus lactis cremoris NZ9000,Lactococcus lactis cremoris SK11,Lactococcus lactis cremoris UC509 9,Lactococcus lactis CV56,Lactococcus lactis Il1403,Lactococcus lactis IO 1,Lactococcus lactis KF147,Lactococcus lactis KLDS 4 0325,Lactococcus raffinolactis 4877,Leuconostoc argentinum KCTC 3773,Leuconostoc C2,Leuconostoc carnosum JB16,Leuconostoc citreum KM20,Leuconostoc citreum LBAE C10,Leuconostoc citreum LBAE C11,Leuconostoc citreum LBAE E16,Leuconostoc fallax KCTC 3537,Leuconostoc gasicomitatum LMG 18811,Leuconostoc gelidum JB7,Leuconostoc gelidum KCTC 3527,Leuconostoc inhae KCTC 3774,Leuconostoc kimchii IMSNU 11154,Leuconostoc lactis KCTC 3528,Leuconostoc mesenteroides ATCC 8293,Leuconostoc mesenteroides cremoris ATCC 19254,Leuconostoc mesenteroides J18,Leuconostoc pseudomesenteroides 4882,Leuconostoc pseudomesenteroides KCTC 3652,Listeria grayi DSM 20601,Listeria innocua ATCC 33091,Listeria innocua Clip11262,Listeria innocua FSL J1 023,Listeria innocua FSL S4 378,Listeria ivanovii FSL F6 596,Listeria ivanovii PAM 55,Listeria marthii FSL S4 120,Listeria monocytogenes,Listeria monocytogenes 07PF0776,Listeria monocytogenes 08 5923,Listeria monocytogenes 10403S,Listeria monocytogenes ATCC 19117,Listeria monocytogenes Clip80459,Listeria monocytogenes EGD,Listeria monocytogenes EGD e,Listeria monocytogenes F6900,Listeria monocytogenes Finland 1998,Listeria monocytogenes FSL F2 208,Listeria monocytogenes FSL F2 515,Listeria monocytogenes FSL J1 175,Listeria monocytogenes FSL J1 194,Listeria monocytogenes FSL J1 208,Listeria monocytogenes FSL J2 003,Listeria monocytogenes FSL J2 064,Listeria monocytogenes FSL J2 071,Listeria monocytogenes FSL N1 017,Listeria monocytogenes FSL N3 165,Listeria monocytogenes FSL R2 503,Listeria monocytogenes FSL R2 561,Listeria monocytogenes HCC23,Listeria monocytogenes HPB2262,Listeria monocytogenes J0161,Listeria monocytogenes J1816,Listeria monocytogenes J1 220,Listeria monocytogenes J2818,Listeria monocytogenes L312,Listeria monocytogenes La111,Listeria monocytogenes LO28,Listeria monocytogenes M7,Listeria monocytogenes N53 1,Listeria monocytogenes Scott A,Listeria monocytogenes serotype 1 2a F6854,Listeria monocytogenes serotype 1 2b SLCC2755,Listeria monocytogenes serotype 4a L99,Listeria monocytogenes serotype 4b F2365,Listeria monocytogenes serotype 4b H7858,Listeria monocytogenes serotype 4b LL195,Listeria monocytogenes serotype 7 SLCC2482,Listeria monocytogenes SLCC2372,Listeria monocytogenes SLCC2376,Listeria monocytogenes SLCC2378,Listeria monocytogenes SLCC2479,Listeria monocytogenes SLCC2540,Listeria monocytogenes SLCC5850,Listeria monocytogenes SLCC7179,Listeria seeligeri FSL N1 067,Listeria seeligeri FSL S4 171,Listeria seeligeri serovar 1 2b SLCC3954,Listeria welshimeri serovar 6b SLCC5334,Lysinibacillus fusiformis ZC1,Lysinibacillus sphaericus C3 41,Macrococcus caseolyticus JCSC5402,Mahella australiensis 50 1 BON,Megamonas funiformis YIT 11815,Megamonas hypermegale,Megasphaera elsdenii DSM 20460,Megasphaera genomosp type 1 28L,Megasphaera micronuciformis F0359,Megasphaera UPII 135 E,Megasphaera UPII 199 6,Melissococcus plutonius ATCC 35311,Melissococcus plutonius DAT561,Moorella thermoacetica ATCC 39073,Natranaerobius thermophilus JW NM WN LF,Oceanobacillus iheyensis HTE831,Oceanobacillus Ndiop,Oenococcus kitaharae DSM 17330,Oenococcus oeni ATCC BAA 1163,Oenococcus oeni AWRIB202,Oenococcus oeni AWRIB304,Oenococcus oeni AWRIB318,Oenococcus oeni AWRIB418,Oenococcus oeni AWRIB419,Oenococcus oeni AWRIB422,Oenococcus oeni AWRIB429,Oenococcus oeni AWRIB548,Oenococcus oeni AWRIB553,Oenococcus oeni AWRIB568,Oenococcus oeni AWRIB576,Oenococcus oeni DSM 20252 AWRIB129,Oenococcus oeni PSU 1,Oscillibacter valericigenes,Paenibacillus Aloe 11,Paenibacillus alvei DSM 29,Paenibacillus curdlanolyticus YK9,Paenibacillus dendritiformis C454,Paenibacillus elgii B69,Paenibacillus HGF5,Paenibacillus HGF7,Paenibacillus JC66,Paenibacillus JDR 2,Paenibacillus lactis 154,Paenibacillus larvae 04 309,Paenibacillus mucilaginosus 3016,Paenibacillus mucilaginosus K02,Paenibacillus mucilaginosus KNP414,Paenibacillus oral taxon 786 D14,Paenibacillus peoriae KCTC 3763,Paenibacillus polymyxa CR1,Paenibacillus polymyxa E681,Paenibacillus polymyxa M1,Paenibacillus polymyxa SC2,Paenibacillus popilliae ATCC 14706,Paenibacillus terrae HPL 003,Paenibacillus vortex V453,Paenibacillus Y412MC10,Pediococcus acidilactici 7 4,Pediococcus acidilactici DSM 20284,Pediococcus acidilactici MA18 5M,Pediococcus claussenii ATCC BAA 344,Pediococcus lolii NGRI 0510Q,Pediococcus pentosaceus ATCC 25745,Pediococcus pentosaceus IE 3,Pediococcus pentosaceus SL4,Pelotomaculum thermopropionicum SI,Roseburia hominis A2 183,Roseburia intestinalis,Roseburia intestinalis L1 82,Roseburia intestinalis XB6B4,Roseburia inulinivorans DSM 16841,Ruminococcus,Ruminococcus 5 1 39BFAA,Ruminococcus albus 7,Ruminococcus albus 8,Ruminococcus bromii,Ruminococcus champanellensis 18P13,Ruminococcus flavefaciens FD 1,Ruminococcus gnavus ATCC 29149,Ruminococcus lactaris ATCC 29176,Ruminococcus obeum,Ruminococcus obeum ATCC 29174,Ruminococcus torques,Ruminococcus torques ATCC 27756,Selenomonas artemidis F0399,Selenomonas CM52,Selenomonas F0473,Selenomonas flueggei ATCC 43531,Selenomonas FOBRC6,Selenomonas FOBRC9,Selenomonas infelix ATCC 43532,Selenomonas noxia ATCC 43541,Selenomonas noxia F0398,Selenomonas oral taxon 137 F0430,Selenomonas oral taxon 138 F0429,Selenomonas oral taxon 149 67H29BP,Selenomonas ruminantium lactilytica TAM6421,Selenomonas sputigena ATCC 35185,Solibacillus silvestris StLB046,Staphylococcus arlettae CVD059,Staphylococcus aureus,Staphylococcus aureus 04 02981,Staphylococcus aureus 08BA02176,Staphylococcus aureus 118,Staphylococcus aureus 11819 97,Staphylococcus aureus 132,Staphylococcus aureus 16K,Staphylococcus aureus 21172,Staphylococcus aureus 21178,Staphylococcus aureus 21189,Staphylococcus aureus 21193,Staphylococcus aureus 21194,Staphylococcus aureus 21195,Staphylococcus aureus 21196,Staphylococcus aureus 21200,Staphylococcus aureus 21201,Staphylococcus aureus 21202,Staphylococcus aureus 21209,Staphylococcus aureus 21232,Staphylococcus aureus 21235,Staphylococcus aureus 21236,Staphylococcus aureus 21252,Staphylococcus aureus 21259,Staphylococcus aureus 21262,Staphylococcus aureus 21264,Staphylococcus aureus 21266,Staphylococcus aureus 21269,Staphylococcus aureus 21272,Staphylococcus aureus 21282,Staphylococcus aureus 21283,Staphylococcus aureus 21305,Staphylococcus aureus 21310,Staphylococcus aureus 21318,Staphylococcus aureus 21331,Staphylococcus aureus 21333,Staphylococcus aureus 21334,Staphylococcus aureus 21340,Staphylococcus aureus 21342,Staphylococcus aureus 21343,Staphylococcus aureus 21345,Staphylococcus aureus 55 2053,Staphylococcus aureus 58 424,Staphylococcus aureus 65 1322,Staphylococcus aureus 6850,Staphylococcus aureus 68 397,Staphylococcus aureus 71193,Staphylococcus aureus 930918 3,Staphylococcus aureus A017934 97,Staphylococcus aureus A10102,Staphylococcus aureus A5937,Staphylococcus aureus A5948,Staphylococcus aureus A6224,Staphylococcus aureus A6300,Staphylococcus aureus A8115,Staphylococcus aureus A8117,Staphylococcus aureus A8796,Staphylococcus aureus A8819,Staphylococcus aureus A9299,Staphylococcus aureus A9635,Staphylococcus aureus A9719,Staphylococcus aureus A9754,Staphylococcus aureus A9763,Staphylococcus aureus A9765,Staphylococcus aureus A9781,Staphylococcus aureus ATCC 51811,Staphylococcus aureus ATCC BAA 39,Staphylococcus aureus Bmb9393,Staphylococcus aureus Btn1260,Staphylococcus aureus C101,Staphylococcus aureus C160,Staphylococcus aureus C427,Staphylococcus aureus CC45,Staphylococcus aureus CF Marseille,Staphylococcus aureus CGS00,Staphylococcus aureus CGS01,Staphylococcus aureus CGS03,Staphylococcus aureus CIG1057,Staphylococcus aureus CIG1096,Staphylococcus aureus CIG1114,Staphylococcus aureus CIG1150,Staphylococcus aureus CIG1165,Staphylococcus aureus CIG1176,Staphylococcus aureus CIG1213,Staphylococcus aureus CIG1214,Staphylococcus aureus CIG1233,Staphylococcus aureus CIG1242,Staphylococcus aureus CIG1267,Staphylococcus aureus CIG149,Staphylococcus aureus CIG1500,Staphylococcus aureus CIG1524,Staphylococcus aureus CIG1605,Staphylococcus aureus CIG1612,Staphylococcus aureus CIG1750,Staphylococcus aureus CIG1769,Staphylococcus aureus CIG1770,Staphylococcus aureus CIG1835,Staphylococcus aureus CIG2018,Staphylococcus aureus CIG290,Staphylococcus aureus CIG547,Staphylococcus aureus CIGC128,Staphylococcus aureus CIGC340D,Staphylococcus aureus CIGC341D,Staphylococcus aureus CIGC345D,Staphylococcus aureus CIGC348,Staphylococcus aureus CIGC93,Staphylococcus aureus CM05,Staphylococcus aureus CN1,Staphylococcus aureus CO 23,Staphylococcus aureus COL,Staphylococcus aureus D139,Staphylococcus aureus D30,Staphylococcus aureus DR10,Staphylococcus aureus DSM 20231,Staphylococcus aureus E1410,Staphylococcus aureus ECT R 2,Staphylococcus aureus ED133,Staphylococcus aureus ED98,Staphylococcus aureus EMRSA16,Staphylococcus aureus GR1,Staphylococcus aureus H19,Staphylococcus aureus HO 5096 0412,Staphylococcus aureus IS 105,Staphylococcus aureus IS 111,Staphylococcus aureus IS 122,Staphylococcus aureus IS 125,Staphylococcus aureus IS 157,Staphylococcus aureus IS 160,Staphylococcus aureus IS 189,Staphylococcus aureus IS 24,Staphylococcus aureus IS 250,Staphylococcus aureus IS 3,Staphylococcus aureus IS 55,Staphylococcus aureus IS 88,Staphylococcus aureus IS 91,Staphylococcus aureus IS 99,Staphylococcus aureus IS K,Staphylococcus aureus IS M,Staphylococcus aureus JH1,Staphylococcus aureus JH9,Staphylococcus aureus JKD6008,Staphylococcus aureus JKD6009,Staphylococcus aureus JKD6159,Staphylococcus aureus KLT6,Staphylococcus aureus KT 314250,Staphylococcus aureus KT Y21,Staphylococcus aureus LCT SA112,Staphylococcus aureus LGA251,Staphylococcus aureus M013,Staphylococcus aureus M0719,Staphylococcus aureus M0769,Staphylococcus aureus M0770,Staphylococcus aureus M1,Staphylococcus aureus M1015,Staphylococcus aureus M1451,Staphylococcus aureus M809,Staphylococcus aureus M876,Staphylococcus aureus M899,Staphylococcus aureus MN8,Staphylococcus aureus MR1,Staphylococcus aureus MRSA131,Staphylococcus aureus MRSA177,Staphylococcus aureus MRSA252,Staphylococcus aureus MSHR1132,Staphylococcus aureus MSSA476,Staphylococcus aureus Mu3,Staphylococcus aureus Mu50,Staphylococcus aureus Mu50 omega,Staphylococcus aureus MW2,Staphylococcus aureus N315,Staphylococcus aureus NCTC 8325,Staphylococcus aureus Newbould 305,Staphylococcus aureus Newman,Staphylococcus aureus NN50,Staphylococcus aureus NN54,Staphylococcus aureus O11,Staphylococcus aureus O46,Staphylococcus aureus PM1,Staphylococcus aureus RF122,Staphylococcus aureus RN4220,Staphylococcus aureus SA40,Staphylococcus aureus SA957,Staphylococcus aureus ST228 10388,Staphylococcus aureus ST228 10497,Staphylococcus aureus ST228 15532,Staphylococcus aureus ST228 16035,Staphylococcus aureus ST228 18412,Staphylococcus aureus ST398,Staphylococcus aureus T0131,Staphylococcus aureus TCH130,Staphylococcus aureus TCH60,Staphylococcus aureus TCH70,Staphylococcus aureus TW20,Staphylococcus aureus USA300 FPR3757,Staphylococcus aureus USA300 TCH1516,Staphylococcus aureus USA300 TCH959,Staphylococcus aureus VC40,Staphylococcus aureus VCU006,Staphylococcus aureus VRS1,Staphylococcus aureus VRS10,Staphylococcus aureus VRS11a,Staphylococcus aureus VRS11b,Staphylococcus aureus VRS2,Staphylococcus aureus VRS3a,Staphylococcus aureus VRS4,Staphylococcus aureus VRS5,Staphylococcus aureus VRS6,Staphylococcus aureus VRS7,Staphylococcus aureus VRS8,Staphylococcus aureus VRS9,Staphylococcus aureus WBG10049,Staphylococcus aureus WW2703 97,Staphylococcus aureus Z172,Staphylococcus capitis SK14,Staphylococcus capitis VCU116,Staphylococcus caprae C87,Staphylococcus carnosus TM300,Staphylococcus epidermidis 14 1 R1 SE,Staphylococcus epidermidis ATCC 12228,Staphylococcus epidermidis AU12 03,Staphylococcus epidermidis BCM HMP0060,Staphylococcus epidermidis BVS058A4,Staphylococcus epidermidis FRI909,Staphylococcus epidermidis M23864 W1,Staphylococcus epidermidis M23864 W2 grey,Staphylococcus epidermidis NIH04003,Staphylococcus epidermidis NIH04008,Staphylococcus epidermidis NIH05001,Staphylococcus epidermidis NIH05003,Staphylococcus epidermidis NIH05005,Staphylococcus epidermidis NIH051475,Staphylococcus epidermidis NIH051668,Staphylococcus epidermidis NIH06004,Staphylococcus epidermidis NIH08001,Staphylococcus epidermidis NIHLM001,Staphylococcus epidermidis NIHLM003,Staphylococcus epidermidis NIHLM008,Staphylococcus epidermidis NIHLM015,Staphylococcus epidermidis NIHLM018,Staphylococcus epidermidis NIHLM020,Staphylococcus epidermidis NIHLM021,Staphylococcus epidermidis NIHLM023,Staphylococcus epidermidis NIHLM031,Staphylococcus epidermidis NIHLM037,Staphylococcus epidermidis NIHLM039,Staphylococcus epidermidis NIHLM040,Staphylococcus epidermidis NIHLM049,Staphylococcus epidermidis NIHLM053,Staphylococcus epidermidis NIHLM057,Staphylococcus epidermidis NIHLM061,Staphylococcus epidermidis NIHLM067,Staphylococcus epidermidis NIHLM070,Staphylococcus epidermidis NIHLM087,Staphylococcus epidermidis NIHLM088,Staphylococcus epidermidis NIHLM095,Staphylococcus epidermidis RP62A,Staphylococcus epidermidis SK135,Staphylococcus epidermidis VCU028,Staphylococcus epidermidis VCU037,Staphylococcus epidermidis VCU041,Staphylococcus epidermidis VCU045,Staphylococcus epidermidis VCU057,Staphylococcus epidermidis VCU065,Staphylococcus epidermidis VCU071,Staphylococcus epidermidis VCU081,Staphylococcus epidermidis VCU105,Staphylococcus epidermidis VCU109,Staphylococcus epidermidis VCU117,Staphylococcus epidermidis VCU118,Staphylococcus epidermidis VCU120,Staphylococcus epidermidis VCU123,Staphylococcus epidermidis VCU125,Staphylococcus epidermidis VCU126,Staphylococcus epidermidis VCU127,Staphylococcus epidermidis VCU128,Staphylococcus epidermidis VCU129,Staphylococcus epidermidis VCU144,Staphylococcus epidermidis W23144,Staphylococcus equorum Mu2,Staphylococcus haemolyticus JCSC1435,Staphylococcus hominis C80,Staphylococcus hominis SK119,Staphylococcus hominis VCU122,Staphylococcus lugdunensis ACS 027 V Sch2,Staphylococcus lugdunensis HKU09 01,Staphylococcus lugdunensis M23590,Staphylococcus lugdunensis N920143,Staphylococcus lugdunensis VCU139,Staphylococcus OJ82,Staphylococcus pasteuri SP1,Staphylococcus pettenkoferi VCU012,Staphylococcus pseudintermedius ED99,Staphylococcus pseudintermedius HKU10 03,Staphylococcus saprophyticus ATCC 15305,Staphylococcus saprophyticus KACC 16562,Staphylococcus simiae CCM 7213,Staphylococcus simulans ACS 120 V Sch1,Staphylococcus warneri L37603,Staphylococcus warneri SG1,Staphylococcus warneri VCU121,Streptococcus 2 1 36FAA,Streptococcus agalactiae 09mas018883,Streptococcus agalactiae 18RS21,Streptococcus agalactiae 2603V R,Streptococcus agalactiae 2 22,Streptococcus agalactiae 515,Streptococcus agalactiae A909,Streptococcus agalactiae ATCC 13813,Streptococcus agalactiae BSU108,Streptococcus agalactiae BSU165,Streptococcus agalactiae BSU167,Streptococcus agalactiae BSU178,Streptococcus agalactiae BSU188,Streptococcus agalactiae BSU247,Streptococcus agalactiae BSU442,Streptococcus agalactiae BSU450,Streptococcus agalactiae BSU92,Streptococcus agalactiae BSU96,Streptococcus agalactiae CCUG 19094,Streptococcus agalactiae CCUG 24810,Streptococcus agalactiae CCUG 25532,Streptococcus agalactiae CCUG 37430,Streptococcus agalactiae CCUG 37737,Streptococcus agalactiae CCUG 37738,Streptococcus agalactiae CCUG 37741,Streptococcus agalactiae CCUG 37742,Streptococcus agalactiae CCUG 38383,Streptococcus agalactiae CCUG 39096 A,Streptococcus agalactiae CCUG 44077,Streptococcus agalactiae CCUG 49087,Streptococcus agalactiae CF01173,Streptococcus agalactiae CJB111,Streptococcus agalactiae COH1,Streptococcus agalactiae FSL C1 494,Streptococcus agalactiae FSL F2 343,Streptococcus agalactiae FSL S3 003,Streptococcus agalactiae FSL S3 026,Streptococcus agalactiae FSL S3 277,Streptococcus agalactiae FSL S3 337,Streptococcus agalactiae FSL S3 501,Streptococcus agalactiae FSL S3 603,Streptococcus agalactiae GB00002,Streptococcus agalactiae GB00003,Streptococcus agalactiae GB00012,Streptococcus agalactiae GB00013,Streptococcus agalactiae GB00018,Streptococcus agalactiae GB00020,Streptococcus agalactiae GB00082,Streptococcus agalactiae GB00083,Streptococcus agalactiae GB00084,Streptococcus agalactiae GB00092,Streptococcus agalactiae GB00097,Streptococcus agalactiae GB00111,Streptococcus agalactiae GB00112,Streptococcus agalactiae GB00115,Streptococcus agalactiae GB00174,Streptococcus agalactiae GB00190,Streptococcus agalactiae GB00202,Streptococcus agalactiae GB00206,Streptococcus agalactiae GB00219,Streptococcus agalactiae GB00226,Streptococcus agalactiae GB00241,Streptococcus agalactiae GB00245,Streptococcus agalactiae GB00247,Streptococcus agalactiae GB00264,Streptococcus agalactiae GB00279,Streptococcus agalactiae GB00300,Streptococcus agalactiae GB00535,Streptococcus agalactiae GB00543,Streptococcus agalactiae GB00548,Streptococcus agalactiae GB00555,Streptococcus agalactiae GB00557,Streptococcus agalactiae GB00561,Streptococcus agalactiae GB00588,Streptococcus agalactiae GB00601,Streptococcus agalactiae GB00614,Streptococcus agalactiae GB00640,Streptococcus agalactiae GB00651,Streptococcus agalactiae GB00653,Streptococcus agalactiae GB00654,Streptococcus agalactiae GB00663,Streptococcus agalactiae GB00679,Streptococcus agalactiae GB00864,Streptococcus agalactiae GB00865,Streptococcus agalactiae GB00867,Streptococcus agalactiae GB00874,Streptococcus agalactiae GB00884,Streptococcus agalactiae GB00887,Streptococcus agalactiae GB00888,Streptococcus agalactiae GB00891,Streptococcus agalactiae GB00893,Streptococcus agalactiae GB00897,Streptococcus agalactiae GB00899,Streptococcus agalactiae GB00901,Streptococcus agalactiae GB00904,Streptococcus agalactiae GB00909,Streptococcus agalactiae GB00911,Streptococcus agalactiae GB00914,Streptococcus agalactiae GB00922,Streptococcus agalactiae GB00923,Streptococcus agalactiae GB00924,Streptococcus agalactiae GB00929,Streptococcus agalactiae GB00932,Streptococcus agalactiae GB00933,Streptococcus agalactiae GB00947,Streptococcus agalactiae GB00955,Streptococcus agalactiae GB00959,Streptococcus agalactiae GB00975,Streptococcus agalactiae GB00984,Streptococcus agalactiae GB00986,Streptococcus agalactiae GB00992,Streptococcus agalactiae GD201008 001,Streptococcus agalactiae Gottschalk 1003A,Streptococcus agalactiae Gottschalk 1005B,Streptococcus agalactiae Gottschalk 992B,Streptococcus agalactiae H36B,Streptococcus agalactiae ILRI005,Streptococcus agalactiae ILRI112,Streptococcus agalactiae LADL 90 503,Streptococcus agalactiae LDS 617,Streptococcus agalactiae LDS 628,Streptococcus agalactiae LMG 14608,Streptococcus agalactiae LMG 14609,Streptococcus agalactiae LMG 14838,Streptococcus agalactiae LMG 15085,Streptococcus agalactiae LMG 15091,Streptococcus agalactiae LMG 15092,Streptococcus agalactiae LMG 15093,Streptococcus agalactiae LMG 15094,Streptococcus agalactiae LMG 15095,Streptococcus agalactiae MRI Z1 012,Streptococcus agalactiae MRI Z1 211,Streptococcus agalactiae MRI Z1 212,Streptococcus agalactiae MRI Z1 213,Streptococcus agalactiae MRI Z1 214,Streptococcus agalactiae MRI Z1 216,Streptococcus agalactiae NEM316,Streptococcus agalactiae SA20 06,Streptococcus agalactiae SS1014,Streptococcus agalactiae SS1219,Streptococcus agalactiae STIR CD 09,Streptococcus agalactiae STIR CD 13,Streptococcus agalactiae STIR CD 17,Streptococcus agalactiae STIR CD 21,Streptococcus agalactiae STIR CD 22,Streptococcus agalactiae STIR CD 23,Streptococcus agalactiae STIR CD 24,Streptococcus agalactiae STIR CD 27,Streptococcus agalactiae STIR CD 28,Streptococcus agalactiae ZQ0910,Streptococcus anginosus 1 2 62CV,Streptococcus anginosus C1051,Streptococcus anginosus C238,Streptococcus anginosus CCUG 39159,Streptococcus anginosus F0211,Streptococcus anginosus SK1138,Streptococcus anginosus SK52,Streptococcus AS14,Streptococcus australis ATCC 700641,Streptococcus bovis ATCC 700338,Streptococcus BS35b,Streptococcus C150,Streptococcus C300,Streptococcus canis FSL Z3 227,Streptococcus constellatus pharyngis C1050,Streptococcus constellatus pharyngis C232,Streptococcus constellatus pharyngis C818,Streptococcus constellatus pharyngis SK1060,Streptococcus constellatus SK53,Streptococcus criceti,Streptococcus cristatus ATCC 51100,Streptococcus downei F0415,Streptococcus dysgalactiae ATCC 27957,Streptococcus dysgalactiae equisimilis 167,Streptococcus dysgalactiae equisimilis AC 2713,Streptococcus dysgalactiae equisimilis ATCC 12394,Streptococcus dysgalactiae equisimilis GGS 124,Streptococcus dysgalactiae equisimilis RE378,Streptococcus dysgalactiae equisimilis SK1249,Streptococcus dysgalactiae equisimilis SK1250,Streptococcus equi 4047,Streptococcus equi zooepidemicus,Streptococcus equi zooepidemicus ATCC 35246,Streptococcus equi zooepidemicus MGCS10565,Streptococcus equinus ATCC 9812,Streptococcus F0441,Streptococcus F0442,Streptococcus gallolyticus ATCC 43143,Streptococcus gallolyticus ATCC BAA 2069,Streptococcus gallolyticus TX20005,Streptococcus gallolyticus UCN34,Streptococcus GMD1S,Streptococcus GMD2S,Streptococcus GMD4S,Streptococcus GMD6S,Streptococcus gordonii Challis substr CH1,Streptococcus I G2,Streptococcus I P16,Streptococcus ictaluri 707 05,Streptococcus infantarius ATCC BAA 102,Streptococcus infantarius CJ18,Streptococcus infantis ATCC 700779,Streptococcus infantis SK1076,Streptococcus infantis SK1302,Streptococcus infantis SK970,Streptococcus infantis X,Streptococcus iniae 9117,Streptococcus iniae SF1,Streptococcus intermedius B196,Streptococcus intermedius C270,Streptococcus intermedius F0395,Streptococcus intermedius F0413,Streptococcus intermedius JTH08,Streptococcus intermedius SK54,Streptococcus lutetiensis 033,Streptococcus M143,Streptococcus M334,Streptococcus macacae NCTC 11558,Streptococcus macedonicus ACA DC 198,Streptococcus mitis ATCC 6249,Streptococcus mitis B6,Streptococcus mitis bv 2 F0392,Streptococcus mitis bv 2 SK95,Streptococcus mitis NCTC 12261,Streptococcus mitis SK1073,Streptococcus mitis SK1080,Streptococcus mitis SK321,Streptococcus mitis SK564,Streptococcus mitis SK569,Streptococcus mitis SK575,Streptococcus mitis SK579,Streptococcus mitis SK597,Streptococcus mitis SK616,Streptococcus mitis SPAR10,Streptococcus mutans 11A1,Streptococcus mutans 11SSST2,Streptococcus mutans 11VS1,Streptococcus mutans 14D,Streptococcus mutans 15JP3,Streptococcus mutans 15VF2,Streptococcus mutans 1ID3,Streptococcus mutans 1SM1,Streptococcus mutans 21,Streptococcus mutans 24,Streptococcus mutans 2ST1,Streptococcus mutans 2VS1,Streptococcus mutans 3SN1,Streptococcus mutans 4SM1,Streptococcus mutans 4VF1,Streptococcus mutans 5DC8,Streptococcus mutans 5SM3,Streptococcus mutans 66 2A,Streptococcus mutans 8ID3,Streptococcus mutans A19,Streptococcus mutans A9,Streptococcus mutans AC4446,Streptococcus mutans ATCC 25175,Streptococcus mutans B,Streptococcus mutans G123,Streptococcus mutans GS 5,Streptococcus mutans KK21,Streptococcus mutans KK23,Streptococcus mutans LJ23,Streptococcus mutans M21,Streptococcus mutans M230,Streptococcus mutans M2A,Streptococcus mutans N29,Streptococcus mutans N3209,Streptococcus mutans N34,Streptococcus mutans N66,Streptococcus mutans NCTC 11060,Streptococcus mutans NFSM1,Streptococcus mutans NFSM2,Streptococcus mutans NLML1,Streptococcus mutans NLML4,Streptococcus mutans NLML5,Streptococcus mutans NLML8,Streptococcus mutans NLML9,Streptococcus mutans NMT4863,Streptococcus mutans NN2025,Streptococcus mutans NV1996,Streptococcus mutans NVAB,Streptococcus mutans OMZ175,Streptococcus mutans R221,Streptococcus mutans S1B,Streptococcus mutans SA38,Streptococcus mutans SA41,Streptococcus mutans SF1,Streptococcus mutans SF12,Streptococcus mutans SF14,Streptococcus mutans SM1,Streptococcus mutans SM4,Streptococcus mutans SM6,Streptococcus mutans ST1,Streptococcus mutans ST6,Streptococcus mutans T4,Streptococcus mutans U138,Streptococcus mutans U2A,Streptococcus mutans U2B,Streptococcus mutans UA159,Streptococcus mutans W6,Streptococcus oligofermentans AS 1 3089,Streptococcus oral taxon 056 F0418,Streptococcus oral taxon 058 F0407,Streptococcus oral taxon 071 73H25AP,Streptococcus oralis,Streptococcus oralis ATCC 35037,Streptococcus oralis SK10,Streptococcus oralis SK100,Streptococcus oralis SK1074,Streptococcus oralis SK255,Streptococcus oralis SK304,Streptococcus oralis SK313,Streptococcus oralis SK610,Streptococcus oralis Uo5,Streptococcus parasanguinis ATCC 15912,Streptococcus parasanguinis ATCC 903,Streptococcus parasanguinis F0405,Streptococcus parasanguinis F0449,Streptococcus parasanguinis FW213,Streptococcus parasanguinis SK236,Streptococcus parauberis KCTC 11537,Streptococcus parauberis KRS 02083,Streptococcus parauberis KRS 02109,Streptococcus parauberis NCFD 2020,Streptococcus pasteurianus ATCC 43144,Streptococcus peroris ATCC 700780,Streptococcus pneumoniae 07AR0125,Streptococcus pneumoniae 1974M1 LZD,Streptococcus pneumoniae 2061376,Streptococcus pneumoniae 2061617,Streptococcus pneumoniae 2070005,Streptococcus pneumoniae 2070035,Streptococcus pneumoniae 2070108,Streptococcus pneumoniae 2070109,Streptococcus pneumoniae 2070335,Streptococcus pneumoniae 2070425,Streptococcus pneumoniae 2070531,Streptococcus pneumoniae 2070768,Streptococcus pneumoniae 2071004,Streptococcus pneumoniae 2071247,Streptococcus pneumoniae 2072047,Streptococcus pneumoniae 2080076,Streptococcus pneumoniae 2080913,Streptococcus pneumoniae 2081074,Streptococcus pneumoniae 2081685,Streptococcus pneumoniae 2082170,Streptococcus pneumoniae 2082239,Streptococcus pneumoniae 2090008,Streptococcus pneumoniae 3063 00,Streptococcus pneumoniae 4027 06,Streptococcus pneumoniae 4075 00,Streptococcus pneumoniae 459 5,Streptococcus pneumoniae 5185 06,Streptococcus pneumoniae 5652 06,Streptococcus pneumoniae 5787 06,Streptococcus pneumoniae 670 6B,Streptococcus pneumoniae 6735 05,Streptococcus pneumoniae 6901 05,Streptococcus pneumoniae 6963 05,Streptococcus pneumoniae 70585,Streptococcus pneumoniae 7286 06,Streptococcus pneumoniae 7533 05,Streptococcus pneumoniae 7879 04,Streptococcus pneumoniae 8190 05,Streptococcus pneumoniae A026,Streptococcus pneumoniae AP200,Streptococcus pneumoniae ATCC 700669,Streptococcus pneumoniae BR1064,Streptococcus pneumoniae BS397,Streptococcus pneumoniae BS455,Streptococcus pneumoniae BS457,Streptococcus pneumoniae BS458,Streptococcus pneumoniae Canada MDR 19A,Streptococcus pneumoniae Canada MDR 19F,Streptococcus pneumoniae CCRI 1974,Streptococcus pneumoniae CCRI 1974M2,Streptococcus pneumoniae CDC0288 04,Streptococcus pneumoniae CDC1087 00,Streptococcus pneumoniae CDC1873 00,Streptococcus pneumoniae CDC3059 06,Streptococcus pneumoniae CGSP14,Streptococcus pneumoniae D39,Streptococcus pneumoniae England14 9,Streptococcus pneumoniae EU NP01,Streptococcus pneumoniae EU NP02,Streptococcus pneumoniae EU NP03,Streptococcus pneumoniae EU NP04,Streptococcus pneumoniae EU NP05,Streptococcus pneumoniae G54,Streptococcus pneumoniae GA02254,Streptococcus pneumoniae GA02270,Streptococcus pneumoniae GA02506,Streptococcus pneumoniae GA02714,Streptococcus pneumoniae GA04175,Streptococcus pneumoniae GA04216,Streptococcus pneumoniae GA04375,Streptococcus pneumoniae GA04672,Streptococcus pneumoniae GA05245,Streptococcus pneumoniae GA05248,Streptococcus pneumoniae GA05578,Streptococcus pneumoniae GA06083,Streptococcus pneumoniae GA07228,Streptococcus pneumoniae GA07643,Streptococcus pneumoniae GA07914,Streptococcus pneumoniae GA08780,Streptococcus pneumoniae GA08825,Streptococcus pneumoniae GA11184,Streptococcus pneumoniae GA11304,Streptococcus pneumoniae GA11426,Streptococcus pneumoniae GA11663,Streptococcus pneumoniae GA11856,Streptococcus pneumoniae GA13224,Streptococcus pneumoniae GA13338,Streptococcus pneumoniae GA13430,Streptococcus pneumoniae GA13455,Streptococcus pneumoniae GA13494,Streptococcus pneumoniae GA13499,Streptococcus pneumoniae GA13637,Streptococcus pneumoniae GA13723,Streptococcus pneumoniae GA13856,Streptococcus pneumoniae GA14373,Streptococcus pneumoniae GA14688,Streptococcus pneumoniae GA14798,Streptococcus pneumoniae GA16121,Streptococcus pneumoniae GA16242,Streptococcus pneumoniae GA16531,Streptococcus pneumoniae GA16833,Streptococcus pneumoniae GA17227,Streptococcus pneumoniae GA17301,Streptococcus pneumoniae GA17328,Streptococcus pneumoniae GA17371,Streptococcus pneumoniae GA17457,Streptococcus pneumoniae GA17484,Streptococcus pneumoniae GA17545,Streptococcus pneumoniae GA17570,Streptococcus pneumoniae GA17719,Streptococcus pneumoniae GA17971,Streptococcus pneumoniae GA18068,Streptococcus pneumoniae GA18523,Streptococcus pneumoniae GA19077,Streptococcus pneumoniae GA19101,Streptococcus pneumoniae GA19451,Streptococcus pneumoniae GA19690,Streptococcus pneumoniae GA19923,Streptococcus pneumoniae GA19998,Streptococcus pneumoniae GA40028,Streptococcus pneumoniae GA40183,Streptococcus pneumoniae GA40410,Streptococcus pneumoniae GA40563,Streptococcus pneumoniae GA41277,Streptococcus pneumoniae GA41301,Streptococcus pneumoniae GA41317,Streptococcus pneumoniae GA41410,Streptococcus pneumoniae GA41437,Streptococcus pneumoniae GA41538,Streptococcus pneumoniae GA41565,Streptococcus pneumoniae GA41688,Streptococcus pneumoniae GA43257,Streptococcus pneumoniae GA43264,Streptococcus pneumoniae GA43265,Streptococcus pneumoniae GA43380,Streptococcus pneumoniae GA44128,Streptococcus pneumoniae GA44194,Streptococcus pneumoniae GA44288,Streptococcus pneumoniae GA44378,Streptococcus pneumoniae GA44386,Streptococcus pneumoniae GA44452,Streptococcus pneumoniae GA44500,Streptococcus pneumoniae GA44511,Streptococcus pneumoniae GA47033,Streptococcus pneumoniae GA47179,Streptococcus pneumoniae GA47210,Streptococcus pneumoniae GA47281,Streptococcus pneumoniae GA47283,Streptococcus pneumoniae GA47360,Streptococcus pneumoniae GA47368,Streptococcus pneumoniae GA47373,Streptococcus pneumoniae GA47388,Streptococcus pneumoniae GA47439,Streptococcus pneumoniae GA47461,Streptococcus pneumoniae GA47502,Streptococcus pneumoniae GA47522,Streptococcus pneumoniae GA47562,Streptococcus pneumoniae GA47597,Streptococcus pneumoniae GA47628,Streptococcus pneumoniae GA47688,Streptococcus pneumoniae GA47751,Streptococcus pneumoniae GA47760,Streptococcus pneumoniae GA47778,Streptococcus pneumoniae GA47794,Streptococcus pneumoniae GA47901,Streptococcus pneumoniae GA47976,Streptococcus pneumoniae GA49138,Streptococcus pneumoniae GA49194,Streptococcus pneumoniae GA49447,Streptococcus pneumoniae GA49542,Streptococcus pneumoniae GA52306,Streptococcus pneumoniae GA52612,Streptococcus pneumoniae GA54354,Streptococcus pneumoniae GA54644,Streptococcus pneumoniae GA56113,Streptococcus pneumoniae GA56348,Streptococcus pneumoniae GA58581,Streptococcus pneumoniae GA58771,Streptococcus pneumoniae GA58981,Streptococcus pneumoniae GA60080,Streptococcus pneumoniae GA60132,Streptococcus pneumoniae GA60190,Streptococcus pneumoniae GA62331,Streptococcus pneumoniae GA62681,Streptococcus pneumoniae gamPNI0373,Streptococcus pneumoniae Hungary19A 6,Streptococcus pneumoniae INV104,Streptococcus pneumoniae INV200,Streptococcus pneumoniae JJA,Streptococcus pneumoniae MLV 016,Streptococcus pneumoniae ND6012,Streptococcus pneumoniae Netherlands15B 37,Streptococcus pneumoniae NorthCarolina6A 23,Streptococcus pneumoniae NP070,Streptococcus pneumoniae NP112,Streptococcus pneumoniae NP127,Streptococcus pneumoniae NP141,Streptococcus pneumoniae NP170,Streptococcus pneumoniae OXC141,Streptococcus pneumoniae P1031,Streptococcus pneumoniae PCS125219,Streptococcus pneumoniae PCS70012,Streptococcus pneumoniae PCS8106,Streptococcus pneumoniae PCS81218,Streptococcus pneumoniae PCS8203,Streptococcus pneumoniae PCS8235,Streptococcus pneumoniae PNI0002,Streptococcus pneumoniae PNI0006,Streptococcus pneumoniae PNI0007,Streptococcus pneumoniae PNI0008,Streptococcus pneumoniae PNI0009,Streptococcus pneumoniae PNI0010,Streptococcus pneumoniae PNI0076,Streptococcus pneumoniae PNI0153,Streptococcus pneumoniae PNI0199,Streptococcus pneumoniae PNI0360,Streptococcus pneumoniae PNI0427,Streptococcus pneumoniae PNI0446,Streptococcus pneumoniae PT8114,Streptococcus pneumoniae R6,Streptococcus pneumoniae R6M2 PG,Streptococcus pneumoniae SP11 BS70,Streptococcus pneumoniae SP14 BS292,Streptococcus pneumoniae SP14 BS69,Streptococcus pneumoniae SP18 BS74,Streptococcus pneumoniae SP195,Streptococcus pneumoniae SP19 BS75,Streptococcus pneumoniae SP23 BS72,Streptococcus pneumoniae SP3 BS71,Streptococcus pneumoniae SP6 BS73,Streptococcus pneumoniae SP9 BS68,Streptococcus pneumoniae SP9v BS293,Streptococcus pneumoniae SPAR27,Streptococcus pneumoniae SPAR48,Streptococcus pneumoniae SPAR55,Streptococcus pneumoniae SPAR95,Streptococcus pneumoniae SPN021198,Streptococcus pneumoniae SPN034156,Streptococcus pneumoniae SPN034183,Streptococcus pneumoniae SPN061370,Streptococcus pneumoniae SPN072838,Streptococcus pneumoniae SPN1041,Streptococcus pneumoniae SPN7465,Streptococcus pneumoniae SPN994038,Streptococcus pneumoniae SPN994039,Streptococcus pneumoniae SPNA45,Streptococcus pneumoniae ST556,Streptococcus pneumoniae SV35,Streptococcus pneumoniae SV36,Streptococcus pneumoniae Taiwan19F 14,Streptococcus pneumoniae TCH8431 19A,Streptococcus pneumoniae TIGR4,Streptococcus porcinus Jelinkova 176,Streptococcus pseudopneumoniae ATCC BAA 960,Streptococcus pseudopneumoniae IS7493,Streptococcus pseudopneumoniae SK674,Streptococcus pseudoporcinus LQ 940 04,Streptococcus pseudoporcinus SPIN 20026,Streptococcus pyogenes A20,Streptococcus pyogenes Alab49,Streptococcus pyogenes ATCC 10782,Streptococcus pyogenes HKU QMH11M0907901,Streptococcus pyogenes HSC5,Streptococcus pyogenes M1 476,Streptococcus pyogenes M1 GAS,Streptococcus pyogenes M49 591,Streptococcus pyogenes Manfredo,Streptococcus pyogenes MGAS10270,Streptococcus pyogenes MGAS10394,Streptococcus pyogenes MGAS10750,Streptococcus pyogenes MGAS15252,Streptococcus pyogenes MGAS1882,Streptococcus pyogenes MGAS2096,Streptococcus pyogenes MGAS315,Streptococcus pyogenes MGAS5005,Streptococcus pyogenes MGAS6180,Streptococcus pyogenes MGAS8232,Streptococcus pyogenes MGAS9429,Streptococcus pyogenes NS88 2,Streptococcus pyogenes NZ131,Streptococcus pyogenes SSI 1,Streptococcus ratti FA 1,Streptococcus ratti FA 1 DSM 20564,Streptococcus salivarius 57 I,Streptococcus salivarius CCHSS3,Streptococcus salivarius JIM8777,Streptococcus salivarius K12,Streptococcus salivarius M18,Streptococcus salivarius PS4,Streptococcus salivarius SK126,Streptococcus sanguinis ATCC 29667,Streptococcus sanguinis ATCC 49296,Streptococcus sanguinis SK1,Streptococcus sanguinis SK1056,Streptococcus sanguinis SK1057,Streptococcus sanguinis SK1058,Streptococcus sanguinis SK1059,Streptococcus sanguinis SK1087,Streptococcus sanguinis SK115,Streptococcus sanguinis SK150,Streptococcus sanguinis SK160,Streptococcus sanguinis SK330,Streptococcus sanguinis SK340,Streptococcus sanguinis SK353,Streptococcus sanguinis SK355,Streptococcus sanguinis SK36,Streptococcus sanguinis SK405,Streptococcus sanguinis SK408,Streptococcus sanguinis SK49,Streptococcus sanguinis SK678,Streptococcus sanguinis SK72,Streptococcus sanguinis VMC66,Streptococcus SK140,Streptococcus SK643,Streptococcus sobrinus DSM 20742,Streptococcus suis 05HAH33,Streptococcus suis 05ZYH33,Streptococcus suis 89 1591,Streptococcus suis 98HAH33,Streptococcus suis A7,Streptococcus suis BM407,Streptococcus suis D12,Streptococcus suis D9,Streptococcus suis GZ1,Streptococcus suis JS14,Streptococcus suis P1 7,Streptococcus suis R61,Streptococcus suis S735,Streptococcus suis SC070731,Streptococcus suis SC84,Streptococcus suis SS12,Streptococcus suis ST1,Streptococcus suis ST3,Streptococcus suis T15,Streptococcus suis TL13,Streptococcus thermophilus CNCM I 1630,Streptococcus thermophilus CNRZ1066,Streptococcus thermophilus JIM 8232,Streptococcus thermophilus LMD 9,Streptococcus thermophilus LMG 18311,Streptococcus thermophilus MN ZLW 002,Streptococcus thermophilus MTCC 5460,Streptococcus thermophilus MTCC 5461,Streptococcus thermophilus ND03,Streptococcus tigurinus 1366,Streptococcus tigurinus AZ 3a,Streptococcus uberis 0140J,Streptococcus urinalis 2285 97,Streptococcus urinalis FB127 CNA 2,Streptococcus vestibularis ATCC 49124,Streptococcus vestibularis F0396,Sulfobacillus acidophilus DSM 10332,Sulfobacillus acidophilus TPY,Symbiobacterium thermophilum IAM 14863,Syntrophobotulus glycolicus DSM 8271,Syntrophomonas wolfei Goettingen,Syntrophothermus lipocalidus DSM 12680,Tepidanaerobacter acetatoxydans Re1,Tepidanaerobacter Re1,Tetragenococcus halophilus,Thermacetogenium phaeum DSM 12270,Thermaerobacter marianensis DSM 12885,Thermaerobacter subterraneus DSM 13965,Thermincola potens JR,Thermoanaerobacter brockii finnii Ako 1,Thermoanaerobacter ethanolicus CCSD1,Thermoanaerobacter ethanolicus JW 200,Thermoanaerobacter italicus Ab9,Thermoanaerobacter mathranii A3,Thermoanaerobacter pseudethanolicus ATCC 33223,Thermoanaerobacter siderophilus SR4,Thermoanaerobacter tengcongensis MB4,Thermoanaerobacter wiegelii Rt8 B1,Thermoanaerobacter X513,Thermoanaerobacter X514,Thermoanaerobacter X561,Thermoanaerobacterium saccharolyticum JW SL YS485,Thermoanaerobacterium thermosaccharolyticum DSM 571,Thermoanaerobacterium thermosaccharolyticum M0795,Thermoanaerobacterium xylanolyticum LX 11,Thermobacillus composti KWC4,Thermodesulfobium narugense DSM 14796,Thermosediminibacter oceani DSM 16646,Veillonella 3 1 44,Veillonella 6 1 27,Veillonella ACP1,Veillonella atypica ACS 049 V Sch6,Veillonella atypica ACS 134 V Col7a,Veillonella atypica KON,Veillonella dispar ATCC 17748,Veillonella oral taxon 158 F0412,Veillonella oral taxon 780 F0422,Veillonella parvula ACS 068 V Sch12,Veillonella parvula ATCC 17745,Veillonella parvula DSM 2008,Veillonella ratti ACS 216 V Col6b,Weissella ceti NC36,Weissella cibaria KACC 11862,Weissella confusa LBAE C39 2,Weissella koreensis KACC 15510,Weissella koreensis KCTC 3621,Weissella paramesenteroides ATCC 33313,Bhargavaea cecembensis DSE10,Blautia hansenii DSM 20583,Blautia hydrogenotrophica DSM 10507,Bulleidia extructa W1219,Caldalkalibacillus thermarum TA2 A1,Caloramator australicus RC3,Catenibacterium mitsuokai DSM 15897,Centipeda periodontii DSM 2778,Coprobacillus 29 1,Coprobacillus 3 3 56FAA,Coprobacillus 8 2 54BFAA,Coprobacillus D7,Desmospora 8437,Dethiobacter alkaliphilus AHT 1,Dialister invisus DSM 15470,Dialister micraerophilus DSM 19965,Dialister microaerophilus UPII 345 E,Dialister succinatiphilus YIT 11850,Dolosigranulum pigrum ATCC 51524,Dorea formicigenerans 4 6 53AFAA,Dorea formicigenerans ATCC 27755,Dorea longicatena DSM 13814,Epulopiscium N t morphotype B,Eremococcus coleocola ACS 139 V Col8,Erysipelotrichaceae bacterium 21 3,Erysipelotrichaceae bacterium 2 2 44A,Erysipelotrichaceae bacterium 3 1 53,Erysipelotrichaceae bacterium 5 2 54FAA,Erysipelotrichaceae bacterium 6 1 45,Eubacteriaceae bacterium ACC19a,Eubacteriaceae bacterium CM2,Eubacteriaceae bacterium CM5,Eubacteriaceae bacterium OBRC8,Facklamia hominis CCUG 36813,Facklamia ignava CCUG 37419,Facklamia languida CCUG 37842,Flavonifractor plautii ATCC 29863,Fructobacillus fructosus KCTC 3544,Gemella haemolysans ATCC 10379,Gemella haemolysans M341,Gemella moribillum M424,Gemella sanguinis M325,Granulicatella adiacens ATCC 49175,Granulicatella elegans ATCC 700633,Helcococcus kunzii ATCC 51366,Holdemania filiformis DSM 12042,Johnsonella ignava ATCC 51276,Kurthia JC30,Kurthia JC8E,Lachnospiraceae bacterium 1 1 57FAA,Lachnospiraceae bacterium 1 4 56FAA,Lachnospiraceae bacterium 2 1 46FAA,Lachnospiraceae bacterium 2 1 58FAA,Lachnospiraceae bacterium 3 1 46FAA,Lachnospiraceae bacterium 3 1 57FAA CT1,Lachnospiraceae bacterium 4 1 37FAA,Lachnospiraceae bacterium 5 1 57FAA,Lachnospiraceae bacterium 5 1 63FAA,Lachnospiraceae bacterium 6 1 63FAA,Lachnospiraceae bacterium 7 1 58FAA,Lachnospiraceae bacterium 8 1 57FAA,Lachnospiraceae bacterium 9 1 43BFAA,Lachnospiraceae bacterium ACC2,Lachnospiraceae bacterium ICM7,Lachnospiraceae bacterium oral taxon 082 F0431,Lachnospiraceae oral taxon 107 F0167,Lentibacillus Grbi,Listeriaceae bacterium TTU M1 001,Mitsuokella multacida DSM 20544,Mogibacterium CM50,Ornithinibacillus TW25,Parvimonas micra ATCC 33270,Parvimonas oral taxon 110 F0139,Parvimonas oral taxon 393 F0440,Pelosinus fermentans A11,Pelosinus fermentans A12,Pelosinus fermentans B3,Pelosinus fermentans B4,Pelosinus fermentans DSM 17108,Pelosinus fermentans JBW45,Peptoniphilus duerdenii ATCC BAA 1640,Peptoniphilus harei ACS 146 V Sch2b,Peptoniphilus indolicus ATCC 29427,Peptoniphilus lacrimalis 315 B,Peptoniphilus oral taxon 375 F0436,Peptoniphilus oral taxon 386 F0131,Peptoniphilus oral taxon 836 F0141,Peptoniphilus rhinitidis 1 13,Phascolarctobacterium YIT 12067,Planococcus antarcticus DSM 14505,Planococcus donghaensis MPA1U2,Planococcus halocryophilus Or1,Pseudoramibacter alactolyticus ATCC 23263,Ruminococcaceae bacterium D16,Salimicrobium MJ3,Shuttleworthia satelles DSM 14600,Solobacterium moorei F0204,Sporolactobacillus inulinus CASD,Sporolactobacillus vineae DSM 21990 SL153,Sporosarcina newyorkensis,Subdoligranulum 4 3 54A2FAA,Subdoligranulum variabile DSM 15176,Thermosinus carboxydivorans Nor1,Turicibacter HGF1,Turicibacter PC909,Bryantella formatexigens DSM 14469,Carboxydibrachium pacificum DSM 12653,Peptostreptococcus anaerobius 653 L,Peptostreptococcus anaerobius VPI 4330,Peptostreptococcus stomatis DSM 17678 |
| Fusobacteria | Fusobacterium 11 3 2,Fusobacterium 12 1B,Fusobacterium 1 1 41FAA,Fusobacterium 21 1A,Fusobacterium 2 1 31,Fusobacterium 3 1 27,Fusobacterium 3 1 33,Fusobacterium 3 1 36A2,Fusobacterium 3 1 5R,Fusobacterium 4 1 13,Fusobacterium 4 8,Fusobacterium 7 1,Fusobacterium D11,Fusobacterium D12,Fusobacterium gonidiaformans ATCC 25563,Fusobacterium mortiferum ATCC 9817,Fusobacterium necrophorum funduliforme 1 1 36S,Fusobacterium necrophorum funduliforme ATCC 51357,Fusobacterium necrophorum funduliforme Fnf 1007,Fusobacterium nucleatum animalis ATCC 51191,Fusobacterium nucleatum animalis F0419,Fusobacterium nucleatum ATCC 23726,Fusobacterium nucleatum ATCC 25586,Fusobacterium nucleatum ChDC F128,Fusobacterium nucleatum fusiforme ATCC 51190,Fusobacterium nucleatum polymorphum F0401,Fusobacterium nucleatum vincentii ATCC 49256,Fusobacterium oral taxon 370 F0437,Fusobacterium periodonticum ATCC 33693,Fusobacterium periodonticum D10,Fusobacterium ulcerans ATCC 49185,Fusobacterium varium ATCC 27725,Ilyobacter polytropus DSM 2926,Leptotrichia buccalis C 1013 b,Leptotrichia goodfellowii F0264,Leptotrichia hofstadii F0254,Sebaldella termitidis ATCC 33386,Streptobacillus moniliformis DSM 12112 |
| Gemmatimonadetes | Gemmatimonas aurantiaca T 27 |
| Nitrospinae | Nitrospina gracilis 3 211 |
| Nitrospirae | Candidatus Nitrospira defluvii,Leptospirillum ferriphilum ML 04,Leptospirillum ferrooxidans C2 3,Thermodesulfovibrio yellowstonii DSM 11347 |
| Planctomycetes | Isosphaera pallida ATCC 43644,Phycisphaera mikurensis NBRC 102666,Pirellula staleyi DSM 6068,Planctomyces brasiliensis DSM 5305,Planctomyces limnophilus DSM 3776,Planctomyces maris DSM 8797,Rhodopirellula baltica SH28,Rhodopirellula baltica SH 1,Rhodopirellula baltica SWK14,Rhodopirellula baltica WH47,Rhodopirellula europaea 6C,Rhodopirellula europaea SH398,Rhodopirellula maiorica SM1,Rhodopirellula sallentina SM41,Rhodopirellula SWK7,Singulisphaera acidiphila DSM 18658,Blastopirellula marina DSM 3645,Gemmata obscuriglobus UQM 2246,Schlesneria paludicola DSM 18645,planctomycete KSU 1 |
| Alphaproteobacteria | Acetobacter pasteurianus 386B,Acetobacter pasteurianus IFO 3283 01,Acetobacter pasteurianus IFO 3283 01 42C,Acetobacter pasteurianus IFO 3283 03,Acetobacter pasteurianus IFO 3283 07,Acetobacter pasteurianus IFO 3283 12,Acetobacter pasteurianus IFO 3283 12 uid158379,Acetobacter pasteurianus IFO 3283 22,Acetobacter pasteurianus IFO 3283 26,Acetobacter pasteurianus IFO 3283 32,Acidiphilium cryptum JF 5,Acidiphilium multivorum AIU301,Agrobacterium fabrum C58,Agrobacterium H13 3,Agrobacterium radiobacter K84,Agrobacterium vitis S4,Agromonas oligotrophica S58,Anaplasma centrale Israel,Anaplasma marginale Dawn,Anaplasma marginale Florida,Anaplasma marginale Gypsy Plains,Anaplasma marginale Maries,Anaplasma phagocytophilum Dog2,Anaplasma phagocytophilum HZ,Anaplasma phagocytophilum HZ2,Anaplasma phagocytophilum JM,Asticcacaulis excentricus CB 48,Azorhizobium caulinodans ORS 571,Azospirillum B510,Azospirillum brasilense Sp245,Azospirillum lipoferum 4B,Bartonella alsatica IBS 382,Bartonella australis Aust NH1,Bartonella bacilliformis INS,Bartonella bacilliformis KC583,Bartonella birtlesii LL WM9,Bartonella clarridgeiae 73,Bartonella DB5 6,Bartonella doshiae NCTC 12862,Bartonella elizabethae F9251,Bartonella elizabethae Re6043vi,Bartonella grahamii as4aup,Bartonella henselae Houston 1,Bartonella melophagi K 2C,Bartonella quintana RM 11,Bartonella quintana Toulouse,Bartonella rattimassiliensis 15908,Bartonella tamiae Th239,Bartonella tamiae Th307,Bartonella taylorii 8TBB,Bartonella tribocorum CIP 105476,Bartonella vinsonii arupensis OK 94 513,Bartonella vinsonii arupensis Pm136co,Bartonella vinsonii berkhoffii Winnie,Bartonella washoensis 085 0475,Bartonella washoensis Sb944nv,Beijerinckia indica ATCC 9039,Bradyrhizobium,Bradyrhizobium BTAi1,Bradyrhizobium CCGE LA001,Bradyrhizobium japonicum USDA 110,Bradyrhizobium japonicum USDA 6,Bradyrhizobium ORS 278,Bradyrhizobium ORS 285,Bradyrhizobium ORS 375,Bradyrhizobium S23321,Bradyrhizobium STM 3843,Bradyrhizobium WSM1253,Bradyrhizobium WSM471,Bradyrhizobium YR681,Brevundimonas BAL3,Brevundimonas diminuta 470 4,Brevundimonas diminuta ATCC 11568,Brevundimonas subvibrioides ATCC 15264,Brucella 83 13,Brucella abortus 2308 A,Brucella abortus A13334,Brucella abortus bv 1 9 941,Brucella abortus bv 1 NI010,Brucella abortus bv 1 NI016,Brucella abortus bv 1 NI021,Brucella abortus bv 1 NI259,Brucella abortus bv 1 NI435a,Brucella abortus bv 1 NI474,Brucella abortus bv 1 NI486,Brucella abortus bv 1 NI488,Brucella abortus bv 2 86 8 59,Brucella abortus bv 3 Tulya,Brucella abortus bv 4 292,Brucella abortus bv 5 B3196,Brucella abortus bv 6 870,Brucella abortus bv 9 C68,Brucella abortus NCTC 8038,Brucella abortus S19,Brucella BO1,Brucella BO2,Brucella canis ATCC 23365,Brucella canis HSK A52141,Brucella ceti B1 94,Brucella ceti Cudo,Brucella ceti M13 05 1,Brucella ceti M490 95 1,Brucella ceti M644 93 1,Brucella ceti TE10759 12,Brucella ceti TE28753 12,Brucella F5 99,Brucella melitensis ATCC 23457,Brucella melitensis biovar Abortus 2308,Brucella melitensis bv 1 16M,Brucella melitensis bv 1 Rev 1,Brucella melitensis bv 2 63 9,Brucella melitensis bv 3 Ether,Brucella melitensis M28,Brucella melitensis M5 90,Brucella melitensis NI,Brucella microti CCM 4915,Brucella neotomae 5K33,Brucella NF 2653,Brucella NVSL 07 0026,Brucella ovis ATCC 25840,Brucella pinnipedialis B2 94,Brucella pinnipedialis M163 99 10,Brucella pinnipedialis M292 94 1,Brucella suis 1330,Brucella suis ATCC 23445,Brucella suis bv 3 686,Brucella suis bv 4 40,Brucella suis bv 5 513,Brucella suis VBI22,Candidatus Hodgkinia cicadicola Dsem,Candidatus Liberibacter americanus PW SP,Candidatus Liberibacter americanus Sao Paulo,Candidatus Liberibacter asiaticus gxpsy,Candidatus Liberibacter asiaticus psy62,Candidatus Liberibacter solanacearum CLso ZC1,Candidatus Midichloria mitochondrii IricVA,Candidatus Pelagibacter HTCC7211,Candidatus Pelagibacter IMCC9063,Candidatus Pelagibacter ubique HTCC1002,Candidatus Pelagibacter ubique HTCC1062,Candidatus Puniceispirillum marinum IMCC1322,Candidatus Rickettsia amblyommii GAT 30V,Caulobacter AP07,Caulobacter crescentus CB15,Caulobacter crescentus NA1000,Caulobacter K31,Caulobacter segnis ATCC 21756,Dinoroseobacter shibae DFL 12,Ehrlichia canis Jake,Ehrlichia chaffeensis Arkansas,Ehrlichia chaffeensis Sapulpa,Ehrlichia muris AS145,Ehrlichia ruminantium Gardel,Ehrlichia ruminantium Welgevonden,Erythrobacter litoralis HTCC2594,Erythrobacter NAP1,Erythrobacter SD 21,Gluconacetobacter diazotrophicus PAl 5,Gluconacetobacter europaeus LMG 18494,Gluconacetobacter hansenii ATCC 23769,Gluconacetobacter oboediens 174Bp2,Gluconacetobacter SXCC 1,Gluconacetobacter xylinus NBRC 3288,Gluconobacter frateurii NBRC 101659,Gluconobacter morbifer G707,Gluconobacter oxydans 621H,Gluconobacter oxydans H24,Gluconobacter thailandicus NBRC 3255,Granulibacter bethesdensis CGDNIH1,Hirschia baltica ATCC 49814,Hyphomicrobium denitrificans 1NES1,Hyphomicrobium denitrificans ATCC 51888,Hyphomicrobium MC1,Hyphomicrobium nitrativorans NL23,Hyphomonas neptunium ATCC 15444,Jannaschia CCS1,Ketogulonicigenium vulgare WSH 001,Ketogulonicigenium vulgare Y25,Magnetospirillum gryphiswaldense MSR 1 v2,Magnetospirillum magneticum AMB 1,Magnetospirillum SO 1,Maricaulis maris MCS10,Mesorhizobium alhagi CCNWXJ12 2,Mesorhizobium amorphae CCNWGS0123,Mesorhizobium australicum WSM2073,Mesorhizobium ciceri biovar biserrulae WSM1271,Mesorhizobium loti MAFF303099,Mesorhizobium metallidurans STM 2683,Mesorhizobium opportunistum WSM2075,Methylobacterium 4 46,Methylobacterium chloromethanicum CM4,Methylobacterium extorquens AM1,Methylobacterium extorquens DM4,Methylobacterium extorquens DSM 13060,Methylobacterium extorquens PA1,Methylobacterium GXF4,Methylobacterium mesophilicum SR1 6 6,Methylobacterium nodulans ORS 2060,Methylobacterium populi BJ001,Methylobacterium radiotolerans JCM 2831,Methylocella silvestris BL2,Methylocystis SC2,Micavibrio aeruginosavorus ARL 13,Micavibrio EPB,Neorickettsia risticii Illinois,Neorickettsia sennetsu Miyayama,Nitrobacter hamburgensis X14,Nitrobacter Nb 311A,Nitrobacter winogradskyi Nb 255,Novosphingobium AP12,Novosphingobium aromaticivorans DSM 12444,Novosphingobium nitrogenifigens DSM 19370,Novosphingobium pentaromativorans US6 1,Novosphingobium PP1Y,Novosphingobium Rr 2 17,Ochrobactrum anthropi ATCC 49188,Ochrobactrum anthropi CTS 325,Ochrobactrum CDB2,Ochrobactrum intermedium LMG 3301,Ochrobactrum intermedium M86,Octadecabacter antarcticus 307,Octadecabacter arcticus 238,Oligotropha carboxidovorans OM4,Oligotropha carboxidovorans OM5,Orientia tsutsugamushi Boryong,Orientia tsutsugamushi Ikeda,Paracoccus aminophilus JCM 7686,Paracoccus denitrificans PD1222,Paracoccus TRP,Parvibaculum lavamentivorans DS 1,Parvularcula bermudensis HTCC2503,Pelagibacterium halotolerans B2,Phaeobacter gallaeciensis 2 10,Phaeobacter gallaeciensis DSM 17395,Phaeobacter gallaeciensis DSM 26640,Phenylobacterium zucineum HLK1,Pseudovibrio FO BEG1,Pseudovibrio JE062,Rhizobium AP16,Rhizobium CCGE 510,Rhizobium CF080,Rhizobium CF122,Rhizobium CF142,Rhizobium etli 8C 3,Rhizobium etli Brasil 5,Rhizobium etli bv mimosae Mim1,Rhizobium etli CFN 42,Rhizobium etli CIAT 652,Rhizobium etli CIAT 894,Rhizobium etli CNPAF512,Rhizobium etli GR56,Rhizobium etli IE4771,Rhizobium etli Kim 5,Rhizobium IRBG74,Rhizobium leguminosarum bv trifolii WSM1325,Rhizobium leguminosarum bv trifolii WSM2012,Rhizobium leguminosarum bv trifolii WSM2297,Rhizobium leguminosarum bv trifolii WSM2304,Rhizobium leguminosarum bv trifolii WSM597,Rhizobium leguminosarum bv trifolii WU95,Rhizobium leguminosarum bv viciae 3841,Rhizobium leguminosarum bv viciae USDA 2370,Rhizobium leguminosarum bv viciae WSM1455,Rhizobium lupini HPC L,Rhizobium mesoamericanum STM3625,Rhizobium NGR234,Rhizobium PDO1 076,Rhizobium Pop5,Rhizobium tropici CIAT 899,Rhodobacter capsulatus SB 1003,Rhodobacter sphaeroides 2 4 1,Rhodobacter sphaeroides ATCC 17025,Rhodobacter sphaeroides ATCC 17029,Rhodobacter sphaeroides KD131,Rhodobacter sphaeroides WS8N,Rhodobacter SW2,Rhodomicrobium vannielii ATCC 17100,Rhodopseudomonas palustris BisA53,Rhodopseudomonas palustris BisB18,Rhodopseudomonas palustris BisB5,Rhodopseudomonas palustris CGA009,Rhodopseudomonas palustris DX 1,Rhodopseudomonas palustris HaA2,Rhodopseudomonas palustris TIE 1,Rhodospirillum centenum SW,Rhodospirillum photometricum,Rhodospirillum rubrum ATCC 11170,Rhodospirillum rubrum F11,Rickettsia africae ESF 5,Rickettsia akari Hartford,Rickettsia australis Cutlack,Rickettsia bellii OSU 85 389,Rickettsia bellii RML369 C,Rickettsia canadensis CA410,Rickettsia canadensis McKiel,Rickettsia conorii Malish 7,Rickettsia endosymbiont of Ixodes scapularis,Rickettsia felis URRWXCal2,Rickettsia heilongjiangensis 054,Rickettsia helvetica C9P9,Rickettsia japonica YH,Rickettsia massiliae AZT80,Rickettsia massiliae MTU5,Rickettsia montanensis OSU 85 930,Rickettsia parkeri Portsmouth,Rickettsia peacockii Rustic,Rickettsia philipii 364D,Rickettsia prowazekii Breinl,Rickettsia prowazekii BuV67 CWPP,Rickettsia prowazekii Chernikova,Rickettsia prowazekii Dachau,Rickettsia prowazekii GvV257,Rickettsia prowazekii Katsinyian,Rickettsia prowazekii Madrid E,Rickettsia prowazekii NMRC Madrid E,Rickettsia prowazekii Rp22,Rickettsia prowazekii RpGvF24,Rickettsia rhipicephali 3 7 female6 CWPP,Rickettsia rickettsii Sheila Smith,Rickettsia rickettsii Arizona,Rickettsia rickettsii Brazil,Rickettsia rickettsii Colombia,Rickettsia rickettsii Hauke,Rickettsia rickettsii Hino,Rickettsia rickettsii Hlp 2,Rickettsia rickettsii Iowa,Rickettsia sibirica 246,Rickettsia slovaca 13 B,Rickettsia slovaca D CWPP,Rickettsia typhi B9991CWPP,Rickettsia typhi TH1527,Rickettsia typhi Wilmington,Roseobacter AzwK 3b,Roseobacter CCS2,Roseobacter denitrificans OCh 114,Roseobacter GAI101,Roseobacter litoralis Och 149,Roseobacter MED193,Roseobacter SK209 2 6,Ruegeria pomeroyi DSS 3,Ruegeria R11,Ruegeria TM1040,Ruegeria TW15,Sinorhizobium fredii HH103,Sinorhizobium fredii USDA 257,Sinorhizobium medicae WSM419,Sinorhizobium meliloti 1021,Sinorhizobium meliloti 2011,Sinorhizobium meliloti AK83,Sinorhizobium meliloti BL225C,Sinorhizobium meliloti CCNWSX0020,Sinorhizobium meliloti GR4,Sinorhizobium meliloti Rm41,Sinorhizobium meliloti SM11,Sphingobium AP49,Sphingobium chlorophenolicum L 1,Sphingobium indicum B90A,Sphingobium japonicum UT26S,Sphingobium SYK 6,Sphingobium yanoikuyae ATCC 51230,Sphingobium yanoikuyae XLDN2 5,Sphingomonas echinoides ATCC 14820,Sphingomonas elodea ATCC 31461,Sphingomonas KC8,Sphingomonas LH128,Sphingomonas MM 1,Sphingomonas PAMC 26605,Sphingomonas PAMC 26617,Sphingomonas PAMC 26621,Sphingomonas S17,Sphingomonas SKA58,Sphingomonas wittichii RW1,Sphingopyxis alaskensis RB2256,Starkeya novella DSM 506,Tistrella mobilis KA081020 065,Wolbachia endosymbiont of Culex quinquefasciatus JHB,Wolbachia endosymbiont of Culex quinquefasciatus Pel,Wolbachia endosymbiont of Drosophila ananassae,Wolbachia endosymbiont of Drosophila melanogaster,Wolbachia endosymbiont of Drosophila simulans,Wolbachia endosymbiont of Drosophila simulans wHa,Wolbachia endosymbiont of Drosophila simulans wNo,Wolbachia endosymbiont of Drosophila willistoni TSC 14030 0811 24,Wolbachia endosymbiont of Muscidifurax uniraptor,Wolbachia endosymbiont of Onchocerca ochengi,Wolbachia endosymbiont TRS of Brugia malayi,Wolbachia endosymbiont wVitB of Nasonia vitripennis,Wolbachia pipientis wAlbB,Wolbachia wRi,Xanthobacter autotrophicus Py2,Zymomonas mobilis ATCC 10988,Zymomonas mobilis ATCC 29191,Zymomonas mobilis CP4 NRRL B 14023,Zymomonas mobilis NCIMB 11163,Zymomonas mobilis pomaceae ATCC 29192,Zymomonas mobilis ZM4,Bradyrhizobiaceae bacterium SG 6C,Caenispirillum salinarum AK4,Celeribacter baekdonensis B30,Chelativorans BNC1,Citreicella 357,Citreicella SE45,Citromicrobium bathyomarinum JL354,Citromicrobium JLT1363,Commensalibacter intestini A911,Fulvimarina pelagi HTCC2506,Hoeflea phototrophica DFL 43,Labrenzia aggregata IAM 12614,Labrenzia alexandrii DFL 11,Leisingera methylohalidivorans DSM 14336,Loktanella vestfoldensis SKA53,Maritimibacter alkaliphilus HTCC2654,Methylosinus trichosporium OB3b,Microvirga WSM3557,Nitratireductor aquibiodomus RA22,Nitratireductor indicus C115,Nitratireductor pacificus pht 3B,Oceanibaculum indicum P24,Oceanibulbus indolifex HEL 45,Oceanicaulis alexandrii HTCC2633,Oceanicola batsensis HTCC2597,Oceanicola granulosus HTCC2516,Oceanicola S124,Oceaniovalibus guishaninsula JLT2003,Pelagibaca bermudensis HTCC2601,Phaeospirillum molischianum,Phyllobacterium YR531,Polymorphum gilvum SL003B 26A1,Rhodobacteraceae bacterium KLH11,Rhodobacterales bacterium HTCC2083,Rhodobacterales bacterium HTCC2150,Rhodobacterales bacterium HTCC2255,Rhodobacterales bacterium Y4I,Rhodovulum PH10,Roseibium TrichSKD4,Roseomonas cervicalis ATCC 49957,Roseovarius 217,Roseovarius nubinhibens ISM,Roseovarius TM1035,Sagittula stellata E 37,Sulfitobacter EE 36,Sulfitobacter NAS 14 1,Thalassobaculum L2,Thalassospira profundimaris WP0211,Thalassospira xiamenensis M 5 DSM 17429,SAR 116 cluster alpha proteobacterium HIMB100,Silicibacter lacuscaerulensis ITI 1157,Silicibacter TrichCH4B,Thalassiobium R2A62,Candidatus Odyssella thessalonicensis L13,Magnetococcus MC 1 |
| Betaproteobacteria | Achromobacter xylosoxidans,Achromobacter xylosoxidans A8,Achromobacter xylosoxidans NBRC 15126,Acidovorax avenae ATCC 19860,Acidovorax citrulli AAC00 1,Acidovorax ebreus TPSY,Acidovorax JS42,Acidovorax KKS102,Advenella kashmirensis WT001,Alicycliphilus denitrificans BC,Alicycliphilus denitrificans K601,Aromatoleum aromaticum EbN1,Azoarcus BH72,Azoarcus KH32C,Bordetella avium 197N,Bordetella bronchiseptica 1289,Bordetella bronchiseptica 253,Bordetella bronchiseptica Bbr77,Bordetella bronchiseptica D445,Bordetella bronchiseptica MO149,Bordetella bronchiseptica RB50,Bordetella holmesii F627,Bordetella holmesii H558,Bordetella parapertussis 12822,Bordetella parapertussis 18323,Bordetella parapertussis Bpp5,Bordetella pertussis CS,Bordetella pertussis Tohama I,Bordetella petrii,Burkholderia 383,Burkholderia ambifaria AMMD,Burkholderia ambifaria IOP40 10,Burkholderia ambifaria MC40 6,Burkholderia ambifaria MEX 5,Burkholderia BT03,Burkholderia CCGE1001,Burkholderia CCGE1002,Burkholderia CCGE1003,Burkholderia cenocepacia AU 1054,Burkholderia cenocepacia BC7,Burkholderia cenocepacia H111,Burkholderia cenocepacia HI2424,Burkholderia cenocepacia J2315,Burkholderia cenocepacia K56 2Valvano,Burkholderia cenocepacia MC0 3,Burkholderia cenocepacia PC184,Burkholderia cepacia GG4,Burkholderia Ch1 1,Burkholderia dolosa AUO158,Burkholderia gladioli BSR3,Burkholderia glumae BGR1,Burkholderia graminis C4D1M,Burkholderia H160,Burkholderia KJ006,Burkholderia mallei 2002721280,Burkholderia mallei ATCC 10399,Burkholderia mallei ATCC 23344,Burkholderia mallei FMH,Burkholderia mallei GB8 horse 4,Burkholderia mallei JHU,Burkholderia mallei NCTC 10229,Burkholderia mallei NCTC 10247,Burkholderia mallei PRL 20,Burkholderia mallei SAVP1,Burkholderia multivorans,Burkholderia multivorans ATCC 17616,Burkholderia multivorans ATCC BAA 247,Burkholderia multivorans CGD1,Burkholderia multivorans CGD2,Burkholderia multivorans CGD2M,Burkholderia oklahomensis C6786,Burkholderia oklahomensis EO147,Burkholderia phenoliruptrix BR3459a,Burkholderia phymatum STM815,Burkholderia phytofirmans PsJN,Burkholderia pseudomallei 1026a,Burkholderia pseudomallei 1026b,Burkholderia pseudomallei 1106a,Burkholderia pseudomallei 1106b,Burkholderia pseudomallei 112,Burkholderia pseudomallei 1258a,Burkholderia pseudomallei 1258b,Burkholderia pseudomallei 14,Burkholderia pseudomallei 1655,Burkholderia pseudomallei 1710a,Burkholderia pseudomallei 1710b,Burkholderia pseudomallei 305,Burkholderia pseudomallei 354a,Burkholderia pseudomallei 354e,Burkholderia pseudomallei 406e,Burkholderia pseudomallei 576,Burkholderia pseudomallei 668,Burkholderia pseudomallei 7894,Burkholderia pseudomallei 9,Burkholderia pseudomallei 91,Burkholderia pseudomallei B7210,Burkholderia pseudomallei BCC215,Burkholderia pseudomallei BPC006,Burkholderia pseudomallei DM98,Burkholderia pseudomallei K96243,Burkholderia pseudomallei MSHR1043,Burkholderia pseudomallei MSHR305,Burkholderia pseudomallei MSHR346,Burkholderia pseudomallei NCTC 13177,Burkholderia pseudomallei NCTC 13179,Burkholderia pseudomallei Pakistan 9,Burkholderia pseudomallei Pasteur 52237,Burkholderia pseudomallei S13,Burkholderia rhizoxinica HKI 454,Burkholderia RPE64,Burkholderia SJ98,Burkholderia terrae BS001,Burkholderia thailandensis Bt4,Burkholderia thailandensis E264,Burkholderia thailandensis MSMB121,Burkholderia thailandensis MSMB43,Burkholderia thailandensis TXDOH,Burkholderia TJI49,Burkholderia ubonensis Bu,Burkholderia vietnamiensis G4,Burkholderia xenovorans LB400,Burkholderia YI23,Candidatus Accumulibacter phosphatis clade IIA UW 1,Candidatus Burkholderia kirkii UZHbot1,Candidatus Kinetoplastibacterium blastocrithidii ex Strigomonas culicis,Candidatus Kinetoplastibacterium blastocrithidii TCC012E,Candidatus Kinetoplastibacterium crithidii ex Angomonas deanei ATCC 30255,Candidatus Kinetoplastibacterium crithidii TCC036E,Candidatus Kinetoplastibacterium desouzaii TCC079E,Candidatus Kinetoplastibacterium galatii TCC219,Candidatus Kinetoplastibacterium oncopeltii TCC290E,Candidatus Tremblaya phenacola PAVE,Candidatus Tremblaya princeps PCIT,Candidatus Tremblaya princeps PCVAL,Candidatus Zinderia insecticola CARI,Chromobacterium violaceum ATCC 12472,Collimonas fungivorans Ter331,Comamonas testosteroni ATCC 11996,Comamonas testosteroni CNB 2,Comamonas testosteroni KF 1,Comamonas testosteroni S44,Cupriavidus basilensis OR16,Cupriavidus HMR 1,Cupriavidus metallidurans CH34,Cupriavidus necator HPC L,Cupriavidus necator N 1,Cupriavidus taiwanensis LMG 19424,Dechloromonas aromatica RCB,Dechlorosoma suillum PS,Delftia acidovorans SPH 1,Delftia Cs1 4,Gallionella capsiferriformans ES 2,Herbaspirillum CF444,Herbaspirillum frisingense GSF30,Herbaspirillum GW103,Herbaspirillum seropedicae SmR1,Herbaspirillum YR522,Herminiimonas arsenicoxydans,Laribacter hongkongensis HLHK9,Leptothrix cholodnii SP 6,Leptothrix ochracea L12,Methylibium petroleiphilum PM1,Methylobacillus flagellatus KT,Methylotenera 301,Methylotenera mobilis JLW8,Methylovorus glucosetrophus SIP3 4,Methylovorus MP688,Neisseria bacilliformis ATCC BAA 1200,Neisseria cinerea ATCC 14685,Neisseria elongata glycolytica ATCC 29315,Neisseria flavescens NRL30031 H210,Neisseria flavescens SK114,Neisseria gonorrhoeae 1291,Neisseria gonorrhoeae 35 02,Neisseria gonorrhoeae DGI18,Neisseria gonorrhoeae DGI2,Neisseria gonorrhoeae F62,Neisseria gonorrhoeae FA19,Neisseria gonorrhoeae FA6140,Neisseria gonorrhoeae FA 1090,Neisseria gonorrhoeae MS11,Neisseria gonorrhoeae NCCP11945,Neisseria gonorrhoeae PID1,Neisseria gonorrhoeae PID18,Neisseria gonorrhoeae PID24 1,Neisseria gonorrhoeae PID332,Neisseria gonorrhoeae SK 92 679,Neisseria gonorrhoeae SK 93 1035,Neisseria gonorrhoeae TCDC NG08107,Neisseria GT4A CT1,Neisseria lactamica 020 06,Neisseria lactamica ATCC 23970,Neisseria lactamica Y92 1009,Neisseria macacae ATCC 33926,Neisseria meningitidis 053442,Neisseria meningitidis 12888,Neisseria meningitidis 2001212,Neisseria meningitidis 2002038,Neisseria meningitidis 2004090,Neisseria meningitidis 2006087,Neisseria meningitidis 2007056,Neisseria meningitidis 4119,Neisseria meningitidis 61103,Neisseria meningitidis 63006,Neisseria meningitidis 63041,Neisseria meningitidis 63049,Neisseria meningitidis 65014,Neisseria meningitidis 68094,Neisseria meningitidis 69096,Neisseria meningitidis 69166,Neisseria meningitidis 70012,Neisseria meningitidis 70030,Neisseria meningitidis 77221,Neisseria meningitidis 8013,Neisseria meningitidis 80179,Neisseria meningitidis 87255,Neisseria meningitidis 88050,Neisseria meningitidis 92045,Neisseria meningitidis 93003,Neisseria meningitidis 93004,Neisseria meningitidis 9506,Neisseria meningitidis 96023,Neisseria meningitidis 961 5945,Neisseria meningitidis 97014,Neisseria meningitidis 97020,Neisseria meningitidis 97021,Neisseria meningitidis 9757,Neisseria meningitidis 98008,Neisseria meningitidis 98080,Neisseria meningitidis alpha14,Neisseria meningitidis alpha704,Neisseria meningitidis alpha710,Neisseria meningitidis ATCC 13091,Neisseria meningitidis CU385,Neisseria meningitidis ES14902,Neisseria meningitidis FAM18,Neisseria meningitidis G2136,Neisseria meningitidis H44 76,Neisseria meningitidis M01 240013,Neisseria meningitidis M01 240149,Neisseria meningitidis M01 240355,Neisseria meningitidis M04 240196,Neisseria meningitidis M0579,Neisseria meningitidis M13255,Neisseria meningitidis M13399,Neisseria meningitidis M6190,Neisseria meningitidis M7089,Neisseria meningitidis M7124,Neisseria meningitidis MC58,Neisseria meningitidis N1568,Neisseria meningitidis NM126,Neisseria meningitidis NM140,Neisseria meningitidis NM174,Neisseria meningitidis NM183,Neisseria meningitidis NM220,Neisseria meningitidis NM233,Neisseria meningitidis NM255,Neisseria meningitidis NM2657,Neisseria meningitidis NM2781,Neisseria meningitidis NM2795,Neisseria meningitidis NM3001,Neisseria meningitidis NM3081,Neisseria meningitidis NM3642,Neisseria meningitidis NM3652,Neisseria meningitidis NM418,Neisseria meningitidis NM422,Neisseria meningitidis NM576,Neisseria meningitidis NM586,Neisseria meningitidis NM762,Neisseria meningitidis NZ 05 33,Neisseria meningitidis OX99 30304,Neisseria meningitidis WUE 2594,Neisseria meningitidis Z2491,Neisseria mucosa ATCC 25996,Neisseria mucosa C102,Neisseria oral taxon 014 F0314,Neisseria oral taxon 020 F0370,Neisseria polysaccharea ATCC 43768,Neisseria shayeganii 871,Neisseria sicca ATCC 29256,Neisseria sicca VK64,Neisseria subflava NJ9703,Neisseria wadsworthii 9715,Neisseria weaveri ATCC 51223,Neisseria weaveri LMG 5135,Nitrosomonas AL212,Nitrosomonas europaea ATCC 19718,Nitrosomonas eutropha C91,Nitrosomonas Is79A3,Nitrosospira multiformis ATCC 25196,Polaromonas CF318,Polaromonas JS666,Polaromonas naphthalenivorans CJ2,Polynucleobacter necessarius asymbioticus QLW P1DMWA 1,Polynucleobacter necessarius STIR1,Pseudogulbenkiania NH8B,Pusillimonas T7 7,Ralstonia 5 2 56FAA,Ralstonia 5 7 47FAA,Ralstonia eutropha H16,Ralstonia eutropha JMP134,Ralstonia PBA,Ralstonia pickettii 12D,Ralstonia pickettii 12J,Ralstonia solanacearum CFBP2957,Ralstonia solanacearum CMR15,Ralstonia solanacearum FQY 4 f,Ralstonia solanacearum GMI1000,Ralstonia solanacearum K60 1,Ralstonia solanacearum MolK2,Ralstonia solanacearum Po82,Ralstonia solanacearum PSI07,Ralstonia solanacearum UW551,Ramlibacter tataouinensis TTB310,Rhodoferax ferrireducens T118,Rubrivivax benzoatilyticus JA2,Rubrivivax gelatinosus IL144,Sideroxydans lithotrophicus ES 1,Taylorella asinigenitalis 14 45,Taylorella asinigenitalis MCE3,Taylorella equigenitalis 14 56,Taylorella equigenitalis ATCC 35865,Taylorella equigenitalis MCE9,Thauera MZ1T,Thiobacillus denitrificans ATCC 25259,Thiomonas 3As,Thiomonas intermedia K12,Variovorax CF313,Variovorax paradoxus B4,Variovorax paradoxus EPS,Variovorax paradoxus S110,Verminephrobacter At4,Verminephrobacter eiseniae EF01 2,Burkholderiales bacterium 1 1 47,Burkholderiales bacterium JOSHI 001,Comamonadaceae bacterium CR,Eikenella corrodens ATCC 23834,Hydrogenophaga PBC,Hylemonella gracilis ATCC 19624,Janthinobacterium HH01,Janthinobacterium Marseille,Janthinobacterium PAMC 25724,Kingella denitrificans ATCC 33394,Kingella kingae ATCC 23330,Kingella kingae PYKK081,Kingella oralis ATCC 51147,Lautropia mirabilis ATCC 51599,Limnobacter MED105,Massilia timonae CCUG 45783,Methylophilales bacterium HTCC2181,Methyloversatilis universalis FAM5,Oxalobacter formigenes HOxBLS,Oxalobacter formigenes OXCC13,Oxalobacteraceae bacterium IMCC9480,Pandoraea pnomenusa 3kgm,Pandoraea RB 44,Parasutterella excrementihominis YIT 11859,Simonsiella muelleri ATCC 29453,Sulfuricella denitrificans skB26,Sutterella parvirubra YIT 11816,Sutterella wadsworthensis 2 1 59BFAA,Sutterella wadsworthensis 3 1 45B,beta proteobacterium KB13,Lutiella nitroferrum 2002,Candidatus Glomeribacter gigasporarum,Candidatus Nasuia deltocephalinicola NAS ALF |
| Deltaproteobacteria | Anaeromyxobacter dehalogenans 2CP 1,Anaeromyxobacter dehalogenans 2CP C,Anaeromyxobacter Fw109 5,Anaeromyxobacter K,Bacteriovorax marinus SJ,Bdellovibrio bacteriovorus HD100,Bdellovibrio bacteriovorus Tiberius,Bdellovibrio exovorus JSS,Corallococcus coralloides DSM 2259,Desulfarculus baarsii DSM 2075,Desulfatibacillum alkenivorans AK 01,Desulfobacca acetoxidans DSM 11109,Desulfobacterium autotrophicum HRM2,Desulfobacula toluolica Tol2,Desulfobulbus propionicus DSM 2032,Desulfocapsa sulfexigens DSM 10523,Desulfococcus oleovorans Hxd3,Desulfohalobium retbaense DSM 5692,Desulfomicrobium baculatum DSM 4028,Desulfomonile tiedjei DSM 6799,Desulfotalea psychrophila LSv54,Desulfovibrio 3 1 syn3,Desulfovibrio 6 1 46AFAA,Desulfovibrio A2,Desulfovibrio aespoeensis Aspo 2,Desulfovibrio africanus PCS,Desulfovibrio africanus Walvis Bay,Desulfovibrio alaskensis G20,Desulfovibrio desulfuricans ATCC 27774,Desulfovibrio desulfuricans ND132,Desulfovibrio fructosovorans JJ,Desulfovibrio FW1012B,Desulfovibrio gigas DSM 1382,Desulfovibrio hydrothermalis AM13 DSM 14728,Desulfovibrio magneticus Maddingley MBC34,Desulfovibrio magneticus RS 1,Desulfovibrio piezophilus C1TLV30,Desulfovibrio piger ATCC 29098,Desulfovibrio salexigens DSM 2638,Desulfovibrio U5L,Desulfovibrio vulgaris Miyazaki F,Desulfovibrio vulgaris DP4,Desulfovibrio vulgaris Hildenborough,Desulfovibrio vulgaris RCH1,Desulfurivibrio alkaliphilus AHT2,Geobacter bemidjiensis Bem,Geobacter FRC 32,Geobacter lovleyi SZ,Geobacter M18,Geobacter M21,Geobacter metallireducens GS 15,Geobacter metallireducens RCH3,Geobacter sulfurreducens KN400,Geobacter sulfurreducens PCA,Geobacter uraniireducens Rf4,Haliangium ochraceum DSM 14365,Hippea maritima DSM 10411,Lawsonia intracellularis N343,Lawsonia intracellularis PHE MN1 00,Myxococcus fulvus HW 1,Myxococcus stipitatus DSM 14675,Myxococcus xanthus DK 1622,Pelobacter carbinolicus DSM 2380,Pelobacter propionicus DSM 2379,Sorangium cellulosum So ce 56,Sorangium cellulosum So0157 2,Stigmatella aurantiaca DW4 3 1,Syntrophobacter fumaroxidans MPOB,Syntrophus aciditrophicus SB,Bilophila 4 1 30,Bilophila wadsworthia 3 1 6,Chondromyces apiculatus DSM 436,Desulfonatronospira thiodismutans ASO3 1,Desulfotignum phosphitoxidans DSM 13687,Desulfuromonas acetoxidans DSM 684,Plesiocystis pacifica SIR 1,delta proteobacterium MLMS 1,delta proteobacterium NaphS2,SAR324 cluster bacterium JCVI SC AAA005,SAR324 cluster bacterium SCGC AAA001 C10,Cystobacter fuscus DSM 2262,Desulfobacter postgatei 2ac9 |
| Epsilonproteobacteria | Arcobacter butzleri 7h1h,Arcobacter butzleri ED 1,Arcobacter butzleri RM4018,Arcobacter L,Arcobacter nitrofigilis DSM 7299,Campylobacter 03 427,Campylobacter 10 1 50,Campylobacter coli 1091,Campylobacter coli 1098,Campylobacter coli 111 3,Campylobacter coli 1148,Campylobacter coli 132 6,Campylobacter coli 1417,Campylobacter coli 151 9,Campylobacter coli 15 537360,Campylobacter coli 1891,Campylobacter coli 1909,Campylobacter coli 1948,Campylobacter coli 1957,Campylobacter coli 1961,Campylobacter coli 202 04,Campylobacter coli 2548,Campylobacter coli 2553,Campylobacter coli 2680,Campylobacter coli 2685,Campylobacter coli 2688,Campylobacter coli 2692,Campylobacter coli 2698,Campylobacter coli 317 04,Campylobacter coli 37 05,Campylobacter coli 59 2,Campylobacter coli 67 8,Campylobacter coli 76339,Campylobacter coli 7 1,Campylobacter coli 80352,Campylobacter coli 84 2,Campylobacter coli 86119,Campylobacter coli 90 3,Campylobacter coli CVM N29710,Campylobacter coli H56,Campylobacter coli H6,Campylobacter coli H8,Campylobacter coli H9,Campylobacter coli JV20,Campylobacter coli LMG 23336,Campylobacter coli LMG 23341,Campylobacter coli LMG 23342,Campylobacter coli LMG 23344,Campylobacter coli LMG 9853,Campylobacter coli LMG 9854,Campylobacter coli LMG 9860,Campylobacter coli RM2228,Campylobacter coli Z156,Campylobacter coli Z163,Campylobacter concisus 13826,Campylobacter concisus UNSWCD,Campylobacter curvus 525 92,Campylobacter fetus 82 40,Campylobacter fetus venerealis Azul 94,Campylobacter fetus venerealis NCTC 10354,Campylobacter FOBRC14,Campylobacter gracilis RM3268,Campylobacter hominis ATCC BAA 381,Campylobacter jejuni 00 2425,Campylobacter jejuni 00 2426,Campylobacter jejuni 00 2538,Campylobacter jejuni 00 2544,Campylobacter jejuni 110 21,Campylobacter jejuni 1213,Campylobacter jejuni 129 258,Campylobacter jejuni 1336,Campylobacter jejuni 140 16,Campylobacter jejuni 1577,Campylobacter jejuni 1798,Campylobacter jejuni 1854,Campylobacter jejuni 1893,Campylobacter jejuni 1928,Campylobacter jejuni 1997 1,Campylobacter jejuni 1997 10,Campylobacter jejuni 1997 11,Campylobacter jejuni 1997 14,Campylobacter jejuni 1997 4,Campylobacter jejuni 1997 7,Campylobacter jejuni 2008 1025,Campylobacter jejuni 2008 831,Campylobacter jejuni 2008 872,Campylobacter jejuni 2008 894,Campylobacter jejuni 2008 979,Campylobacter jejuni 2008 988,Campylobacter jejuni 260 94,Campylobacter jejuni 305,Campylobacter jejuni 327,Campylobacter jejuni 4031,Campylobacter jejuni 414,Campylobacter jejuni 51037,Campylobacter jejuni 51494,Campylobacter jejuni 53161,Campylobacter jejuni 55037,Campylobacter jejuni 60004,Campylobacter jejuni 81116,Campylobacter jejuni 81 176,Campylobacter jejuni 84 25,Campylobacter jejuni 86605,Campylobacter jejuni 87330,Campylobacter jejuni 87459,Campylobacter jejuni ATCC 33560,Campylobacter jejuni CF93 6,Campylobacter jejuni CG8421,Campylobacter jejuni CG8486,Campylobacter jejuni D2600,Campylobacter jejuni DFVF1099,Campylobacter jejuni doylei 269 97,Campylobacter jejuni HB93 13,Campylobacter jejuni IA3902,Campylobacter jejuni ICDCCJ07001,Campylobacter jejuni LMG 23210,Campylobacter jejuni LMG 23211,Campylobacter jejuni LMG 23216,Campylobacter jejuni LMG 23218,Campylobacter jejuni LMG 23223,Campylobacter jejuni LMG 23263,Campylobacter jejuni LMG 23264,Campylobacter jejuni LMG 23269,Campylobacter jejuni LMG 23357,Campylobacter jejuni LMG 9081,Campylobacter jejuni LMG 9217,Campylobacter jejuni LMG 9872,Campylobacter jejuni LMG 9879,Campylobacter jejuni M1,Campylobacter jejuni NCTC 11168 ATCC 700819,Campylobacter jejuni NCTC 11168 BN148,Campylobacter jejuni NW,Campylobacter jejuni PT14,Campylobacter jejuni RM1221,Campylobacter jejuni S3,Campylobacter lari RM2100,Campylobacter rectus RM3267,Campylobacter showae CC57C,Campylobacter showae RM3277,Campylobacter upsaliensis JV21,Campylobacter upsaliensis RM3195,Helicobacter acinonychis Sheeba,Helicobacter bilis ATCC 43879,Helicobacter bizzozeronii CCUG 35545,Helicobacter bizzozeronii CIII 1,Helicobacter canadensis MIT 98 5491,Helicobacter cetorum MIT 00 7128,Helicobacter cetorum MIT 99 5656,Helicobacter cinaedi ATCC BAA 847,Helicobacter cinaedi CCUG 18818,Helicobacter cinaedi PAGU611,Helicobacter felis ATCC 49179,Helicobacter heilmannii ASB1 4,Helicobacter hepaticus ATCC 51449,Helicobacter mustelae 12198,Helicobacter pullorum MIT 98 5489,Helicobacter pylori,Helicobacter pylori 2017,Helicobacter pylori 2018,Helicobacter pylori 26695,Helicobacter pylori 35A,Helicobacter pylori 51,Helicobacter pylori 83,Helicobacter pylori 8A3,Helicobacter pylori 908,Helicobacter pylori 98 10,Helicobacter pylori A45,Helicobacter pylori Aklavik117,Helicobacter pylori Aklavik86,Helicobacter pylori B128,Helicobacter pylori B38,Helicobacter pylori B8,Helicobacter pylori BCS100H1,Helicobacter pylori BM012A,Helicobacter pylori BM012S,Helicobacter pylori CCHI 33,Helicobacter pylori CPY1124,Helicobacter pylori CPY1313,Helicobacter pylori CPY1662,Helicobacter pylori CPY1962,Helicobacter pylori CPY3281,Helicobacter pylori CPY6081,Helicobacter pylori CPY6261,Helicobacter pylori CPY6271,Helicobacter pylori CPY6311,Helicobacter pylori Cuz20,Helicobacter pylori ELS37,Helicobacter pylori F16,Helicobacter pylori F30,Helicobacter pylori F32,Helicobacter pylori F57,Helicobacter pylori G27,Helicobacter pylori GAM100Ai,Helicobacter pylori GAM101Biv,Helicobacter pylori GAM103Bi,Helicobacter pylori GAM105Ai,Helicobacter pylori GAM112Ai,Helicobacter pylori GAM114Ai,Helicobacter pylori GAM115Ai,Helicobacter pylori GAM118Bi,Helicobacter pylori GAM119Bi,Helicobacter pylori GAM120Ai,Helicobacter pylori GAM121Aii,Helicobacter pylori GAM201Ai,Helicobacter pylori GAM210Bi,Helicobacter pylori GAM231Ai,Helicobacter pylori GAM239Bi,Helicobacter pylori GAM244Ai,Helicobacter pylori GAM245Ai,Helicobacter pylori GAM246Ai,Helicobacter pylori GAM249T,Helicobacter pylori GAM250AFi,Helicobacter pylori GAM250T,Helicobacter pylori GAM252Bi,Helicobacter pylori GAM252T,Helicobacter pylori GAM254Ai,Helicobacter pylori GAM260ASi,Helicobacter pylori GAM260Bi,Helicobacter pylori GAM260BSi,Helicobacter pylori GAM263BFi,Helicobacter pylori GAM264Ai,Helicobacter pylori GAM265BSii,Helicobacter pylori GAM268Bii,Helicobacter pylori GAM270ASi,Helicobacter pylori GAM42Ai,Helicobacter pylori GAM71Ai,Helicobacter pylori GAM80Ai,Helicobacter pylori GAM83Bi,Helicobacter pylori GAM83T,Helicobacter pylori GAM93Bi,Helicobacter pylori GAM96Ai,Helicobacter pylori Gambia94 24,Helicobacter pylori GAMchJs106B,Helicobacter pylori GAMchJs114i,Helicobacter pylori GAMchJs117Ai,Helicobacter pylori GAMchJs124i,Helicobacter pylori GAMchJs136i,Helicobacter pylori HP116Bi,Helicobacter pylori HP250AFii,Helicobacter pylori HP250AFiii,Helicobacter pylori HP250AFiV,Helicobacter pylori HP250ASi,Helicobacter pylori HP250ASii,Helicobacter pylori HP250BFi,Helicobacter pylori HP250BFii,Helicobacter pylori HP250BFiii,Helicobacter pylori HP250BFiV,Helicobacter pylori HP250BSi,Helicobacter pylori HP260AFi,Helicobacter pylori HP260AFii,Helicobacter pylori HP260ASii,Helicobacter pylori HP260BFii,Helicobacter pylori HP260Bi,Helicobacter pylori Hp A 14,Helicobacter pylori Hp A 16,Helicobacter pylori Hp A 17,Helicobacter pylori Hp A 20,Helicobacter pylori Hp A 26,Helicobacter pylori Hp A 27,Helicobacter pylori Hp A 4,Helicobacter pylori Hp A 5,Helicobacter pylori Hp A 6,Helicobacter pylori Hp A 8,Helicobacter pylori Hp A 9,Helicobacter pylori Hp H 1,Helicobacter pylori Hp H 10,Helicobacter pylori Hp H 11,Helicobacter pylori Hp H 16,Helicobacter pylori Hp H 18,Helicobacter pylori Hp H 19,Helicobacter pylori Hp H 21,Helicobacter pylori Hp H 23,Helicobacter pylori Hp H 24,Helicobacter pylori Hp H 24b,Helicobacter pylori Hp H 24c,Helicobacter pylori Hp H 27,Helicobacter pylori Hp H 28,Helicobacter pylori Hp H 29,Helicobacter pylori Hp H 3,Helicobacter pylori Hp H 30,Helicobacter pylori Hp H 34,Helicobacter pylori Hp H 36,Helicobacter pylori Hp H 4,Helicobacter pylori Hp H 41,Helicobacter pylori Hp H 42,Helicobacter pylori Hp H 43,Helicobacter pylori Hp H 44,Helicobacter pylori Hp H 45,Helicobacter pylori Hp H 5b,Helicobacter pylori Hp H 6,Helicobacter pylori Hp H 9,Helicobacter pylori Hp M1,Helicobacter pylori Hp M2,Helicobacter pylori Hp M3,Helicobacter pylori Hp M4,Helicobacter pylori Hp M5,Helicobacter pylori Hp M6,Helicobacter pylori Hp M9,Helicobacter pylori Hp P 1,Helicobacter pylori Hp P 11,Helicobacter pylori Hp P 11b,Helicobacter pylori Hp P 13,Helicobacter pylori Hp P 13b,Helicobacter pylori Hp P 15,Helicobacter pylori Hp P 15b,Helicobacter pylori Hp P 16,Helicobacter pylori Hp P 1b,Helicobacter pylori Hp P 2,Helicobacter pylori Hp P 23,Helicobacter pylori Hp P 25,Helicobacter pylori Hp P 25c,Helicobacter pylori Hp P 25d,Helicobacter pylori Hp P 26,Helicobacter pylori Hp P 28b,Helicobacter pylori Hp P 2b,Helicobacter pylori Hp P 3,Helicobacter pylori Hp P 30,Helicobacter pylori Hp P 3b,Helicobacter pylori Hp P 4,Helicobacter pylori Hp P 41,Helicobacter pylori Hp P 4c,Helicobacter pylori Hp P 4d,Helicobacter pylori Hp P 62,Helicobacter pylori Hp P 74,Helicobacter pylori Hp P 8,Helicobacter pylori Hp P 8b,Helicobacter pylori HPAG1,Helicobacter pylori HPKX 438 AG0C1,Helicobacter pylori HPKX 438 CA4C1,Helicobacter pylori HUP B14,Helicobacter pylori India7,Helicobacter pylori J99,Helicobacter pylori Lithuania75,Helicobacter pylori MALT,Helicobacter pylori N6,Helicobacter pylori NAB47,Helicobacter pylori NAD1,Helicobacter pylori NCTC 11638,Helicobacter pylori NQ1671,Helicobacter pylori NQ1701,Helicobacter pylori NQ1707,Helicobacter pylori NQ1712,Helicobacter pylori NQ315,Helicobacter pylori NQ352,Helicobacter pylori NQ367,Helicobacter pylori NQ392,Helicobacter pylori NQ4044,Helicobacter pylori NQ4053,Helicobacter pylori NQ4060,Helicobacter pylori NQ4076,Helicobacter pylori NQ4099,Helicobacter pylori NQ4110,Helicobacter pylori NQ4161,Helicobacter pylori NQ4191,Helicobacter pylori NQ4200,Helicobacter pylori NQ4216,Helicobacter pylori NQ4228,Helicobacter pylori OK113,Helicobacter pylori OK310,Helicobacter pylori P12,Helicobacter pylori P79,Helicobacter pylori PeCan18,Helicobacter pylori PeCan4,Helicobacter pylori Puno120,Helicobacter pylori Puno135,Helicobacter pylori R018c,Helicobacter pylori R030b,Helicobacter pylori R036d,Helicobacter pylori R037c,Helicobacter pylori R038b,Helicobacter pylori R046Wa,Helicobacter pylori R055a,Helicobacter pylori R056a,Helicobacter pylori R32b,Helicobacter pylori Rif1,Helicobacter pylori Rif2,Helicobacter pylori Sat464,Helicobacter pylori Shi112,Helicobacter pylori Shi169,Helicobacter pylori Shi417,Helicobacter pylori Shi470,Helicobacter pylori SJM180,Helicobacter pylori SNT49,Helicobacter pylori SouthAfrica20,Helicobacter pylori SouthAfrica7,Helicobacter pylori UM032,Helicobacter pylori UM037,Helicobacter pylori UM066,Helicobacter pylori UM298,Helicobacter pylori UM299,Helicobacter pylori v225d,Helicobacter pylori XZ274,Helicobacter suis HS1,Helicobacter suis HS5,Helicobacter winghamensis ATCC BAA 430,Nautilia profundicola AmH,Nitratifractor salsuginis DSM 16511,Nitratiruptor SB155 2,Sulfuricurvum kujiense DSM 16994,Sulfurimonas autotrophica DSM 16294,Sulfurimonas denitrificans DSM 1251,Sulfurimonas GD1,Sulfurospirillum barnesii SES 3,Sulfurospirillum deleyianum DSM 6946,Sulfurovum AR,Sulfurovum NBC37 1,Wolinella succinogenes DSM 1740,Caminibacter mediatlanticus TB 2,Campylobacterales bacterium GD 1,Thiovulum ES |
| Gammaproteobacteria | Acidithiobacillus caldus SM 1,Acidithiobacillus ferrivorans SS3,Acidithiobacillus ferrooxidans ATCC 23270,Acidithiobacillus ferrooxidans ATCC 53993,Acinetobacter ADP1,Acinetobacter baumannii 1656 2,Acinetobacter baumannii AB0057,Acinetobacter baumannii AB307 0294,Acinetobacter baumannii ACICU,Acinetobacter baumannii ATCC 17978,Acinetobacter baumannii AYE,Acinetobacter baumannii BJAB07104,Acinetobacter baumannii BJAB0715,Acinetobacter baumannii BJAB0868,Acinetobacter baumannii D1279779,Acinetobacter baumannii MDR TJ,Acinetobacter baumannii MDR ZJ06,Acinetobacter baumannii SDF,Acinetobacter baumannii TCDC AB0715,Acinetobacter baumannii TYTH 1,Acinetobacter baumannii ZW85 1,Acinetobacter calcoaceticus PHEA 2,Acinetobacter oleivorans DR1,Actinobacillus pleuropneumoniae serovar 3 JL03,Actinobacillus pleuropneumoniae serovar 5b L20,Actinobacillus pleuropneumoniae serovar 7 AP76,Actinobacillus succinogenes 130Z,Actinobacillus suis H91 0380,Aeromonas hydrophila ATCC 7966,Aeromonas hydrophila ML09 119,Aeromonas salmonicida A449,Aeromonas veronii B565,Aggregatibacter actinomycetemcomitans ANH9381,Aggregatibacter actinomycetemcomitans D11S 1,Aggregatibacter actinomycetemcomitans D7S 1,Aggregatibacter aphrophilus NJ8700,Alcanivorax borkumensis SK2,Alcanivorax dieselolei B5,Aliivibrio salmonicida LFI1238,Alkalilimnicola ehrlichii MLHE 1,Allochromatium vinosum DSM 180,Alteromonas macleodii Aegean Sea MED64,Alteromonas macleodii Balearic Sea AD45,Alteromonas macleodii Black Sea 11,Alteromonas macleodii Deep ecotype,Alteromonas macleodii English Channel 615,Alteromonas macleodii English Channel 673,Alteromonas macleodii Ionian Sea U4,Alteromonas macleodii Ionian Sea U7,Alteromonas macleodii Ionian Sea U8,Alteromonas macleodii Ionian Sea UM4b,Alteromonas macleodii Ionian Sea UM7,Alteromonas macleodii AltDE1,Alteromonas macleodii ATCC 27126,Alteromonas SN2,Azotobacter vinelandii CA,Azotobacter vinelandii CA6,Azotobacter vinelandii DJ,Baumannia cicadellinicola Hc Homalodisca coagulata,Bibersteinia trehalosi 192,Buchnera aphidicola 5A Acyrthosiphon pisum,Buchnera aphidicola Cinara tujafilina,Buchnera aphidicola Ak Acyrthosiphon kondoi,Buchnera aphidicola APS Acyrthosiphon pisum,Buchnera aphidicola Bp Baizongia pistaciae,Buchnera aphidicola Cc Cinara cedri,Buchnera aphidicola JF98 Acyrthosiphon pisum,Buchnera aphidicola JF99 Acyrthosiphon pisum,Buchnera aphidicola LL01 Acyrthosiphon pisum,Buchnera aphidicola LSR1 Acyrthosiphon pisum,Buchnera aphidicola Sg Schizaphis graminum,Buchnera aphidicola TLW03 Acyrthosiphon pisum,Buchnera aphidicola Tuc7 Acyrthosiphon pisum,Buchnera aphidicola Ua Uroleucon ambrosiae,Candidatus Blochmannia chromaiodes 640,Candidatus Blochmannia floridanus,Candidatus Blochmannia pennsylvanicus BPEN,Candidatus Blochmannia vafer BVAF,Candidatus Carsonella ruddii,Candidatus Carsonella ruddii CE isolate Thao2000,Candidatus Carsonella ruddii CS isolate Thao2000,Candidatus Carsonella ruddii DC,Candidatus Carsonella ruddii HC isolate Thao2000,Candidatus Carsonella ruddii HT isolate Thao2000,Candidatus Carsonella ruddii PC isolate NHV,Candidatus Hamiltonella defensa 5AT Acyrthosiphon pisum,Candidatus Moranella endobia PCIT,Candidatus Moranella endobia PCVAL,Candidatus Portiera aleyrodidarum BT B,Candidatus Portiera aleyrodidarum BT QVLC,Candidatus Portiera aleyrodidarum TV,Candidatus Riesia pediculicola USDA,Candidatus Ruthia magnifica Cm Calyptogena magnifica,Candidatus Vesicomyosocius okutanii HA,Cellvibrio BR,Cellvibrio japonicus Ueda107,Chromohalobacter salexigens DSM 3043,Citrobacter 30 2,Citrobacter A1,Citrobacter freundii 4 7 47CFAA,Citrobacter freundii ATCC 8090 MTCC 1658,Citrobacter freundii GTC 09479,Citrobacter koseri ATCC BAA 895,Citrobacter rodentium ICC168,Citrobacter youngae ATCC 29220,Colwellia psychrerythraea 34H,Coxiella burnetii MSU Goat Q177,Coxiella burnetii CbuG Q212,Coxiella burnetii CbuK Q154,Coxiella burnetii Dugway 5J108 111,Coxiella burnetii RSA 331,Coxiella burnetii RSA 334,Coxiella burnetii RSA 493,Cronobacter sakazakii 45402,Cronobacter sakazakii ATCC BAA 894,Cronobacter sakazakii E899,Cronobacter sakazakii ES15,Cronobacter sakazakii Sp291,Cronobacter turicensis z3032,Cycloclasticus P1,Cycloclasticus zancles 7 ME,Dichelobacter nodosus VCS1703A,Dickeya dadantii 3937,Dickeya dadantii Ech586,Dickeya dadantii Ech703,Dickeya zeae Ech1591,Edwardsiella ictaluri 93 146,Edwardsiella tarda ATCC 23685,Edwardsiella tarda C07 087,Edwardsiella tarda EIB202,Edwardsiella tarda FL6 60,Edwardsiella tarda NBRC 105688,Enterobacter 638,Enterobacter aerogenes EA1509E,Enterobacter aerogenes KCTC 2190,Enterobacter Ag1,Enterobacter asburiae LF7a,Enterobacter cancerogenus ATCC 35316,Enterobacter cloacae ATCC 13047,Enterobacter cloacae dissolvens SDM,Enterobacter cloacae EcWSU1,Enterobacter cloacae ENHKU01,Enterobacter cloacae GS1,Enterobacter cloacae NCTC 9394,Enterobacter cloacae SCF1,Enterobacter hormaechei ATCC 49162,Enterobacter mori LMG 25706,Enterobacter R4 368,Enterobacter radicincitans DSM 16656,Enterobacter SST3,Enterobacteriaceae bacterium 9 2 54FAA,Enterobacteriaceae bacterium FGI 57,Erwinia amylovora ATCC 49946,Erwinia amylovora CFBP1430,Erwinia billingiae Eb661,Erwinia Ejp617,Erwinia pyrifoliae DSM 12163,Erwinia pyrifoliae Ep1 96,Erwinia tasmaniensis Et1 99,Escherichia 1 1 43,Escherichia 3 2 53FAA,Escherichia 4 1 40B,Escherichia albertii TW07627,Escherichia albertii TW08933,Escherichia albertii TW11588,Escherichia albertii TW15818,Escherichia blattae DSM 4481,Escherichia blattae NBRC 105725,Escherichia coli 042,Escherichia coli 07798,Escherichia coli 09BKT078844,Escherichia coli 0 1288,Escherichia coli 0 1304,Escherichia coli 101 1,Escherichia coli 10 0821,Escherichia coli 10 0833,Escherichia coli 10 0869,Escherichia coli 1827 70,Escherichia coli 1 2264,Escherichia coli 1 2741,Escherichia coli 2362 75,Escherichia coli 2534 86,Escherichia coli 2 3916,Escherichia coli 2 4168,Escherichia coli 3003,Escherichia coli 3006,Escherichia coli 3030 1,Escherichia coli 3431,Escherichia coli 3 2303,Escherichia coli 3 2608,Escherichia coli 3 3884,Escherichia coli 3 4870,Escherichia coli 3 4880,Escherichia coli 4 0522,Escherichia coli 4 0967,Escherichia coli 4 1 47FAA,Escherichia coli 536,Escherichia coli 53638,Escherichia coli 5412,Escherichia coli 541 1,Escherichia coli 541 15,Escherichia coli 55989,Escherichia coli 576 1,Escherichia coli 5905,Escherichia coli 5 0588,Escherichia coli 5 0959,Escherichia coli 5 2239,Escherichia coli 6 0172,Escherichia coli 75,Escherichia coli 7 1982,Escherichia coli 83972,Escherichia coli 88 0221,Escherichia coli 88 1042,Escherichia coli 88 1467,Escherichia coli 89 0511,Escherichia coli 8 0416,Escherichia coli 8 0566,Escherichia coli 8 0569,Escherichia coli 8 0586,Escherichia coli 8 2524,Escherichia coli 900105 10e,Escherichia coli 90 0039,Escherichia coli 90 0091,Escherichia coli 90 2281,Escherichia coli 93 001,Escherichia coli 93 0055,Escherichia coli 93 0056,Escherichia coli 93 0624,Escherichia coli 94 0618,Escherichia coli 95 0083,Escherichia coli 95 0183,Escherichia coli 95 0941,Escherichia coli 95 0943,Escherichia coli 95 1288,Escherichia coli 96 0107,Escherichia coli 96 0109,Escherichia coli 96 0427,Escherichia coli 96 0428,Escherichia coli 96 0497,Escherichia coli 96 0932,Escherichia coli 96 0939,Escherichia coli 96 154,Escherichia coli 97 0003,Escherichia coli 97 0010,Escherichia coli 97 0246,Escherichia coli 97 0259,Escherichia coli 97 0264,Escherichia coli 97 1742,Escherichia coli 99 0670,Escherichia coli 99 0672,Escherichia coli 99 0741,Escherichia coli 99 0814,Escherichia coli 99 0815,Escherichia coli 99 0816,Escherichia coli 99 0839,Escherichia coli 99 0848,Escherichia coli 99 1753,Escherichia coli 99 1762,Escherichia coli 99 1775,Escherichia coli 99 1781,Escherichia coli 99 1793,Escherichia coli 99 1805,Escherichia coli 9 0111,Escherichia coli 9 1649,Escherichia coli BL21 Gold DE3 pLysS AG,Escherichia coli clone D i14,Escherichia coli clone D i2,Escherichia coli AA86,Escherichia coli ABU 83972,Escherichia coli AD30,Escherichia coli AI27,Escherichia coli APEC O1,Escherichia coli APEC O78,Escherichia coli ARS4 2123,Escherichia coli ATCC 700728,Escherichia coli ATCC 8739,Escherichia coli B088,Escherichia coli B093,Escherichia coli B171,Escherichia coli B185,Escherichia coli B354,Escherichia coli B41,Escherichia coli B799,Escherichia coli B7A,Escherichia coli B REL606,Escherichia coli BL21 DE3,Escherichia coli BW2952,Escherichia coli CB7326,Escherichia coli CFT073,Escherichia coli chi7122,Escherichia coli cloneA i1,Escherichia coli CUMT8,Escherichia coli DEC10A,Escherichia coli DEC10B,Escherichia coli DEC10C,Escherichia coli DEC10D,Escherichia coli DEC10E,Escherichia coli DEC10F,Escherichia coli DEC11A,Escherichia coli DEC11B,Escherichia coli DEC11C,Escherichia coli DEC11D,Escherichia coli DEC11E,Escherichia coli DEC12A,Escherichia coli DEC12B,Escherichia coli DEC12C,Escherichia coli DEC12D,Escherichia coli DEC12E,Escherichia coli DEC13A,Escherichia coli DEC13B,Escherichia coli DEC13C,Escherichia coli DEC13D,Escherichia coli DEC13E,Escherichia coli DEC14A,Escherichia coli DEC14B,Escherichia coli DEC14C,Escherichia coli DEC14D,Escherichia coli DEC15A,Escherichia coli DEC15B,Escherichia coli DEC15C,Escherichia coli DEC15D,Escherichia coli DEC15E,Escherichia coli DEC1A,Escherichia coli DEC1B,Escherichia coli DEC1C,Escherichia coli DEC1D,Escherichia coli DEC1E,Escherichia coli DEC2A,Escherichia coli DEC2B,Escherichia coli DEC2C,Escherichia coli DEC2D,Escherichia coli DEC2E,Escherichia coli DEC3A,Escherichia coli DEC3B,Escherichia coli DEC3C,Escherichia coli DEC3D,Escherichia coli DEC3E,Escherichia coli DEC3F,Escherichia coli DEC4A,Escherichia coli DEC4B,Escherichia coli DEC4C,Escherichia coli DEC4D,Escherichia coli DEC4E,Escherichia coli DEC4F,Escherichia coli DEC5A,Escherichia coli DEC5B,Escherichia coli DEC5C,Escherichia coli DEC5D,Escherichia coli DEC5E,Escherichia coli DEC6A,Escherichia coli DEC6B,Escherichia coli DEC6C,Escherichia coli DEC6D,Escherichia coli DEC6E,Escherichia coli DEC7A,Escherichia coli DEC7B,Escherichia coli DEC7C,Escherichia coli DEC7D,Escherichia coli DEC7E,Escherichia coli DEC8A,Escherichia coli DEC8B,Escherichia coli DEC8C,Escherichia coli DEC8D,Escherichia coli DEC8E,Escherichia coli DEC9A,Escherichia coli DEC9B,Escherichia coli DEC9C,Escherichia coli DEC9D,Escherichia coli DEC9E,Escherichia coli DH1,Escherichia coli E101,Escherichia coli E110019,Escherichia coli E1167,Escherichia coli E128010,Escherichia coli E1520,Escherichia coli E22,Escherichia coli E24377A,Escherichia coli E482,Escherichia coli EC1734,Escherichia coli EC1735,Escherichia coli EC1736,Escherichia coli EC1737,Escherichia coli EC1738,Escherichia coli EC1845,Escherichia coli EC1846,Escherichia coli EC1847,Escherichia coli EC1848,Escherichia coli EC1849,Escherichia coli EC1850,Escherichia coli EC1856,Escherichia coli EC1862,Escherichia coli EC1863,Escherichia coli EC1864,Escherichia coli EC1865,Escherichia coli EC1866,Escherichia coli EC1868,Escherichia coli EC1869,Escherichia coli EC1870,Escherichia coli EC302 04,Escherichia coli EC4013,Escherichia coli EC4100B,Escherichia coli EC4196,Escherichia coli EC4203,Escherichia coli EC4402,Escherichia coli EC4421,Escherichia coli EC4422,Escherichia coli EC4436,Escherichia coli EC4437,Escherichia coli EC4439,Escherichia coli EC4448,Escherichia coli EC96038,Escherichia coli ED1a,Escherichia coli EPEC C342 62,Escherichia coli EPECa12,Escherichia coli EPECa14,Escherichia coli ETEC H10407,Escherichia coli F11,Escherichia coli FDA504,Escherichia coli FDA505,Escherichia coli FDA506,Escherichia coli FDA507,Escherichia coli FDA517,Escherichia coli FRIK1985,Escherichia coli FRIK1990,Escherichia coli FRIK1996,Escherichia coli FRIK1997,Escherichia coli FRIK1999,Escherichia coli FRIK2001,Escherichia coli FRIK523,Escherichia coli FRIK920,Escherichia coli FVEC1302,Escherichia coli FVEC1412,Escherichia coli G58 1,Escherichia coli H120,Escherichia coli H252,Escherichia coli H263,Escherichia coli H299,Escherichia coli H30,Escherichia coli H397,Escherichia coli H489,Escherichia coli H494,Escherichia coli H591,Escherichia coli H730,Escherichia coli H736,Escherichia coli HM605,Escherichia coli HS,Escherichia coli IAI1,Escherichia coli IAI39,Escherichia coli IHE3034,Escherichia coli IMT2125,Escherichia coli J53,Escherichia coli J96,Escherichia coli JB1 95,Escherichia coli JJ1886,Escherichia coli K 12 substr DH10B,Escherichia coli K 12 substr MDS42,Escherichia coli K 12 substr MG1655,Escherichia coli K 12 substr MG1655star,Escherichia coli K 12 substr W3110,Escherichia coli KD1,Escherichia coli KD2,Escherichia coli KO11FL,Escherichia coli KTE10,Escherichia coli KTE101,Escherichia coli KTE104,Escherichia coli KTE105,Escherichia coli KTE106,Escherichia coli KTE109,Escherichia coli KTE11,Escherichia coli KTE111,Escherichia coli KTE112,Escherichia coli KTE113,Escherichia coli KTE115,Escherichia coli KTE116,Escherichia coli KTE117,Escherichia coli KTE118,Escherichia coli KTE119,Escherichia coli KTE12,Escherichia coli KTE120,Escherichia coli KTE122,Escherichia coli KTE123,Escherichia coli KTE124,Escherichia coli KTE125,Escherichia coli KTE128,Escherichia coli KTE129,Escherichia coli KTE131,Escherichia coli KTE133,Escherichia coli KTE135,Escherichia coli KTE136,Escherichia coli KTE137,Escherichia coli KTE138,Escherichia coli KTE139,Escherichia coli KTE140,Escherichia coli KTE141,Escherichia coli KTE142,Escherichia coli KTE143,Escherichia coli KTE144,Escherichia coli KTE145,Escherichia coli KTE146,Escherichia coli KTE147,Escherichia coli KTE148,Escherichia coli KTE15,Escherichia coli KTE150,Escherichia coli KTE153,Escherichia coli KTE154,Escherichia coli KTE156,Escherichia coli KTE157,Escherichia coli KTE158,Escherichia coli KTE16,Escherichia coli KTE160,Escherichia coli KTE161,Escherichia coli KTE162,Escherichia coli KTE163,Escherichia coli KTE165,Escherichia coli KTE166,Escherichia coli KTE167,Escherichia coli KTE168,Escherichia coli KTE169,Escherichia coli KTE17,Escherichia coli KTE171,Escherichia coli KTE173,Escherichia coli KTE174,Escherichia coli KTE175,Escherichia coli KTE176,Escherichia coli KTE177,Escherichia coli KTE178,Escherichia coli KTE179,Escherichia coli KTE18,Escherichia coli KTE180,Escherichia coli KTE181,Escherichia coli KTE183,Escherichia coli KTE184,Escherichia coli KTE187,Escherichia coli KTE188,Escherichia coli KTE189,Escherichia coli KTE190,Escherichia coli KTE191,Escherichia coli KTE192,Escherichia coli KTE193,Escherichia coli KTE194,Escherichia coli KTE196,Escherichia coli KTE197,Escherichia coli KTE2,Escherichia coli KTE201,Escherichia coli KTE202,Escherichia coli KTE203,Escherichia coli KTE204,Escherichia coli KTE205,Escherichia coli KTE206,Escherichia coli KTE207,Escherichia coli KTE208,Escherichia coli KTE209,Escherichia coli KTE21,Escherichia coli KTE210,Escherichia coli KTE211,Escherichia coli KTE212,Escherichia coli KTE213,Escherichia coli KTE214,Escherichia coli KTE215,Escherichia coli KTE216,Escherichia coli KTE217,Escherichia coli KTE218,Escherichia coli KTE22,Escherichia coli KTE220,Escherichia coli KTE223,Escherichia coli KTE224,Escherichia coli KTE227,Escherichia coli KTE228,Escherichia coli KTE229,Escherichia coli KTE23,Escherichia coli KTE230,Escherichia coli KTE232,Escherichia coli KTE233,Escherichia coli KTE234,Escherichia coli KTE235,Escherichia coli KTE236,Escherichia coli KTE237,Escherichia coli KTE25,Escherichia coli KTE26,Escherichia coli KTE28,Escherichia coli KTE29,Escherichia coli KTE39,Escherichia coli KTE4,Escherichia coli KTE42,Escherichia coli KTE43,Escherichia coli KTE44,Escherichia coli KTE45,Escherichia coli KTE46,Escherichia coli KTE47,Escherichia coli KTE48,Escherichia coli KTE49,Escherichia coli KTE5,Escherichia coli KTE50,Escherichia coli KTE51,Escherichia coli KTE53,Escherichia coli KTE54,Escherichia coli KTE55,Escherichia coli KTE56,Escherichia coli KTE57,Escherichia coli KTE58,Escherichia coli KTE59,Escherichia coli KTE6,Escherichia coli KTE60,Escherichia coli KTE62,Escherichia coli KTE63,Escherichia coli KTE65,Escherichia coli KTE66,Escherichia coli KTE67,Escherichia coli KTE72,Escherichia coli KTE75,Escherichia coli KTE76,Escherichia coli KTE77,Escherichia coli KTE78,Escherichia coli KTE79,Escherichia coli KTE8,Escherichia coli KTE80,Escherichia coli KTE81,Escherichia coli KTE82,Escherichia coli KTE83,Escherichia coli KTE84,Escherichia coli KTE85,Escherichia coli KTE86,Escherichia coli KTE87,Escherichia coli KTE88,Escherichia coli KTE9,Escherichia coli KTE90,Escherichia coli KTE91,Escherichia coli KTE93,Escherichia coli KTE94,Escherichia coli KTE95,Escherichia coli KTE97,Escherichia coli KTE99,Escherichia coli LCT EC106,Escherichia coli LF82,Escherichia coli LT 68,Escherichia coli LY180,Escherichia coli M605,Escherichia coli M718,Escherichia coli M863,Escherichia coli M919,Escherichia coli MA6,Escherichia coli MS 107 1,Escherichia coli MS 110 3,Escherichia coli MS 115 1,Escherichia coli MS 116 1,Escherichia coli MS 117 3,Escherichia coli MS 119 7,Escherichia coli MS 124 1,Escherichia coli MS 145 7,Escherichia coli MS 146 1,Escherichia coli MS 153 1,Escherichia coli MS 16 3,Escherichia coli MS 175 1,Escherichia coli MS 182 1,Escherichia coli MS 185 1,Escherichia coli MS 187 1,Escherichia coli MS 196 1,Escherichia coli MS 198 1,Escherichia coli MS 200 1,Escherichia coli MS 21 1,Escherichia coli MS 45 1,Escherichia coli MS 57 2,Escherichia coli MS 60 1,Escherichia coli MS 69 1,Escherichia coli MS 78 1,Escherichia coli MS 79 10,Escherichia coli MS 84 1,Escherichia coli MS 85 1,Escherichia coli N1,Escherichia coli NA114,Escherichia coli NC101,Escherichia coli NCCP15647,Escherichia coli NCCP15657,Escherichia coli NCCP15658,Escherichia coli NE037,Escherichia coli NE098,Escherichia coli NE1487,Escherichia coli Nissle 1917,Escherichia coli O08,Escherichia coli O103 H25 CVM9340,Escherichia coli O103 H25 NIPH 11060424,Escherichia coli O103 H2 12009,Escherichia coli O103 H2 CVM9450,Escherichia coli O104 H4 01 09591,Escherichia coli O104 H4 04 8351,Escherichia coli O104 H4 09 7901,Escherichia coli O104 H4 11 02030,Escherichia coli O104 H4 11 02033 1,Escherichia coli O104 H4 11 02092,Escherichia coli O104 H4 11 02093,Escherichia coli O104 H4 11 02281,Escherichia coli O104 H4 11 02318,Escherichia coli O104 H4 11 02913,Escherichia coli O104 H4 11 03439,Escherichia coli O104 H4 11 03943,Escherichia coli O104 H4 11 04080,Escherichia coli O104 H4 11 3677,Escherichia coli O104 H4 11 4404,Escherichia coli O104 H4 11 4522,Escherichia coli O104 H4 11 4623,Escherichia coli O104 H4 11 4632 C1,Escherichia coli O104 H4 11 4632 C2,Escherichia coli O104 H4 11 4632 C3,Escherichia coli O104 H4 11 4632 C4,Escherichia coli O104 H4 11 4632 C5,Escherichia coli O104 H4 2009EL 2050,Escherichia coli O104 H4 2009EL 2071,Escherichia coli O104 H4 2011C 3493,Escherichia coli O104 H4 C227 11,Escherichia coli O104 H4 C236 11,Escherichia coli O104 H4 E112 10,Escherichia coli O104 H4 Ec11 4984,Escherichia coli O104 H4 Ec11 4986,Escherichia coli O104 H4 Ec11 4987,Escherichia coli O104 H4 Ec11 4988,Escherichia coli O104 H4 Ec11 5603,Escherichia coli O104 H4 Ec11 5604,Escherichia coli O104 H4 Ec11 6006,Escherichia coli O104 H4 Ec11 9450,Escherichia coli O104 H4 Ec11 9941,Escherichia coli O104 H4 Ec11 9990,Escherichia coli O104 H4 Ec12 0465,Escherichia coli O104 H4 Ec12 0466,Escherichia coli O104 H4 GOS1,Escherichia coli O104 H4 GOS2,Escherichia coli O104 H4 H112180280,Escherichia coli O104 H4 H112180282,Escherichia coli O104 H4 H112180283,Escherichia coli O104 H4 LB226692,Escherichia coli O104 H4 ON2010,Escherichia coli O104 H4 ON2011,Escherichia coli O104 H4 TY 2482,Escherichia coli O10 K5 L H4 ATCC 23506,Escherichia coli O111 B4 C48 93,Escherichia coli O111 CVM9455,Escherichia coli O111 H11 CVM9534,Escherichia coli O111 H11 CVM9545,Escherichia coli O111 H11 CVM9553,Escherichia coli O111 H8 CVM9570,Escherichia coli O111 H8 CVM9574,Escherichia coli O111 H8 CVM9602,Escherichia coli O111 H8 CVM9634,Escherichia coli O111 H 11128,Escherichia coli O113 H21 CL 3,Escherichia coli O121 H19 MT 2,Escherichia coli O127 B8 C43 90,Escherichia coli O127 H6 E2348 69,Escherichia coli O145 H28 4865 96,Escherichia coli O157 H43 T22,Escherichia coli O157 H7 1044,Escherichia coli O157 H7 1125,Escherichia coli O157 H7 EC1212,Escherichia coli O157 H7 EC4009,Escherichia coli O157 H7 EC4024,Escherichia coli O157 H7 EC4042,Escherichia coli O157 H7 EC4045,Escherichia coli O157 H7 EC4076,Escherichia coli O157 H7 EC4113,Escherichia coli O157 H7 EC4115,Escherichia coli O157 H7 EC4191,Escherichia coli O157 H7 EC4196,Escherichia coli O157 H7 EC4206,Escherichia coli O157 H7 EC4401,Escherichia coli O157 H7 EC4486,Escherichia coli O157 H7 EC4501,Escherichia coli O157 H7 EC508,Escherichia coli O157 H7 EC869,Escherichia coli O157 H7 EDL933,Escherichia coli O157 H7 FRIK2000,Escherichia coli O157 H7 FRIK966,Escherichia coli O157 H7 G5101,Escherichia coli O157 H7 LSU 61,Escherichia coli O157 H7 Sakai,Escherichia coli O157 H7 TW14359,Escherichia coli O157 H7 TW14588,Escherichia coli O157 H 493 89,Escherichia coli O157 H H 2687,Escherichia coli O25b H4 ST131 EC958,Escherichia coli O26 H11 11368,Escherichia coli O26 H11 CVM10021,Escherichia coli O26 H11 CVM10026,Escherichia coli O26 H11 CVM10030,Escherichia coli O26 H11 CVM10224,Escherichia coli O26 H11 CVM9942,Escherichia coli O26 H11 CVM9952,Escherichia coli O32 H37 P4,Escherichia coli O45 H2 03 EN 705,Escherichia coli O55 H7 3256 97,Escherichia coli O55 H7 CB9615,Escherichia coli O55 H7 RM12579,Escherichia coli O55 H7 USDA 5905,Escherichia coli O5 K4 L H4 ATCC 23502,Escherichia coli O7 K1 CE10,Escherichia coli O83 H1 NRG 857C,Escherichia coli O91 H21 B2F1,Escherichia coli OK1180,Escherichia coli OK1357,Escherichia coli OP50,Escherichia coli P12b,Escherichia coli PA10,Escherichia coli PA11,Escherichia coli PA13,Escherichia coli PA14,Escherichia coli PA15,Escherichia coli PA19,Escherichia coli PA2,Escherichia coli PA22,Escherichia coli PA23,Escherichia coli PA24,Escherichia coli PA25,Escherichia coli PA28,Escherichia coli PA3,Escherichia coli PA31,Escherichia coli PA32,Escherichia coli PA33,Escherichia coli PA34,Escherichia coli PA35,Escherichia coli PA38,Escherichia coli PA39,Escherichia coli PA4,Escherichia coli PA40,Escherichia coli PA41,Escherichia coli PA42,Escherichia coli PA45,Escherichia coli PA47,Escherichia coli PA48,Escherichia coli PA49,Escherichia coli PA5,Escherichia coli PA7,Escherichia coli PA8,Escherichia coli PA9,Escherichia coli PCN033,Escherichia coli PMV 1,Escherichia coli RN587 1,Escherichia coli S17,Escherichia coli S88,Escherichia coli SCI 07,Escherichia coli SE11,Escherichia coli SE15,Escherichia coli SEPT362,Escherichia coli SMS 3 5,Escherichia coli STEC 7v,Escherichia coli STEC 94C,Escherichia coli STEC B2F1,Escherichia coli STEC C165 02,Escherichia coli STEC DG131 3,Escherichia coli STEC EH250,Escherichia coli STEC H 1 8,Escherichia coli STEC MHI813,Escherichia coli STEC O31,Escherichia coli STEC S1191,Escherichia coli TA007,Escherichia coli TA124,Escherichia coli TA143,Escherichia coli TA206,Escherichia coli TA271,Escherichia coli TA280,Escherichia coli TT12B,Escherichia coli TW00353,Escherichia coli TW06591,Escherichia coli TW07793,Escherichia coli TW07945,Escherichia coli TW09098,Escherichia coli TW09109,Escherichia coli TW09195,Escherichia coli TW10119,Escherichia coli TW10246,Escherichia coli TW10509,Escherichia coli TW10598,Escherichia coli TW10722,Escherichia coli TW10828,Escherichia coli TW11039,Escherichia coli TW14301,Escherichia coli TW14313,Escherichia coli TW14425,Escherichia coli TW15901,Escherichia coli TX1999,Escherichia coli UM146,Escherichia coli UMN026,Escherichia coli UMNF18,Escherichia coli UMNK88,Escherichia coli UTI89,Escherichia coli W,Escherichia coli W26,Escherichia coli WV 060327,Escherichia coli XH001,Escherichia coli XH140A,Escherichia coli Xuzhou21,Escherichia fergusonii ATCC 35469,Escherichia fergusonii B253,Escherichia fergusonii ECD227,Escherichia hermannii NBRC 105704,Escherichia TW09231,Escherichia TW09276,Escherichia TW09308,Escherichia TW10509,Ferrimonas balearica DSM 9799,Francisella cf novicida 3523,Francisella cf novicida Fx1,Francisella noatunensis orientalis LADL 07 285A,Francisella noatunensis orientalis Toba 04,Francisella novicida FTE,Francisella novicida FTG,Francisella novicida GA99 3548,Francisella novicida GA99 3549,Francisella novicida U112,Francisella philomiragia ATCC 25015,Francisella philomiragia ATCC 25017,Francisella tularensis 3571,Francisella tularensis 70102010,Francisella tularensis 80700075,Francisella tularensis 80700103,Francisella tularensis 831,Francisella tularensis AS 713,Francisella tularensis FSC033,Francisella tularensis FSC198,Francisella tularensis holarctica 257,Francisella tularensis holarctica F92,Francisella tularensis holarctica FSC022,Francisella tularensis holarctica FSC200,Francisella tularensis holarctica FTNF002 00,Francisella tularensis holarctica LVS,Francisella tularensis holarctica OSU18,Francisella tularensis holarctica URFT1,Francisella tularensis MA00 2987,Francisella tularensis mediasiatica FSC147,Francisella tularensis NE061598,Francisella tularensis SCHU S4,Francisella tularensis TI0902,Francisella tularensis TIGB03,Francisella tularensis WY96 3418,Francisella TX077308,Frateuria aurantia DSM 6220,Gallibacterium anatis UMN179,Glaciecola 4H 3 7 YE 5,Glaciecola agarilytica NO2,Glaciecola arctica BSs20135,Glaciecola chathamensis S18K6,Glaciecola HTCC2999,Glaciecola lipolytica E3,Glaciecola mesophila KMM 241,Glaciecola nitratireducens FR1064,Glaciecola pallidula DSM 14239 ACAM 615,Glaciecola polaris LMG 21857,Glaciecola psychrophila 170,Glaciecola punicea DSM 14233 ACAM 611,Haemophilus parainfluenzae CCUG 13788,Haemophilus aegyptius ATCC 11116,Haemophilus ducreyi 35000HP,Haemophilus haemolyticus HK386,Haemophilus haemolyticus M19107,Haemophilus haemolyticus M19501,Haemophilus haemolyticus M21127,Haemophilus haemolyticus M21621,Haemophilus haemolyticus M21639,Haemophilus influenzae 10810,Haemophilus influenzae 22 1 21,Haemophilus influenzae 3655,Haemophilus influenzae 6P18H1,Haemophilus influenzae 7P49H1,Haemophilus influenzae 86 028NP,Haemophilus influenzae F3031,Haemophilus influenzae F3047,Haemophilus influenzae HK1212,Haemophilus influenzae KR494,Haemophilus influenzae NT127,Haemophilus influenzae PittAA,Haemophilus influenzae PittEE,Haemophilus influenzae PittGG,Haemophilus influenzae PittHH,Haemophilus influenzae PittII,Haemophilus influenzae R2846,Haemophilus influenzae R2866,Haemophilus influenzae R3021,Haemophilus influenzae Rd KW20,Haemophilus influenzae RdAW,Haemophilus oral taxon 851 F0397,Haemophilus parahaemolyticus HK385,Haemophilus parainfluenzae ATCC 33392,Haemophilus parainfluenzae HK2019,Haemophilus parainfluenzae HK262,Haemophilus parainfluenzae T3T1,Haemophilus paraphrohaemolyticus HK411,Haemophilus parasuis 29755,Haemophilus parasuis SH0165,Haemophilus parasuis ZJ0906,Haemophilus pittmaniae HK 85,Haemophilus somnus 129PT,Haemophilus somnus 2336,Haemophilus sputorum HK 2154,Hahella chejuensis KCTC 2396,Halomonas boliviensis LC1,Halomonas elongata DSM 2581,Halomonas GFAJ 1,Halomonas HAL1,Halomonas KM 1,Halomonas TD01,Halomonas titanicae BH1,Halorhodospira halophila SL1,Halothiobacillus neapolitanus c2,Idiomarina A28L,Idiomarina baltica OS145,Idiomarina loihiensis GSL 199,Idiomarina loihiensis L2TR,Idiomarina xiamenensis 10 D 4,Kangiella koreensis DSM 16069,Klebsiella 1 1 55,Klebsiella 4 1 44FAA,Klebsiella MS 92 3,Klebsiella OBRC7,Klebsiella oxytoca 10 5242,Klebsiella oxytoca 10 5243,Klebsiella oxytoca 10 5245,Klebsiella oxytoca 10 5246,Klebsiella oxytoca 10 5250,Klebsiella oxytoca E718,Klebsiella oxytoca KCTC 1686,Klebsiella oxytoca M5al,Klebsiella pneumoniae,Klebsiella pneumoniae 1084,Klebsiella pneumoniae 342,Klebsiella pneumoniae 700603,Klebsiella pneumoniae ATCC BAA 1705,Klebsiella pneumoniae ATCC BAA 2146,Klebsiella pneumoniae CG43,Klebsiella pneumoniae DSM 30104,Klebsiella pneumoniae Ecl8,Klebsiella pneumoniae HS11286,Klebsiella pneumoniae hvKP1,Klebsiella pneumoniae JHCK1,Klebsiella pneumoniae JM45,Klebsiella pneumoniae KCTC 2242,Klebsiella pneumoniae KPNIH1,Klebsiella pneumoniae KPNIH10,Klebsiella pneumoniae KPNIH11,Klebsiella pneumoniae KPNIH12,Klebsiella pneumoniae KPNIH14,Klebsiella pneumoniae KPNIH16,Klebsiella pneumoniae KPNIH17,Klebsiella pneumoniae KPNIH18,Klebsiella pneumoniae KPNIH19,Klebsiella pneumoniae KPNIH2,Klebsiella pneumoniae KPNIH20,Klebsiella pneumoniae KPNIH21,Klebsiella pneumoniae KPNIH22,Klebsiella pneumoniae KPNIH23,Klebsiella pneumoniae KPNIH4,Klebsiella pneumoniae KPNIH5,Klebsiella pneumoniae KPNIH6,Klebsiella pneumoniae KPNIH7,Klebsiella pneumoniae KPNIH8,Klebsiella pneumoniae KPNIH9,Klebsiella pneumoniae KpQ3,Klebsiella pneumoniae MGH 78578,Klebsiella pneumoniae NTUH K2044,Klebsiella pneumoniae rhinoscleromatis ATCC 13884,Klebsiella pneumoniae RYC492,Klebsiella pneumoniae ST258 K26BO,Klebsiella pneumoniae ST258 K28BO,Klebsiella pneumoniae ST512 K30BO,Klebsiella pneumoniae VA360,Klebsiella pneumoniae WGLW1,Klebsiella pneumoniae WGLW2,Klebsiella pneumoniae WGLW3,Klebsiella pneumoniae WGLW5,Klebsiella variicola At 22,Legionella drancourtii LLAP12,Legionella longbeachae D 4968,Legionella longbeachae NSW150,Legionella pneumophila,Legionella pneumophila 2300 99 Alcoy,Legionella pneumophila ATCC 43290,Legionella pneumophila Corby,Legionella pneumophila Lens,Legionella pneumophila Lorraine,Legionella pneumophila Paris,Legionella pneumophila Philadelphia 1,Legionella pneumophila Thunder Bay,Mannheimia haemolytica D153,Mannheimia haemolytica D171,Mannheimia haemolytica D174,Mannheimia haemolytica M42548,Mannheimia haemolytica PHL213,Mannheimia haemolytica serotype 6 H23,Mannheimia haemolytica serotype A2 BOVINE,Mannheimia haemolytica serotype A2 OVINE,Mannheimia haemolytica USDA ARS SAM 185,Mannheimia haemolytica USDA ARS USMARC 183,Mannheimia haemolytica USMARC 2286,Mannheimia succiniciproducens MBEL55E,Marinobacter adhaerens HP15,Marinobacter algicola DG893,Marinobacter aquaeolei VT8,Marinobacter BSs20148,Marinobacter ELB17,Marinobacter hydrocarbonoclasticus ATCC 49840,Marinobacter MnI7 9,Marinobacter santoriniensis NKSG1,Marinomonas MED121,Marinomonas mediterranea MMB 1,Marinomonas MWYL1,Marinomonas posidonica IVIA Po 181,Methylococcus capsulatus Bath,Methylomicrobium album BG8,Methylomicrobium alcaliphilum,Methylomonas methanica MC09,Methylophaga aminisulfidivorans MP,Methylophaga JAM1,Methylophaga JAM7,Methylophaga lonarensis MPL,Methylophaga thiooxidans DMS010,Moraxella catarrhalis 101P30B1,Moraxella catarrhalis 103P14B1,Moraxella catarrhalis 12P80B1,Moraxella catarrhalis 46P47B1,Moraxella catarrhalis 7169,Moraxella catarrhalis BBH18,Moraxella catarrhalis BC1,Moraxella catarrhalis BC7,Moraxella catarrhalis BC8,Moraxella catarrhalis CO72,Moraxella catarrhalis O35E,Moraxella catarrhalis RH4,Moraxella macacae 0408225,Morganella morganii KT,Morganella morganii SC01,Nitrosococcus halophilus Nc4,Nitrosococcus oceani AFC27,Nitrosococcus oceani ATCC 19707,Nitrosococcus watsonii C 113,Oceanimonas GK1,Pantoea aB,Pantoea agglomerans 299R,Pantoea agglomerans IG1,Pantoea ananatis,Pantoea ananatis AJ13355,Pantoea ananatis LMG 20103,Pantoea ananatis PA13,Pantoea At 9b,Pantoea GM01,Pantoea Sc1,Pantoea SL1 M5,Pantoea stewartii DC283,Pantoea vagans C9 1,Pantoea YR343,Pasteurella bettyae CCUG 2042,Pasteurella dagmatis ATCC 43325,Pasteurella multocida 3480,Pasteurella multocida 36950,Pasteurella multocida Anand1 buffalo,Pasteurella multocida Anand1 cattle,Pasteurella multocida Anand1 goat,Pasteurella multocida gallicida Anand1 poultry,Pasteurella multocida gallicida P1059,Pasteurella multocida gallicida X73,Pasteurella multocida HN06,Pasteurella multocida P52VAC,Pasteurella multocida Pm70,Pectobacterium atrosepticum SCRI1043,Pectobacterium carotovorum brasiliensis PBR1692,Pectobacterium carotovorum PC1,Pectobacterium carotovorum PCC21,Pectobacterium carotovorum WPP14,Pectobacterium SCC3193,Pectobacterium wasabiae CFBP 3304,Pectobacterium wasabiae WPP163,Photobacterium AK15,Photobacterium angustum S14,Photobacterium damselae CIP 102761,Photobacterium leiognathi mandapamensis svers 1 1,Photobacterium profundum 3TCK,Photobacterium profundum SS9,Photobacterium SKA34,Photorhabdus asymbiotica ATCC 43949,Photorhabdus luminescens laumondii TTO1,Proteus mirabilis ATCC 29906,Proteus mirabilis BB2000,Proteus mirabilis HI4320,Proteus mirabilis WGLW4,Proteus mirabilis WGLW6,Proteus penneri ATCC 35198,Providencia alcalifaciens Dmel2,Providencia alcalifaciens DSM 30120,Providencia burhodogranariea DSM 19968,Providencia rettgeri Dmel1,Providencia rettgeri DSM 1131,Providencia rustigianii DSM 4541,Providencia sneebia DSM 19967,Providencia stuartii ATCC 25827,Providencia stuartii MRSN 2154,Pseudoalteromonas arctica A 37 1 2,Pseudoalteromonas atlantica T6c,Pseudoalteromonas BSi20311,Pseudoalteromonas BSi20429,Pseudoalteromonas BSi20439,Pseudoalteromonas BSi20480,Pseudoalteromonas BSi20495,Pseudoalteromonas BSi20652,Pseudoalteromonas Bsw20308,Pseudoalteromonas citrea NCIMB 1889,Pseudoalteromonas flavipulchra JG1,Pseudoalteromonas haloplanktis ANT 505,Pseudoalteromonas haloplanktis TAC125,Pseudoalteromonas luteoviolacea B ATCC 29581,Pseudoalteromonas marina mano4,Pseudoalteromonas piscicida JCM 20779,Pseudoalteromonas rubra ATCC 29570,Pseudoalteromonas SM9913,Pseudoalteromonas spongiae UST010723 006,Pseudoalteromonas tunicata D2,Pseudoalteromonas undina NCIMB 2128,Pseudomonas 2 1 26,Pseudomonas aeruginosa 138244,Pseudomonas aeruginosa 152504,Pseudomonas aeruginosa 18A,Pseudomonas aeruginosa 2192,Pseudomonas aeruginosa 39016,Pseudomonas aeruginosa ATCC 14886,Pseudomonas aeruginosa ATCC 25324,Pseudomonas aeruginosa ATCC 700888,Pseudomonas aeruginosa B136 33,Pseudomonas aeruginosa C3719,Pseudomonas aeruginosa CI27,Pseudomonas aeruginosa CIG1,Pseudomonas aeruginosa DK2,Pseudomonas aeruginosa E2,Pseudomonas aeruginosa LES431,Pseudomonas aeruginosa LESB58,Pseudomonas aeruginosa M18,Pseudomonas aeruginosa MPAO1 P1,Pseudomonas aeruginosa MPAO1 P2,Pseudomonas aeruginosa MTB,Pseudomonas aeruginosa NCGM2 S1,Pseudomonas aeruginosa NCMG1179,Pseudomonas aeruginosa PA1,Pseudomonas aeruginosa PA1R,Pseudomonas aeruginosa PA21 ST175,Pseudomonas aeruginosa PA7,Pseudomonas aeruginosa PAb1,Pseudomonas aeruginosa PACS2,Pseudomonas aeruginosa PADK2 CF510,Pseudomonas aeruginosa PAO1,Pseudomonas aeruginosa PAO579,Pseudomonas aeruginosa RP73,Pseudomonas aeruginosa SCV20265,Pseudomonas aeruginosa UCBPP PA14,Pseudomonas aeruginosa VRFPA01,Pseudomonas Ag1,Pseudomonas avellanae BPIC 631,Pseudomonas brassicacearum NFM421,Pseudomonas chlororaphis aureofaciens 30 84,Pseudomonas chlororaphis O6,Pseudomonas Chol1,Pseudomonas denitrificans ATCC 13867,Pseudomonas entomophila L48,Pseudomonas extremaustralis 14 3 substr 14 3b,Pseudomonas fluorescens A506,Pseudomonas fluorescens BBc6R8,Pseudomonas fluorescens BRIP34879,Pseudomonas fluorescens CHA0,Pseudomonas fluorescens F113,Pseudomonas fluorescens Pf0 1,Pseudomonas fluorescens Pf 5,Pseudomonas fluorescens Q2 87,Pseudomonas fluorescens Q8r1 96,Pseudomonas fluorescens SBW25,Pseudomonas fluorescens SS101,Pseudomonas fluorescens WH6,Pseudomonas fragi A22,Pseudomonas fulva 12 X,Pseudomonas fuscovaginae UPB0736,Pseudomonas geniculata N1,Pseudomonas GM102,Pseudomonas GM16,Pseudomonas GM17,Pseudomonas GM18,Pseudomonas GM21,Pseudomonas GM24,Pseudomonas GM25,Pseudomonas GM30,Pseudomonas GM33,Pseudomonas GM41 2012,Pseudomonas GM48,Pseudomonas GM49,Pseudomonas GM50,Pseudomonas GM55,Pseudomonas GM60,Pseudomonas GM67,Pseudomonas GM74,Pseudomonas GM78,Pseudomonas GM79,Pseudomonas GM80,Pseudomonas GM84,Pseudomonas HYS,Pseudomonas M47T1,Pseudomonas mandelii JR 1,Pseudomonas mendocina DLHK,Pseudomonas mendocina NK 01,Pseudomonas mendocina ymp,Pseudomonas monteilii SB3078,Pseudomonas monteilii SB3101,Pseudomonas ND6,Pseudomonas PAMC 25886,Pseudomonas poae RE 1 1 14,Pseudomonas pseudoalcaligenes CECT 5344,Pseudomonas pseudoalcaligenes KF707,Pseudomonas psychrotolerans L19,Pseudomonas putida BIRD 1,Pseudomonas putida DOT T1E,Pseudomonas putida F1,Pseudomonas putida GB 1,Pseudomonas putida H8234,Pseudomonas putida HB3267,Pseudomonas putida KT2440,Pseudomonas putida LS46,Pseudomonas putida NBRC 14164,Pseudomonas putida S11,Pseudomonas putida S16,Pseudomonas putida UW4,Pseudomonas putida W619,Pseudomonas R81,Pseudomonas resinovorans NBRC 106553,Pseudomonas S9,Pseudomonas stutzeri A1501,Pseudomonas stutzeri ATCC 14405 CCUG 16156,Pseudomonas stutzeri ATCC 17588 LMG 11199,Pseudomonas stutzeri CCUG 29243,Pseudomonas stutzeri DSM 10701,Pseudomonas stutzeri DSM 4166,Pseudomonas stutzeri KOS6,Pseudomonas stutzeri NF13,Pseudomonas stutzeri RCH2,Pseudomonas stutzeri TS44,Pseudomonas synxantha BG33R,Pseudomonas syringae 642,Pseudomonas syringae aceris M302273,Pseudomonas syringae actinidiae M302091,Pseudomonas syringae aesculi 0893 23,Pseudomonas syringae aesculi 2250,Pseudomonas syringae aesculi NCPPB3681,Pseudomonas syringae aptata DSM 50252,Pseudomonas syringae avellanae ISPaVe013,Pseudomonas syringae avellanae ISPaVe037,Pseudomonas syringae B64,Pseudomonas syringae B728a,Pseudomonas syringae BRIP34876,Pseudomonas syringae BRIP34881,Pseudomonas syringae BRIP39023,Pseudomonas syringae Cit 7,Pseudomonas syringae FF5,Pseudomonas syringae glycinea B076,Pseudomonas syringae glycinea race 4,Pseudomonas syringae japonica M301072,Pseudomonas syringae lachrymans M301315,Pseudomonas syringae lachrymans M302278,Pseudomonas syringae Lz4W,Pseudomonas syringae maculicola ES4326,Pseudomonas syringae mori 301020,Pseudomonas syringae morsprunorum M302280,Pseudomonas syringae oryzae 1 6,Pseudomonas syringae phaseolicola 1448A,Pseudomonas syringae pisi 1704B,Pseudomonas syringae tabaci ATCC 11528,Pseudomonas syringae tomato DC3000,Pseudomonas syringae tomato K40,Pseudomonas syringae tomato Max13,Pseudomonas syringae tomato NCPPB 1108,Pseudomonas syringae tomato T1,Pseudomonas TJI 51,Pseudomonas TKP,Pseudomonas UK4,Pseudomonas viridiflava UASWS0038,Pseudomonas VLB120,Pseudoxanthomonas spadix BD a59,Pseudoxanthomonas suwonensis 11 1,Psychrobacter 1501 2011,Psychrobacter arcticus 273 4,Psychrobacter cryohalolentis K5,Psychrobacter G,Psychrobacter PAMC 21119,Psychrobacter PRwf 1,Psychromonas CNPT3,Psychromonas ingrahamii 37,Rahnella aquatilis CIP 78 65 ATCC 33071,Rahnella aquatilis HX2,Rahnella Y9602,Raoultella ornithinolytica B6,Rhodanobacter 115,Rhodanobacter 116 2,Rhodanobacter 2APBS1,Rhodanobacter fulvus Jip2,Rhodanobacter spathiphylli B39,Rhodanobacter thiooxydans LCS2,Saccharophagus degradans 2 40,Salmonella bongori NCTC 12419,Salmonella bongori Sbon 167,Salmonella enterica arizonae serovar 62 z4 z23,Salmonella enterica houtenae ATCC BAA 1581,Salmonella enterica salamae 3588 07,Salmonella enterica serovar 4 5 12 i 08 1736,Salmonella enterica serovar 4 5 12 i CVM23701,Salmonella enterica serovar Adelaide A4 669,Salmonella enterica serovar Agona 24249,Salmonella enterica serovar Agona SH08SF124,Salmonella enterica serovar Agona SH10GFN094,Salmonella enterica serovar Agona SH11G1113,Salmonella enterica serovar Agona SL483,Salmonella enterica serovar Alachua R6 377,Salmonella enterica serovar Baildon R6 199,Salmonella enterica serovar Bareilly 06 0784,Salmonella enterica serovar Bareilly CFSAN000189,Salmonella enterica serovar Bovismorbificans 3114,Salmonella enterica serovar Choleraesuis SC B67,Salmonella enterica serovar Choleraesuis SCSA50,Salmonella enterica Serovar Cubana CFSAN002050,Salmonella enterica serovar Dublin CT 02021853,Salmonella enterica serovar Dublin HWS51,Salmonella enterica serovar Dublin SD3246,Salmonella enterica serovar Dublin SL1438,Salmonella enterica serovar Dublin UC16,Salmonella enterica serovar Enteritidis 08 0047,Salmonella enterica serovar Enteritidis 08 0128,Salmonella enterica serovar Enteritidis 08 0627,Salmonella enterica serovar Enteritidis 13183 1,Salmonella enterica serovar Enteritidis 13 1,Salmonella enterica serovar Enteritidis 17927,Salmonella enterica serovar Enteritidis 18569,Salmonella enterica serovar Enteritidis 20037,Salmonella enterica serovar Enteritidis 2010 1237,Salmonella enterica serovar Enteritidis 2010K 0669,Salmonella enterica serovar Enteritidis 2010K 2599,Salmonella enterica serovar Enteritidis 22510 1,Salmonella enterica serovar Enteritidis 22558,Salmonella enterica serovar Enteritidis 22704,Salmonella enterica serovar Enteritidis 33944,Salmonella enterica serovar Enteritidis 436,Salmonella enterica serovar Enteritidis 485549 17,Salmonella enterica serovar Enteritidis 50 3079,Salmonella enterica serovar Enteritidis 50 5646,Salmonella enterica serovar Enteritidis 53 407,Salmonella enterica serovar Enteritidis 543463 22 17,Salmonella enterica serovar Enteritidis 543463 40 18,Salmonella enterica serovar Enteritidis 543463 42 20,Salmonella enterica serovar Enteritidis 561362 1 1,Salmonella enterica serovar Enteritidis 561362 9 7,Salmonella enterica serovar Enteritidis 576709,Salmonella enterica serovar Enteritidis 58 6482,Salmonella enterica serovar Enteritidis 596866 22,Salmonella enterica serovar Enteritidis 596866 70,Salmonella enterica serovar Enteritidis 607307 2,Salmonella enterica serovar Enteritidis 607307 6,Salmonella enterica serovar Enteritidis 607308 16,Salmonella enterica serovar Enteritidis 607308 19,Salmonella enterica serovar Enteritidis 607308 9,Salmonella enterica serovar Enteritidis 622731 39,Salmonella enterica serovar Enteritidis 629163,Salmonella enterica serovar Enteritidis 629164 26,Salmonella enterica serovar Enteritidis 629164 37,Salmonella enterica serovar Enteritidis 62 1976,Salmonella enterica serovar Enteritidis 635290 58,Salmonella enterica serovar Enteritidis 638970 15,Salmonella enterica serovar Enteritidis 639016 6,Salmonella enterica serovar Enteritidis 639672 46,Salmonella enterica serovar Enteritidis 639672 50,Salmonella enterica serovar Enteritidis 640631,Salmonella enterica serovar Enteritidis 642044 4 1,Salmonella enterica serovar Enteritidis 642044 8 1,Salmonella enterica serovar Enteritidis 642046 4 7,Salmonella enterica serovar Enteritidis 648898 4 5,Salmonella enterica serovar Enteritidis 648899 3 17,Salmonella enterica serovar Enteritidis 648900 1 16,Salmonella enterica serovar Enteritidis 648901 16 16,Salmonella enterica serovar Enteritidis 648901 1 17,Salmonella enterica serovar Enteritidis 648901 39 2,Salmonella enterica serovar Enteritidis 648901 6 18,Salmonella enterica serovar Enteritidis 648902 6 8,Salmonella enterica serovar Enteritidis 648903 1 6,Salmonella enterica serovar Enteritidis 648904 3 6,Salmonella enterica serovar Enteritidis 648905 5 18,Salmonella enterica serovar Enteritidis 653049 13 19,Salmonella enterica serovar Enteritidis 6 0562 1,Salmonella enterica serovar Enteritidis 76 2651,Salmonella enterica serovar Enteritidis 77 0424,Salmonella enterica serovar Enteritidis 77 1427,Salmonella enterica serovar Enteritidis 77 2659,Salmonella enterica serovar Enteritidis 78 1757,Salmonella enterica serovar Enteritidis 81 2625,Salmonella enterica serovar Enteritidis 8b 1,Salmonella enterica serovar Enteritidis CDC 2010K 0895,Salmonella enterica serovar Enteritidis CDC 2010K 0899,Salmonella enterica serovar Enteritidis CDC 2010K 0956,Salmonella enterica serovar Enteritidis CDC 2010K 0968,Salmonella enterica serovar Enteritidis CDC 2010K 1010,Salmonella enterica serovar Enteritidis CDC 2010K 1018,Salmonella enterica serovar Enteritidis CDC 2010K 1441,Salmonella enterica serovar Enteritidis CDC 2010K 1444,Salmonella enterica serovar Enteritidis CDC 2010K 1445,Salmonella enterica serovar Enteritidis CDC 2010K 1455,Salmonella enterica serovar Enteritidis CDC 2010K 1457,Salmonella enterica serovar Enteritidis CDC 2010K 1543,Salmonella enterica serovar Enteritidis CDC 2010K 1558,Salmonella enterica serovar Enteritidis CDC 2010K 1559,Salmonella enterica serovar Enteritidis CDC 2010K 1565,Salmonella enterica serovar Enteritidis CDC 2010K 1566,Salmonella enterica serovar Enteritidis CDC 2010K 1575,Salmonella enterica serovar Enteritidis CDC 2010K 1580,Salmonella enterica serovar Enteritidis CDC 2010K 1594,Salmonella enterica serovar Enteritidis CDC 2010K 1725,Salmonella enterica serovar Enteritidis CDC 2010K 1729,Salmonella enterica serovar Enteritidis CDC 2010K 1745,Salmonella enterica serovar Enteritidis CDC 2010K 1747,Salmonella enterica serovar Enteritidis CDC 2010K 1791,Salmonella enterica serovar Enteritidis CDC 2010K 1795,Salmonella enterica serovar Enteritidis CDC 2010K 1808,Salmonella enterica serovar Enteritidis CDC 2010K 1810,Salmonella enterica serovar Enteritidis CDC 2010K 1811,Salmonella enterica serovar Enteritidis CDC 2010K 1882,Salmonella enterica serovar Enteritidis CDC 2010K 1884,Salmonella enterica serovar Enteritidis CHS4,Salmonella enterica serovar Enteritidis CHS44,Salmonella enterica serovar Enteritidis CVM 56 3991,Salmonella enterica serovar Enteritidis CVM 69 4941,Salmonella enterica serovar Enteritidis CVM 76 3618,Salmonella enterica serovar Enteritidis CVM 81 2490,Salmonella enterica serovar Enteritidis CVM N202,Salmonella enterica serovar Enteritidis P125109,Salmonella enterica serovar Enteritidis PT23,Salmonella enterica serovar Enteritidis SARB17,Salmonella enterica serovar Enteritidis SE10,Salmonella enterica serovar Enteritidis SE15 1,Salmonella enterica serovar Enteritidis SE30663,Salmonella enterica serovar Enteritidis SE8a,Salmonella enterica serovar Enteritidis SL909,Salmonella enterica serovar Enteritidis SL913,Salmonella enterica serovar Gallinarum 287 91,Salmonella enterica serovar Gallinarum 9184,Salmonella enterica serovar Gallinarum Pullorum CDC1983 67,Salmonella enterica serovar Gallinarum pullorum RKS5078,Salmonella enterica serovar Gallinarum SG9,Salmonella enterica serovar Gaminara A4 567,Salmonella enterica serovar Give S5 487,Salmonella enterica serovar Hadar 2011K 0112,Salmonella enterica serovar Hadar RI 05P066,Salmonella enterica serovar Hartford 06 0676,Salmonella enterica serovar Heidelberg 41563,Salmonella enterica serovar Heidelberg 41565,Salmonella enterica serovar Heidelberg 41566,Salmonella enterica serovar Heidelberg 41573,Salmonella enterica serovar Heidelberg 41578,Salmonella enterica serovar Heidelberg 41579,Salmonella enterica serovar Heidelberg B182,Salmonella enterica Serovar Heidelberg CFSAN002069,Salmonella enterica serovar Heidelberg CFSAN00322,Salmonella enterica serovar Heidelberg CFSAN00325,Salmonella enterica serovar Heidelberg CFSAN00326,Salmonella enterica serovar Heidelberg CFSAN00328,Salmonella enterica serovar Heidelberg SL476,Salmonella enterica serovar Heidelberg SL486,Salmonella enterica serovar Hvittingfoss A4 620,Salmonella enterica serovar Infantis SARB27,Salmonella enterica serovar Inverness R8 3668,Salmonella enterica serovar Javiana CFSAN001992,Salmonella enterica serovar Javiana GA MM04042433,Salmonella enterica serovar Johannesburg S5 703,Salmonella enterica serovar Kentucky CDC 191,Salmonella enterica serovar Kentucky CVM29188,Salmonella enterica serovar Minnesota A4 603,Salmonella enterica serovar Mississippi 2010K 1406,Salmonella enterica serovar Mississippi A4 633,Salmonella enterica serovar Montevideo 19N,Salmonella enterica serovar Montevideo 2009083312,Salmonella enterica serovar Montevideo 2009085258,Salmonella enterica serovar Montevideo 29N,Salmonella enterica serovar Montevideo 315731156,Salmonella enterica serovar Montevideo 315996572,Salmonella enterica serovar Montevideo 366867,Salmonella enterica serovar Montevideo 413180,Salmonella enterica serovar Montevideo 414877,Salmonella enterica serovar Montevideo 42N,Salmonella enterica serovar Montevideo 4441 H,Salmonella enterica serovar Montevideo 446600,Salmonella enterica serovar Montevideo 495297 1,Salmonella enterica serovar Montevideo 495297 3,Salmonella enterica serovar Montevideo 495297 4,Salmonella enterica serovar Montevideo 507440 20,Salmonella enterica serovar Montevideo 515920 1,Salmonella enterica serovar Montevideo 515920 2,Salmonella enterica serovar Montevideo 531954,Salmonella enterica serovar Montevideo 556150 1,Salmonella enterica serovar Montevideo 556152,Salmonella enterica serovar Montevideo 609458 1,Salmonella enterica serovar Montevideo 609460,Salmonella enterica serovar Montevideo 80959 06,Salmonella enterica serovar Montevideo 81038 01,Salmonella enterica serovar Montevideo ATCC BAA710,Salmonella enterica serovar Montevideo CASC 09SCPH15965,Salmonella enterica serovar Montevideo CT 02035278,Salmonella enterica serovar Montevideo CT 02035318,Salmonella enterica serovar Montevideo CT 02035320,Salmonella enterica serovar Montevideo CT 02035321,Salmonella enterica serovar Montevideo CT 02035327,Salmonella enterica serovar Montevideo IA 2009159199,Salmonella enterica serovar Montevideo IA 2010008282,Salmonella enterica serovar Montevideo IA 2010008283,Salmonella enterica serovar Montevideo IA 2010008284,Salmonella enterica serovar Montevideo IA 2010008285,Salmonella enterica serovar Montevideo IA 2010008286,Salmonella enterica serovar Montevideo IA 2010008287,Salmonella enterica serovar Montevideo LQC 10,Salmonella enterica serovar Montevideo MB101509 0077,Salmonella enterica serovar Montevideo MB102109 0047,Salmonella enterica serovar Montevideo MB110209 0055,Salmonella enterica serovar Montevideo MB111609 0052,Salmonella enterica serovar Montevideo MD MDA09249507,Salmonella enterica serovar Montevideo NC MB110209 0054,Salmonella enterica serovar Montevideo OH 2009072675,Salmonella enterica serovar Montevideo S5 403,Salmonella enterica serovar Montevideo SARB30,Salmonella enterica serovar Montevideo SARB31,Salmonella enterica serovar Newport CVM 19443,Salmonella enterica serovar Newport CVM 19447,Salmonella enterica serovar Newport CVM 19449,Salmonella enterica serovar Newport CVM 19470,Salmonella enterica serovar Newport CVM 19536,Salmonella enterica serovar Newport CVM 19567,Salmonella enterica serovar Newport CVM 19593,Salmonella enterica serovar Newport CVM 21538,Salmonella enterica serovar Newport CVM 21539,Salmonella enterica serovar Newport CVM 21550,Salmonella enterica serovar Newport CVM 21554,Salmonella enterica serovar Newport CVM 21559,Salmonella enterica serovar Newport CVM 22425,Salmonella enterica serovar Newport CVM 22462,Salmonella enterica serovar Newport CVM 22513,Salmonella enterica serovar Newport CVM 33953,Salmonella enterica serovar Newport CVM 35185,Salmonella enterica serovar Newport CVM 35188,Salmonella enterica serovar Newport CVM 35199,Salmonella enterica serovar Newport CVM 35202,Salmonella enterica serovar Newport CVM 37978,Salmonella enterica serovar Newport CVM 4176,Salmonella enterica serovar Newport CVM N1543,Salmonella enterica serovar Newport CVM N18486,Salmonella enterica serovar Newport Henan 3,Salmonella enterica serovar Newport JS09102,Salmonella enterica serovar Newport Levine 1,Salmonella enterica serovar Newport Levine 15,Salmonella enterica serovar Newport SH111077,Salmonella enterica serovar Newport Shandong 3,Salmonella enterica serovar Newport SL254,Salmonella enterica serovar Newport SL317,Salmonella enterica serovar Newport USMARC S3124 1,Salmonella enterica serovar Paratyphi A AKU 12601,Salmonella enterica serovar Paratyphi A ATCC 9150,Salmonella enterica serovar Paratyphi B SPB7,Salmonella enterica serovar Paratyphi C RKS4594,Salmonella enterica serovar Pomona ATCC 10729,Salmonella enterica serovar Pullorum ATCC 9120,Salmonella enterica serovar Pullorum S06004,Salmonella enterica serovar Rubislaw A4 653,Salmonella enterica serovar Saintpaul SARA23,Salmonella enterica serovar Saintpaul SARA29,Salmonella enterica serovar Schwarzengrund CVM19633,Salmonella enterica serovar Schwarzengrund SL480,Salmonella enterica serovar Senftenberg A4 543,Salmonella enterica serovar Senftenberg SS209,Salmonella enterica serovar Tennessee CDC07 0191,Salmonella enterica serovar Thompson RM6836,Salmonella enterica serovar Typhi BL196,Salmonella enterica serovar Typhi CR0044,Salmonella enterica serovar Typhi CT18,Salmonella enterica serovar Typhi E00 7866,Salmonella enterica serovar Typhi E02 1180,Salmonella enterica serovar Typhi E98 2068,Salmonella enterica serovar Typhi E98 3139,Salmonella enterica serovar Typhi J185,Salmonella enterica serovar Typhi M223,Salmonella enterica serovar Typhi P stx 12,Salmonella enterica serovar Typhi ST0208,Salmonella enterica serovar Typhi Ty2,Salmonella enterica serovar Typhi Ty21a,Salmonella enterica serovar Typhi UJ308A,Salmonella enterica serovar Typhi UJ816A,Salmonella enterica serovar Typhimurium 14028S,Salmonella enterica serovar Typhimurium 798,Salmonella enterica serovar Typhimurium D23580,Salmonella enterica serovar Typhimurium DT2,Salmonella enterica serovar Typhimurium LT2,Salmonella enterica serovar Typhimurium SL1344,Salmonella enterica serovar Typhimurium ST1660 06,Salmonella enterica serovar Typhimurium ST4 74,Salmonella enterica serovar Typhimurium STm1,Salmonella enterica serovar Typhimurium STm10,Salmonella enterica serovar Typhimurium STm11,Salmonella enterica serovar Typhimurium STm12,Salmonella enterica serovar Typhimurium STm2,Salmonella enterica serovar Typhimurium STm3,Salmonella enterica serovar Typhimurium STm4,Salmonella enterica serovar Typhimurium STm5,Salmonella enterica serovar Typhimurium STm6,Salmonella enterica serovar Typhimurium STm8,Salmonella enterica serovar Typhimurium STm9,Salmonella enterica serovar Typhimurium T000240,Salmonella enterica serovar Typhimurium TN061786,Salmonella enterica serovar Typhimurium U288,Salmonella enterica serovar Typhimurium UK 1,Salmonella enterica Serovar Typhimurium var 5 CFSAN001921,Salmonella enterica serovar Uganda R8 3404,Salmonella enterica serovar Urbana R8 2977,Salmonella enterica serovar Virchow SL491,Salmonella enterica serovar Wandsworth A4 580,Salmonella enterica serovar Weltevreden 2007 60 3289 1,Salmonella enterica serovar Weltevreden HI N05 537,Salmonella typhimurium DT104,Serratia AS12,Serratia AS13,Serratia ATCC 39006,Serratia liquefaciens ATCC 27592,Serratia M24T3,Serratia marcescens FGI94,Serratia marcescens VGH107,Serratia marcescens WW4,Serratia odorifera 4Rx13,Serratia odorifera DSM 4582,Serratia plymuthica A30,Serratia plymuthica AS9,Serratia plymuthica PRI 2C,Serratia plymuthica S13,Serratia proteamaculans 568,Serratia symbiotica Cinara cedri,Serratia symbiotica Tucson,Shewanella amazonensis SB2B,Shewanella ANA 3,Shewanella baltica BA175,Shewanella baltica OS117,Shewanella baltica OS155,Shewanella baltica OS183,Shewanella baltica OS185,Shewanella baltica OS195,Shewanella baltica OS223,Shewanella baltica OS625,Shewanella baltica OS678,Shewanella benthica KT99,Shewanella denitrificans OS217,Shewanella frigidimarina NCIMB 400,Shewanella halifaxensis HAW EB4,Shewanella HN 41,Shewanella loihica PV 4,Shewanella MR 4,Shewanella MR 7,Shewanella oneidensis MR 1,Shewanella pealeana ATCC 700345,Shewanella piezotolerans WP3,Shewanella putrefaciens 200,Shewanella putrefaciens CN 32,Shewanella sediminis HAW EB3,Shewanella violacea DSS12,Shewanella W3 18 1,Shewanella woodyi ATCC 51908,Shigella boydii 3594 74,Shigella boydii 4444 74,Shigella boydii 5216 82,Shigella boydii 965 58,Shigella boydii ATCC 9905,Shigella boydii CDC 3083 94,Shigella boydii Sb227,Shigella D9,Shigella dysenteriae 1012,Shigella dysenteriae 155 74,Shigella dysenteriae 1617,Shigella dysenteriae 225 75,Shigella dysenteriae CDC 74 1112,Shigella dysenteriae Sd197,Shigella flexneri 1235 66,Shigella flexneri 1485 80,Shigella flexneri 2002017,Shigella flexneri 2747 71,Shigella flexneri 2850 71,Shigella flexneri 2930 71,Shigella flexneri 2a 2457T,Shigella flexneri 2a 301,Shigella flexneri 4343 70,Shigella flexneri 5 8401,Shigella flexneri 5a M90T,Shigella flexneri 6603 63,Shigella flexneri CCH060,Shigella flexneri CDC 796 83,Shigella flexneri J1713,Shigella flexneri K 1770,Shigella flexneri K 218,Shigella flexneri K 227,Shigella flexneri K 272,Shigella flexneri K 304,Shigella flexneri K 315,Shigella flexneri K 404,Shigella flexneri K 671,Shigella flexneri VA 6,Shigella sonnei 3226 85,Shigella sonnei 3233 85,Shigella sonnei 4822 66,Shigella sonnei 53G,Shigella sonnei Moseley,Shigella sonnei Ss046,Simiduia agarivorans SA1,Sodalis glossinidius morsitans,Stenotrophomonas maltophilia Ab55555,Stenotrophomonas maltophilia AU12 09,Stenotrophomonas maltophilia D457,Stenotrophomonas maltophilia EPM1,Stenotrophomonas maltophilia JV3,Stenotrophomonas maltophilia K279a,Stenotrophomonas maltophilia R551 3,Stenotrophomonas SKA14,Teredinibacter turnerae T7901,Thalassolituus oleivorans MIL 1,Thioalkalimicrobium aerophilum AL3,Thioalkalimicrobium cyclicum ALM1,Thioalkalivibrio K90mix,Thioalkalivibrio nitratireducens DSM 14787,Thioalkalivibrio sulfidophilus HL EbGr7,Thioalkalivibrio thiocyanoxidans ARh 4,Thiocystis violascens DSM 198,Thioflavicoccus mobilis 8321,Thiomicrospira crunogena XCL 2,Tolumonas auensis DSM 9187,Vibrio alginolyticus 12G01,Vibrio alginolyticus 40B,Vibrio alginolyticus E0666,Vibrio alginolyticus NBRC 15630 ATCC 17749,Vibrio AND4,Vibrio anguillarum 775,Vibrio brasiliensis LMG 20546,Vibrio campbellii CAIM 519,Vibrio campbellii DS40M4,Vibrio caribbenthicus ATCC BAA 2122,Vibrio cholerae,Vibrio cholerae 12129 1,Vibrio cholerae 1587,Vibrio cholerae 2740 80,Vibrio cholerae 4260B,Vibrio cholerae 623 39,Vibrio cholerae AM 19226,Vibrio cholerae B33,Vibrio cholerae BJG 01,Vibrio cholerae bv albensis VL426,Vibrio cholerae BX 330286,Vibrio cholerae CIRS101,Vibrio cholerae CP1030 3,Vibrio cholerae CP1032 5,Vibrio cholerae CP1033 6,Vibrio cholerae CP1035 8,Vibrio cholerae CP1037 10,Vibrio cholerae CP1038 11,Vibrio cholerae CP1040 13,Vibrio cholerae CP1041 14,Vibrio cholerae CP1042 15,Vibrio cholerae CP1044 17,Vibrio cholerae CP1046 19,Vibrio cholerae CP1047 20,Vibrio cholerae CP1048 21,Vibrio cholerae CP1050 23,Vibrio cholerae CT 5369 93,Vibrio cholerae H1,Vibrio cholerae HC 02A1,Vibrio cholerae HC 02C1,Vibrio cholerae HC 06A1,Vibrio cholerae HC 17A1,Vibrio cholerae HC 17A2,Vibrio cholerae HC 19A1,Vibrio cholerae HC 1A2,Vibrio cholerae HC 20A2,Vibrio cholerae HC 21A1,Vibrio cholerae HC 22A1,Vibrio cholerae HC 23A1,Vibrio cholerae HC 28A1,Vibrio cholerae HC 32A1,Vibrio cholerae HC 33A2,Vibrio cholerae HC 37A1,Vibrio cholerae HC 38A1,Vibrio cholerae HC 39A1,Vibrio cholerae HC 40A1,Vibrio cholerae HC 41A1,Vibrio cholerae HC 41B1,Vibrio cholerae HC 42A1,Vibrio cholerae HC 43A1,Vibrio cholerae HC 43B1,Vibrio cholerae HC 44C1,Vibrio cholerae HC 46A1,Vibrio cholerae HC 46B1,Vibrio cholerae HC 47A1,Vibrio cholerae HC 48A1,Vibrio cholerae HC 48B2,Vibrio cholerae HC 49A2,Vibrio cholerae HC 50A1,Vibrio cholerae HC 50A2,Vibrio cholerae HC 51A1,Vibrio cholerae HC 52A1,Vibrio cholerae HC 55A1,Vibrio cholerae HC 55B2,Vibrio cholerae HC 55C2,Vibrio cholerae HC 56A1,Vibrio cholerae HC 56A2,Vibrio cholerae HC 57A1,Vibrio cholerae HC 57A2,Vibrio cholerae HC 59A1,Vibrio cholerae HC 59B1,Vibrio cholerae HC 60A1,Vibrio cholerae HC 61A1,Vibrio cholerae HC 61A2,Vibrio cholerae HC 62A1,Vibrio cholerae HC 62B1,Vibrio cholerae HC 64A1,Vibrio cholerae HC 65A1,Vibrio cholerae HC 67A1,Vibrio cholerae HC 68A1,Vibrio cholerae HC 69A1,Vibrio cholerae HC 70A1,Vibrio cholerae HC 71A1,Vibrio cholerae HC 72A2,Vibrio cholerae HC 77A1,Vibrio cholerae HC 78A1,Vibrio cholerae HC 7A1,Vibrio cholerae HC 80A1,Vibrio cholerae HC 81A1,Vibrio cholerae HC 81A2,Vibrio cholerae HCUF01,Vibrio cholerae HE39,Vibrio cholerae HE48,Vibrio cholerae HE 09,Vibrio cholerae HE 16,Vibrio cholerae HE 25,Vibrio cholerae HE 40,Vibrio cholerae HE 45,Vibrio cholerae HE 46,Vibrio cholerae HENC 01,Vibrio cholerae HENC 02,Vibrio cholerae HENC 03,Vibrio cholerae HFU 02,Vibrio cholerae IEC224,Vibrio cholerae INDRE 91 1,Vibrio cholerae LMA3984 4,Vibrio cholerae M66 2,Vibrio cholerae MAK 757,Vibrio cholerae MJ 1236,Vibrio cholerae MO10,Vibrio cholerae MZO 2,Vibrio cholerae MZO 3,Vibrio cholerae NCTC 8457,Vibrio cholerae O1 116059,Vibrio cholerae O1 116063,Vibrio cholerae O1 2010EL 1786,Vibrio cholerae O1 2010EL 1792,Vibrio cholerae O1 2010EL 1798,Vibrio cholerae O1 87395,Vibrio cholerae O1 95412,Vibrio cholerae O1 AG 7404,Vibrio cholerae O1 AG 8040,Vibrio cholerae O1 Amazonia,Vibrio cholerae O1 biovar El Tor N16961,Vibrio cholerae O1 EC 0009,Vibrio cholerae O1 EC 0012,Vibrio cholerae O1 EC 0027,Vibrio cholerae O1 EC 0051,Vibrio cholerae O1 EDC 020,Vibrio cholerae O1 EDC 022,Vibrio cholerae O1 EM 1536,Vibrio cholerae O1 EM 1546,Vibrio cholerae O1 EM 1626,Vibrio cholerae O1 EM 1676A,Vibrio cholerae O1 EM 1727,Vibrio cholerae O1 Inaba G4222,Vibrio cholerae O1 Nep 21106,Vibrio cholerae O1 Nep 21113,Vibrio cholerae O1 NHCC 004A,Vibrio cholerae O1 NHCC 006C,Vibrio cholerae O1 NHCC 008D,Vibrio cholerae O1 NHCC 010F,Vibrio cholerae O1 PCS 023,Vibrio cholerae O395,Vibrio cholerae PS15,Vibrio cholerae RC27,Vibrio cholerae RC385,Vibrio cholerae RC9,Vibrio cholerae TM 11079 80,Vibrio cholerae TMA 21,Vibrio cholerae V51,Vibrio cholerae V52,Vibrio cholerae VC1761,Vibrio cholerae VC35,Vibrio cholerae VC4370,Vibrio coralliilyticus ATCC BAA 450,Vibrio cyclitrophicus ZF14,Vibrio EJY3,Vibrio Ex25,Vibrio fischeri ES114,Vibrio fischeri MJ11,Vibrio fischeri SR5,Vibrio furnissii CIP 102972,Vibrio furnissii NCTC 11218,Vibrio harveyi 1DA3,Vibrio harveyi ATCC BAA 1116,Vibrio harveyi CAIM 1792,Vibrio harveyi HY01,Vibrio ichthyoenteri ATCC 700023,Vibrio MED222,Vibrio metschnikovii CIP 69 14,Vibrio mimicus CAIM 602,Vibrio mimicus MB 451,Vibrio mimicus SX 4,Vibrio mimicus VM223,Vibrio mimicus VM573,Vibrio mimicus VM603,Vibrio N418,Vibrio nigripulchritudo ATCC 27043,Vibrio nigripulchritudo SnF1,Vibrio ordalii ATCC 33509,Vibrio orientalis CIP 102891,Vibrio orientalis CIP 102891 ATCC 33934,Vibrio parahaemolyticus 10329,Vibrio parahaemolyticus 16,Vibrio parahaemolyticus AN 5034,Vibrio parahaemolyticus AQ3810,Vibrio parahaemolyticus AQ4037,Vibrio parahaemolyticus BB22OP,Vibrio parahaemolyticus K5030,Vibrio parahaemolyticus O1 K33 CDC K4557,Vibrio parahaemolyticus Peru 466,Vibrio parahaemolyticus RIMD 2210633,Vibrio RC341,Vibrio RC586,Vibrio rotiferianus DAT722,Vibrio scophthalmi LMG 19158,Vibrio shilonii AK1,Vibrio sinaloensis DSM 21326,Vibrio splendidus 12B01,Vibrio splendidus ATCC 33789,Vibrio splendidus LGP32,Vibrio tubiashii ATCC 19109,Vibrio tubiashii NCIMB 1337 ATCC 19106,Vibrio vulnificus CMCP6,Vibrio vulnificus MO6 24 O,Vibrio vulnificus YJ016,Wigglesworthia glossinidia endosymbiont of Glossina brevipalpis,Wigglesworthia glossinidia endosymbiont of Glossina morsitans Yale colony,Xanthomonas albilineans GPE PC73,Xanthomonas axonopodis citri 306,Xanthomonas axonopodis citrumelo F1,Xanthomonas axonopodis malvacearum GSPB1386,Xanthomonas axonopodis malvacearum GSPB2388,Xanthomonas axonopodis punicae LMG 859,Xanthomonas axonopodis Xac29 1,Xanthomonas campestris,Xanthomonas campestris 8004,Xanthomonas campestris ATCC 33913,Xanthomonas campestris musacearum NCPPB4381,Xanthomonas campestris raphani 756C,Xanthomonas campestris vasculorum NCPPB702,Xanthomonas campestris vesicatoria 85 10,Xanthomonas citri Aw12879,Xanthomonas citri mangiferaeindicae LMG 941,Xanthomonas fuscans aurantifolii ICPB 10535,Xanthomonas fuscans aurantifolii ICPB 11122,Xanthomonas gardneri ATCC 19865,Xanthomonas oryzae KACC 10331,Xanthomonas oryzae MAFF 311018,Xanthomonas oryzae oryzicola BLS256,Xanthomonas oryzae PXO99A,Xanthomonas perforans 91 118,Xanthomonas sacchari NCPPB 4393,Xanthomonas translucens DAR61454,Xanthomonas translucens DSM 18974,Xanthomonas vesicatoria ATCC 35937,Xenorhabdus bovienii SS 2004,Xenorhabdus nematophila ATCC 19061,Xylella fastidiosa 9a5c,Xylella fastidiosa Dixon,Xylella fastidiosa EB92 1,Xylella fastidiosa GB514,Xylella fastidiosa M12,Xylella fastidiosa M23,Xylella fastidiosa Temecula1,Yersinia aldovae ATCC 35236,Yersinia bercovieri ATCC 43970,Yersinia enterocolitica 8081,Yersinia enterocolitica IP 10393,Yersinia enterocolitica palearctica 105 5R r,Yersinia enterocolitica palearctica PhRBD Ye1,Yersinia enterocolitica palearctica Y11,Yersinia enterocolitica WA 314,Yersinia frederiksenii ATCC 33641,Yersinia intermedia ATCC 29909,Yersinia kristensenii ATCC 33638,Yersinia mollaretii ATCC 43969,Yersinia pestis A1122,Yersinia pestis Angola,Yersinia pestis Antiqua,Yersinia pestis biovar Antiqua B42003004,Yersinia pestis biovar Antiqua E1979001,Yersinia pestis biovar Antiqua UG05 0454,Yersinia pestis biovar Mediaevalis K1973002,Yersinia pestis biovar Medievalis Harbin 35,Yersinia pestis biovar Microtus 91001,Yersinia pestis biovar Orientalis F1991016,Yersinia pestis biovar Orientalis India 195,Yersinia pestis biovar Orientalis IP275,Yersinia pestis biovar Orientalis MG05 1020,Yersinia pestis biovar Orientalis PEXU2,Yersinia pestis CA88 4125,Yersinia pestis CO92,Yersinia pestis D106004,Yersinia pestis D182038,Yersinia pestis FV 1,Yersinia pestis INS,Yersinia pestis KIM 10,Yersinia pestis KIM D27,Yersinia pestis Nepal516,Yersinia pestis Pestoides A,Yersinia pestis Pestoides F,Yersinia pestis PY 01,Yersinia pestis PY 02,Yersinia pestis PY 03,Yersinia pestis PY 04,Yersinia pestis PY 05,Yersinia pestis PY 06,Yersinia pestis PY 07,Yersinia pestis PY 08,Yersinia pestis PY 09,Yersinia pestis PY 10,Yersinia pestis PY 100,Yersinia pestis PY 101,Yersinia pestis PY 102,Yersinia pestis PY 103,Yersinia pestis PY 11,Yersinia pestis PY 113,Yersinia pestis PY 12,Yersinia pestis PY 13,Yersinia pestis PY 14,Yersinia pestis PY 15,Yersinia pestis PY 16,Yersinia pestis PY 19,Yersinia pestis PY 25,Yersinia pestis PY 29,Yersinia pestis PY 32,Yersinia pestis PY 34,Yersinia pestis PY 36,Yersinia pestis PY 42,Yersinia pestis PY 45,Yersinia pestis PY 46,Yersinia pestis PY 47,Yersinia pestis PY 48,Yersinia pestis PY 52,Yersinia pestis PY 53,Yersinia pestis PY 54,Yersinia pestis PY 55,Yersinia pestis PY 56,Yersinia pestis PY 58,Yersinia pestis PY 59,Yersinia pestis PY 60,Yersinia pestis PY 61,Yersinia pestis PY 63,Yersinia pestis PY 64,Yersinia pestis PY 65,Yersinia pestis PY 66,Yersinia pestis PY 71,Yersinia pestis PY 72,Yersinia pestis PY 76,Yersinia pestis PY 88,Yersinia pestis PY 89,Yersinia pestis PY 90,Yersinia pestis PY 91,Yersinia pestis PY 92,Yersinia pestis PY 93,Yersinia pestis PY 94,Yersinia pestis PY 95,Yersinia pestis PY 96,Yersinia pestis PY 98,Yersinia pestis PY 99,Yersinia pestis Z176003,Yersinia pseudotuberculosis IP 31758,Yersinia pseudotuberculosis IP 32953,Yersinia pseudotuberculosis PB1,Yersinia pseudotuberculosis YPIII,Yersinia rohdei ATCC 43380,Yersinia ruckeri ATCC 29473,Beggiatoa alba B18LD,Beggiatoa SS,Bermanella marisrubri,Brenneria EniD312,Cardiobacterium hominis ATCC 15826,Cardiobacterium valvarum F0432,Congregibacter litoralis KT71,Diplorickettsia massiliensis 20B,Ectothiorhodospira PHS 1,Ectothiorhodospiraceae bacterium M19 40,Endoriftia persephone Hot96 1 Hot96 2,Enhydrobacter aerosaccus SK60,Enterovibrio AK16,Fluoribacter dumoffii Tex KL,Gallaecimonas xiamenensis 3 C 1,Grimontia hollisae CIP 101886,Hafnia alvei ATCC 51873,Hydrocarboniphaga effusa AP103,Marichromatium purpuratum 984,Marinobacterium stanieri S30,Methylobacter tundripaludum SV96,Moritella PE36,Neptuniibacter caesariensis,Nitrococcus mobilis Nb 231,Reinekea blandensis MED297,Rheinheimera A13L,Rheinheimera nanhaiensis E407 8,Rickettsiella grylli,Salinisphaera shabanensis E1L3A,Spiribacter UAH SP71,Succinatimonas hippei YIT 12066,Succinivibrionaceae bacterium WG 1,Thiocapsa marina 5811,Thiorhodococcus drewsii AZ1,Thiorhodospira sibirica ATCC 700588,Thiothrix nivea DSM 5205,Vibrionales bacterium SWAT 3,Wohlfahrtiimonas chitiniclastica SH04,Yokenella regensburgei ATCC 43003,Plautia stali symbiont,gamma proteobacterium BDW918,gamma proteobacterium HIMB30,gamma proteobacterium HIMB55,gamma proteobacterium HTCC2207,gamma proteobacterium HTCC5015,gamma proteobacterium IMCC1989,gamma proteobacterium IMCC2047,gamma proteobacterium IMCC3088,gamma proteobacterium NOR51 B,gamma proteobacterium NOR5 3,gamma proteobacterium SCGC AAA001 B15,gamma proteobacterium SCGC AAA007 O20,Listonella anguillarum M3,marine gamma proteobacterium HTCC2080,marine gamma proteobacterium HTCC2143,marine gamma proteobacterium HTCC2148,SAR86 cluster bacterium SAR86C,SAR86 cluster bacterium SAR86D,SAR86 cluster bacterium SAR86E,Thiorhodovibrio 970,Candidatus Regiella insecticola LSR1,Candidatus Regiella insecticola R5 15 |
| Zetaproteobacteria | Mariprofundus ferrooxydans PV 1 |
| Spirochaetes | Borrelia afzelii ACA 1,Borrelia afzelii HLJ01,Borrelia afzelii PKo,Borrelia bissettii DN127,Borrelia burgdorferi 118a,Borrelia burgdorferi 156a,Borrelia burgdorferi 29805,Borrelia burgdorferi 64b,Borrelia burgdorferi 72a,Borrelia burgdorferi 80a,Borrelia burgdorferi 94a,Borrelia burgdorferi B31,Borrelia burgdorferi Bol26,Borrelia burgdorferi CA382,Borrelia burgdorferi CA 11 2a,Borrelia burgdorferi JD1,Borrelia burgdorferi N40,Borrelia burgdorferi WI91 23,Borrelia burgdorferi ZS7,Borrelia crocidurae Achema,Borrelia duttonii Ly,Borrelia garinii BgVir,Borrelia garinii Far04,Borrelia garinii NMJW1,Borrelia garinii PBi,Borrelia garinii PBr,Borrelia hermsii DAH,Borrelia miyamotoi LB 2001,Borrelia recurrentis A1,Borrelia spielmanii A14S,Borrelia SV1,Borrelia turicatae 91E135,Brachyspira 30446,Brachyspira hampsonii 30599,Brachyspira hyodysenteriae WA1,Brachyspira intermedia PWS A,Brachyspira murdochii DSM 12563,Brachyspira pilosicoli 95 1000,Brachyspira pilosicoli B2904,Brachyspira pilosicoli P43 6 78,Brachyspira pilosicoli WesB,Leptospira alexanderi serovar Manhao 3 L 60,Leptospira biflexa serovar Patoc Patoc 1 Ames,Leptospira biflexa serovar Patoc Patoc 1 Paris,Leptospira borgpetersenii 200701203,Leptospira borgpetersenii 200801926,Leptospira borgpetersenii 200901122,Leptospira borgpetersenii Brem 307,Leptospira borgpetersenii Brem 328,Leptospira borgpetersenii Noumea 25,Leptospira borgpetersenii serovar Castellonis 200801910,Leptospira borgpetersenii serovar Hardjo bovis JB197,Leptospira borgpetersenii serovar Hardjo bovis L550,Leptospira borgpetersenii serovar Javanica MK146,Leptospira borgpetersenii serovar Mini 200901116,Leptospira borgpetersenii serovar Pomona 200901868,Leptospira borgpetersenii UI 09149,Leptospira broomii 5399,Leptospira Fiocruz LV3954,Leptospira Fiocruz LV4135,Leptospira inadai serovar Lyme 10,Leptospira interrogans 2002000621,Leptospira interrogans 2002000623,Leptospira interrogans 2002000624,Leptospira interrogans 2002000626,Leptospira interrogans 2006001854,Leptospira interrogans Brem 329,Leptospira interrogans C10069,Leptospira interrogans FPW1039,Leptospira interrogans FPW2026,Leptospira interrogans HAI1536,Leptospira interrogans L0996,Leptospira interrogans L1207,Leptospira interrogans MMD3731,Leptospira interrogans serovar Australis 200703203,Leptospira interrogans serovar Autumnalis LP101,Leptospira interrogans serovar Bataviae 2006006959,Leptospira interrogans serovar Bataviae 2006006976,Leptospira interrogans serovar Bataviae HAI135,Leptospira interrogans serovar Bataviae Kariadi Satu,Leptospira interrogans serovar Bataviae L1111,Leptospira interrogans serovar Bataviae Swart,Leptospira interrogans serovar Bataviae UI 08561,Leptospira interrogans serovar Bataviae UT234,Leptospira interrogans serovar Bratislava Brem 137,Leptospira interrogans serovar Bulgarica Mallika,Leptospira interrogans serovar Canicola Fiocruz LV133,Leptospira interrogans serovar Canicola HAI0024,Leptospira interrogans serovar Canicola LT1962,Leptospira interrogans serovar Copenhageni 2001025091,Leptospira interrogans serovar Copenhageni 2006007831,Leptospira interrogans serovar Copenhageni Fiocruz LV192,Leptospira interrogans serovar Copenhageni Fiocruz LV199,Leptospira interrogans serovar Copenhageni Fiocruz LV204,Leptospira interrogans serovar Copenhageni Fiocruz LV212,Leptospira interrogans serovar Copenhageni Fiocruz LV239,Leptospira interrogans serovar Copenhageni Fiocruz LV2752,Leptospira interrogans serovar Copenhageni Fiocruz LV2756C6,Leptospira interrogans serovar Copenhageni Fiocruz LV2759,Leptospira interrogans serovar Copenhageni Fiocruz LV2763,Leptospira interrogans serovar Copenhageni Fiocruz LV2767,Leptospira interrogans serovar Copenhageni Fiocruz LV2772,Leptospira interrogans serovar Copenhageni Fiocruz LV2776,Leptospira interrogans serovar Copenhageni Fiocruz LV2787,Leptospira interrogans serovar Copenhageni Fiocruz LV2790,Leptospira interrogans serovar Copenhageni Fiocruz LV2791,Leptospira interrogans serovar Copenhageni Fiocruz LV2799,Leptospira interrogans serovar Copenhageni Fiocruz LV2804,Leptospira interrogans serovar Copenhageni Fiocruz LV2805,Leptospira interrogans serovar Copenhageni Fiocruz LV2806,Leptospira interrogans serovar Copenhageni Fiocruz LV2811,Leptospira interrogans serovar Copenhageni Fiocruz LV2812,Leptospira interrogans serovar Copenhageni Fiocruz LV2825,Leptospira interrogans serovar Copenhageni Fiocruz LV2832,Leptospira interrogans serovar Copenhageni Fiocruz LV2897,Leptospira interrogans serovar Copenhageni Fiocruz LV2948,Leptospira interrogans serovar Copenhageni Fiocruz LV2953,Leptospira interrogans serovar Copenhageni Fiocruz LV2958,Leptospira interrogans serovar Copenhageni Fiocruz LV2959,Leptospira interrogans serovar Copenhageni Fiocruz LV2973,Leptospira interrogans serovar Copenhageni Fiocruz LV3086,Leptospira interrogans serovar Copenhageni Fiocruz LV3094,Leptospira interrogans serovar Copenhageni Fiocruz LV3096,Leptospira interrogans serovar Copenhageni Fiocruz LV3323,Leptospira interrogans serovar Copenhageni Fiocruz LV3373,Leptospira interrogans serovar Copenhageni Fiocruz LV3737,Leptospira interrogans serovar Copenhageni Fiocruz LV3738,Leptospira interrogans serovar Copenhageni Fiocruz LV3834,Leptospira interrogans serovar Copenhageni Fiocruz LV3879,Leptospira interrogans serovar Copenhageni Fiocruz R154,Leptospira interrogans serovar Copenhageni Fiocruz R83,Leptospira interrogans serovar Copenhageni HAI0156,Leptospira interrogans serovar Copenhageni HAI0188,Leptospira interrogans serovar Copenhageni LT2050,Leptospira interrogans serovar Copenhageni P2518,Leptospira interrogans serovar Copenhageni R066,Leptospira interrogans serovar Copenhageni R103,Leptospira interrogans serovar Djasiman LT1649,Leptospira interrogans serovar Grippotyphosa 2006006986,Leptospira interrogans serovar Grippotyphosa Andaman,Leptospira interrogans serovar Grippotyphosa LT2186,Leptospira interrogans serovar Grippotyphosa UI 08368,Leptospira interrogans serovar Grippotyphosa UI 08434,Leptospira interrogans serovar Grippotyphosa UI 12764,Leptospira interrogans serovar Grippotyphosa UI 12769,Leptospira interrogans serovar Hebdomadis R499,Leptospira interrogans serovar Icterohaemorrhagiae P2422,Leptospira interrogans serovar Icterohaemorrhagiae P2547,Leptospira interrogans serovar Icterohaemorrhagiae P2554,Leptospira interrogans serovar Icterohaemorrhagiae Verdun HP,Leptospira interrogans serovar Icterohaemorrhagiae Verdun LP,Leptospira interrogans serovar Lai,Leptospira interrogans serovar Lai 56601,Leptospira interrogans serovar Lai IPAV,Leptospira interrogans serovar Lai Langkawi,Leptospira interrogans serovar Lai LPS mutant,Leptospira interrogans serovar Lora TE 1992,Leptospira interrogans serovar Manilae M001 Tn Mutant Parent,Leptospira interrogans serovar Manilae M1352 LPS mutant,Leptospira interrogans serovar Manilae M776 fur mutant,Leptospira interrogans serovar Manilae M874 LA0615 mutant,Leptospira interrogans serovar Manilae M933 lip32 mutant,Leptospira interrogans serovar Medanensis L0448,Leptospira interrogans serovar Medanensis L0887,Leptospira interrogans serovar Medanensis UT053,Leptospira interrogans serovar Muenchen Brem 129,Leptospira interrogans serovar Pomona,Leptospira interrogans serovar Pomona CSL10083,Leptospira interrogans serovar Pomona Fox 32256,Leptospira interrogans serovar Pomona Kennewicki LC82 25,Leptospira interrogans serovar Pomona UT364,Leptospira interrogans serovar Pyrogenes 2006006956,Leptospira interrogans serovar Pyrogenes 2006006960,Leptospira interrogans serovar Pyrogenes 200701872,Leptospira interrogans serovar Pyrogenes L0374,Leptospira interrogans serovar Pyrogenes R168,Leptospira interrogans serovar Valbuzzi,Leptospira interrogans serovar Zanoni LT2156,Leptospira interrogans UI 08452,Leptospira interrogans UI 09600,Leptospira interrogans UI 12621,Leptospira interrogans UI 12758,Leptospira interrogans UI 13372,Leptospira kirschneri 200801774,Leptospira kirschneri 200801925,Leptospira kirschneri 200802841,Leptospira kirschneri 200803703,Leptospira kirschneri 2008720114,Leptospira kirschneri H1,Leptospira kirschneri H2,Leptospira kirschneri serovar Bim 1051,Leptospira kirschneri serovar Grippotyphosa Moskva,Leptospira kirschneri serovar Grippotyphosa RM52,Leptospira kirschneri serovar Sokoine RM1,Leptospira kirschneri serovar Valbuzzi 200702274,Leptospira kmetyi serovar Malaysia Bejo Iso9,Leptospira licerasiae MMD4847,Leptospira licerasiae serovar Varillal MMD0835,Leptospira licerasiae serovar Varillal VAR 010,Leptospira meyeri serovar Hardjo Went 5,Leptospira noguchii 2001034031,Leptospira noguchii 2006001870,Leptospira noguchii 2007001578,Leptospira noguchii Bonito,Leptospira noguchii Cascata,Leptospira noguchii Hook,Leptospira noguchii serovar Autumnalis ZUN142,Leptospira santarosai 2000027870,Leptospira santarosai 2000030832,Leptospira santarosai 200403458,Leptospira santarosai 200702252,Leptospira santarosai AIM,Leptospira santarosai CBC1416,Leptospira santarosai CBC1531,Leptospira santarosai CBC379,Leptospira santarosai CBC523,Leptospira santarosai HAI134,Leptospira santarosai HAI1380,Leptospira santarosai HAI1594,Leptospira santarosai HAI821,Leptospira santarosai JET,Leptospira santarosai MOR084,Leptospira santarosai serovar Arenal MAVJ 401,Leptospira santarosai serovar Shermani LT 821,Leptospira santarosai ST188,Leptospira santarosai ZUN179,Leptospira weilii 2006001853,Leptospira weilii 2006001855,Leptospira weilii LNT 1234,Leptospira weilii serovar Topaz LT2116,Leptospira weilii UI 13098,Sphaerochaeta pleomorpha Grapes,Spirochaeta africana DSM 8902,Spirochaeta Buddy,Spirochaeta caldaria DSM 7334,Spirochaeta coccoides DSM 17374,Spirochaeta smaragdinae DSM 11293,Spirochaeta thermophila DSM 6192,Spirochaeta thermophila DSM 6578,Treponema azotonutricium ZAS 9,Treponema brennaborense DSM 12168,Treponema denticola AL 2,Treponema denticola ASLM,Treponema denticola ATCC 33520,Treponema denticola ATCC 33521,Treponema denticola ATCC 35404,Treponema denticola ATCC 35405,Treponema denticola F0402,Treponema denticola H1 T,Treponema denticola H 22,Treponema denticola MYR T,Treponema denticola OTK,Treponema denticola SP33,Treponema denticola SP37,Treponema denticola US Trep,Treponema JC4,Treponema pallidum Chicago,Treponema pallidum DAL 1,Treponema pallidum Fribourg Blanc,Treponema pallidum Mexico A,Treponema pallidum Nichols,Treponema pallidum pertenue CDC2,Treponema pallidum pertenue Gauthier,Treponema pallidum pertenue SamoaD,Treponema pallidum SS14,Treponema paraluiscuniculi Cuniculi A,Treponema pedis T A4,Treponema primitia ZAS 1,Treponema primitia ZAS 2,Treponema saccharophilum DSM 2985,Treponema succinifaciens DSM 2489,Treponema vincentii ATCC 35580,Turneriella parva DSM 21527,Leptonema illini DSM 21528 |
| Synergistetes | Aminobacterium colombiense DSM 12261,Anaerobaculum mobile DSM 13181,Synergistetes bacterium SGP1,Thermanaerovibrio acidaminovorans DSM 6589,Thermanaerovibrio velox DSM 12556,Thermovirga lienii DSM 17291,Dethiosulfovibrio peptidovorans DSM 11002,Jonquetella anthropi DSM 22815,Jonquetella anthropi E3 33 E1,Pyramidobacter piscolens W5455,Synergistes 3 1 syn1 |
| Tenericutes | Acholeplasma laidlawii PG 8A,Candidatus Mycoplasma haemolamae Purdue,Candidatus Mycoplasma haemominutum Birmingham 1,Candidatus Phytoplasma australiense,Candidatus Phytoplasma mali,Candidatus Phytoplasma solani 284 09,Mesoplasma florum L1,Mesoplasma florum W37,Mycoplasma agalactiae,Mycoplasma agalactiae 14628,Mycoplasma agalactiae PG2,Mycoplasma alligatoris A21JP2,Mycoplasma anatis 1340,Mycoplasma arthritidis 158L3 1,Mycoplasma bovis HB0801,Mycoplasma bovis Hubei 1,Mycoplasma bovis PG45,Mycoplasma canis PG 14,Mycoplasma canis UF31,Mycoplasma canis UF33,Mycoplasma canis UFG1,Mycoplasma canis UFG4,Mycoplasma capricolum ATCC 27343,Mycoplasma columbinum SF7,Mycoplasma conjunctivae,Mycoplasma crocodyli MP145,Mycoplasma cynos C142,Mycoplasma fermentans JER,Mycoplasma fermentans M64,Mycoplasma fermentans PG18,Mycoplasma G5847,Mycoplasma gallisepticum CA06 2006 052 5 2P,Mycoplasma gallisepticum F,Mycoplasma gallisepticum NC06 2006 080 5 2P,Mycoplasma gallisepticum NC08 2008 031 4 3P,Mycoplasma gallisepticum NC95 13295 2 2P,Mycoplasma gallisepticum NC96 1596 4 2P,Mycoplasma gallisepticum NY01 2001 047 5 1P,Mycoplasma gallisepticum R high,Mycoplasma gallisepticum R low,Mycoplasma gallisepticum S6,Mycoplasma gallisepticum VA94 7994 1 7P,Mycoplasma gallisepticum WI01 2001 043 13 2P,Mycoplasma genitalium G37,Mycoplasma genitalium M2288,Mycoplasma genitalium M2321,Mycoplasma genitalium M6282,Mycoplasma genitalium M6320,Mycoplasma haemocanis Illinois,Mycoplasma haemofelis Langford 1,Mycoplasma haemofelis Ohio2,Mycoplasma hominis ATCC 23114,Mycoplasma hyopneumoniae 168,Mycoplasma hyopneumoniae 168 L,Mycoplasma hyopneumoniae 232,Mycoplasma hyopneumoniae 7422,Mycoplasma hyopneumoniae 7448,Mycoplasma hyopneumoniae J,Mycoplasma hyorhinis DBS 1050,Mycoplasma hyorhinis GDL 1,Mycoplasma hyorhinis HUB 1,Mycoplasma hyorhinis MCLD,Mycoplasma hyorhinis SK76,Mycoplasma iowae 695,Mycoplasma leachii 99 014 6,Mycoplasma leachii PG50,Mycoplasma mobile 163K,Mycoplasma mycoides capri LC 95010,Mycoplasma mycoides SC Gladysdale,Mycoplasma mycoides SC PG1,Mycoplasma ovipneumoniae SC01,Mycoplasma ovis Michigan,Mycoplasma parvum Indiana,Mycoplasma penetrans HF 2,Mycoplasma pneumoniae 309,Mycoplasma pneumoniae FH,Mycoplasma pneumoniae M129,Mycoplasma pneumoniae M129 B7,Mycoplasma pulmonis UAB CTIP,Mycoplasma putrefaciens KS1,Mycoplasma putrefaciens Mput9231,Mycoplasma suis Illinois,Mycoplasma suis KI3806,Mycoplasma synoviae 53,Mycoplasma wenyonii Massachusetts,Ureaplasma parvum serovar 14 ATCC 33697,Ureaplasma parvum serovar 1 ATCC 27813,Ureaplasma parvum serovar 3 ATCC 27815,Ureaplasma parvum serovar 3 ATCC 700970,Ureaplasma parvum serovar 6 ATCC 27818,Ureaplasma urealyticum serovar 10 ATCC 33699,Ureaplasma urealyticum serovar 11 ATCC 33695,Ureaplasma urealyticum serovar 12 ATCC 33696,Ureaplasma urealyticum serovar 13 ATCC 33698,Ureaplasma urealyticum serovar 2 ATCC 27814,Ureaplasma urealyticum serovar 4 ATCC 27816,Ureaplasma urealyticum serovar 5 ATCC 27817,Ureaplasma urealyticum serovar 7 ATCC 27819,Ureaplasma urealyticum serovar 8 ATCC 27618,Ureaplasma urealyticum serovar 9 ATCC 33175,Aster yellows witches broom phytoplasma AYWB,Spiroplasma apis B31,Spiroplasma chrysopicola DF 1,Spiroplasma diminutum CUAS 1,Spiroplasma melliferum IPMB4A,Spiroplasma melliferum KC3,Spiroplasma syrphidicola EA 1,Spiroplasma taiwanense CT 1,Onion yellows phytoplasma OY M,Peanut witches broom phytoplasma NTU2011,Strawberry lethal yellows phytoplasma CPA NZSb11 |
| Thermodesulfobacteria | Thermodesulfatator indicus DSM 15286,Thermodesulfobacterium OPB45 |
| Thermotogae | Fervidobacterium nodosum Rt17 B1,Fervidobacterium pennivorans DSM 9078,Kosmotoga olearia TBF 19 5 1,Marinitoga piezophila KA3,Mesotoga prima MesG1 Ag 4 2,Petrotoga mobilis SJ95,Thermotoga neapolitana DSM 4359,Thermotoga petrophila RKU 1,Thermotoga RQ2,Thermotoga thermarum DSM 5069,Thermosipho africanus H17ap60334,Thermosipho africanus TCF52B,Thermosipho melanesiensis BI429,Thermotoga EMP,Thermotoga lettingae TMO,Thermotoga maritima MSB8,Thermotoga naphthophila RKU 10, |
| Crenarchaeota | Acidianus hospitalis W1,Acidilobus saccharovorans 345 15,Aeropyrum camini SY1 JCM 12091,Aeropyrum pernix K1,Caldisphaera lagunensis DSM 15908,Caldivirga maquilingensis IC 167,Desulfurococcus fermentans DSM 16532,Desulfurococcus kamchatkensis 1221n,Desulfurococcus mucosus DSM 2162,Fervidicoccus fontis Kam940,Hyperthermus butylicus DSM 5456,Ignicoccus hospitalis KIN4 I,Ignisphaera aggregans DSM 17230,Metallosphaera cuprina Ar 4,Metallosphaera sedula DSM 5348,Metallosphaera yellowstonensis MK1,Pyrobaculum 1860,Pyrobaculum aerophilum IM2,Pyrobaculum arsenaticum DSM 13514,Pyrobaculum calidifontis JCM 11548,Pyrobaculum islandicum DSM 4184,Pyrobaculum neutrophilum V24Sta,Pyrobaculum oguniense TE7,Pyrolobus fumarii 1A,Staphylothermus hellenicus DSM 12710,Staphylothermus marinus F1,Sulfolobus acidocaldarius DSM 639,Sulfolobus acidocaldarius N8,Sulfolobus acidocaldarius Ron12 I,Sulfolobus acidocaldarius SUSAZ,Sulfolobus islandicus HVE10 4,Sulfolobus islandicus L D 8 5,Sulfolobus islandicus L S 2 15,Sulfolobus islandicus LAL14 1,Sulfolobus islandicus M 14 25,Sulfolobus islandicus M 16 27,Sulfolobus islandicus M 16 4,Sulfolobus islandicus REY15A,Sulfolobus islandicus Y G 57 14,Sulfolobus islandicus Y N 15 51,Sulfolobus solfataricus 98 2,Sulfolobus solfataricus P2,Sulfolobus tokodaii 7,Thermofilum 1910b,Thermofilum pendens Hrk 5,Thermogladius 1633,Thermoproteus tenax Kra 1,Thermoproteus uzoniensis 768 20,Thermosphaera aggregans DSM 11486,Vulcanisaeta distributa DSM 14429,Vulcanisaeta moutnovskia 768 28 |
| Euryarchaeota | Aciduliprofundum boonei T469,Aciduliprofundum MAR08 339,Archaeoglobus fulgidus DSM 4304,Archaeoglobus profundus DSM 5631,Archaeoglobus sulfaticallidus PM70 1,Archaeoglobus veneficus SNP6,Ferroglobus placidus DSM 10642,Halalkalicoccus jeotgali B3,Haloarcula amylolytica JCM 13557,Haloarcula argentinensis DSM 12282,Haloarcula californiae ATCC 33799,Haloarcula hispanica ATCC 33960,Haloarcula hispanica N601,Haloarcula japonica DSM 6131,Haloarcula marismortui ATCC 43049,Haloarcula sinaiiensis ATCC 33800,Haloarcula vallismortis ATCC 29715,Halobacterium DL1,Halobacterium NRC 1,Halobacterium salinarum R1,Haloferax alexandrinus JCM 10717,Haloferax ATCC BAA 644,Haloferax ATCC BAA 645,Haloferax ATCC BAA 646,Haloferax BAB2207,Haloferax denitrificans ATCC 35960,Haloferax elongans ATCC BAA 1513,Haloferax gibbonsii ATCC 33959,Haloferax larsenii JCM 13917,Haloferax lucentense DSM 14919,Haloferax mediterranei ATCC 33500,Haloferax mucosum ATCC BAA 1512,Haloferax prahovense DSM 18310,Haloferax sulfurifontis ATCC BAA 897,Haloferax volcanii DS2,Halogeometricum borinquense DSM 11551,Halomicrobium mukohataei DSM 12286,Halopiger xanaduensis SH 6,Haloquadratum walsbyi C23,Haloquadratum walsbyi DSM 16790,Halorhabdus tiamatea SARL4B,Halorhabdus utahensis DSM 12940,Halorubrum aidingense JCM 13560,Halorubrum arcis JCM 13916,Halorubrum californiensis DSM 19288,Halorubrum coriense DSM 10284,Halorubrum distributum JCM 10118,Halorubrum distributum JCM 9100,Halorubrum hochstenium ATCC 700873,Halorubrum kocurii JCM 14978,Halorubrum lacusprofundi ATCC 49239,Halorubrum lipolyticum DSM 21995,Halorubrum litoreum JCM 13561,Halorubrum saccharovorum DSM 1137,Halorubrum tebenquichense DSM 14210,Halorubrum terrestre JCM 10247,Haloterrigena limicola JCM 13563,Haloterrigena salina JCM 13891,Haloterrigena thermotolerans DSM 11522,Haloterrigena turkmenica DSM 5511,Halovivax asiaticus JCM 14624,Halovivax ruber XH 70,Methanobacterium AL 21,Methanobacterium formicicum DSM 3637,Methanobacterium Maddingley MBC34,Methanobacterium MB1,Methanobacterium SWAN 1,Methanobrevibacter AbM4,Methanobrevibacter ruminantium M1,Methanobrevibacter smithii ATCC 35061,Methanobrevibacter smithii DSM 2374,Methanobrevibacter smithii DSM 2375,Methanocaldococcus fervens AG86,Methanocaldococcus FS406 22,Methanocaldococcus infernus ME,Methanocaldococcus jannaschii DSM 2661,Methanocaldococcus vulcanius M7,Methanocella arvoryzae MRE50,Methanocella conradii HZ254,Methanocella paludicola SANAE,Methanococcoides burtonii DSM 6242,Methanococcus aeolicus Nankai 3,Methanococcus maripaludis C5,Methanococcus maripaludis C6,Methanococcus maripaludis C7,Methanococcus maripaludis S2,Methanococcus maripaludis X1,Methanococcus vannielii SB,Methanococcus voltae A3,Methanocorpusculum labreanum Z,Methanoculleus bourgensis MS2,Methanoculleus marisnigri JR1,Methanohalobium evestigatum Z 7303,Methanohalophilus mahii DSM 5219,Methanolobus psychrophilus R15,Methanomethylovorans hollandica DSM 15978,Methanoplanus limicola DSM 2279,Methanoplanus petrolearius DSM 11571,Methanopyrus kandleri AV19,Methanoregula boonei 6A8,Methanoregula formicicum SMSP,Methanosaeta concilii GP6,Methanosaeta harundinacea 6Ac,Methanosaeta thermophila PT,Methanosalsum zhilinae DSM 4017,Methanosarcina acetivorans C2A,Methanosarcina barkeri Fusaro,Methanosarcina mazei Go1,Methanosarcina mazei Tuc01,Methanosphaera stadtmanae DSM 3091,Methanosphaerula palustris E1 9c,Methanospirillum hungatei JF 1,Methanothermobacter marburgensis Marburg,Methanothermobacter thermautotrophicus Delta H,Methanothermococcus okinawensis IH1,Methanothermus fervidus DSM 2088,Methanotorris formicicus Mc S 70,Methanotorris igneus Kol 5,Natrialba aegyptia DSM 13077,Natrialba asiatica DSM 12278,Natrialba chahannaoensis JCM 10990,Natrialba hulunbeirensis JCM 10989,Natrialba magadii ATCC 43099,Natrialba taiwanensis DSM 12281,Natrinema altunense JCM 12890,Natrinema gari JCM 14663,Natrinema J7,Natrinema pallidum DSM 3751,Natrinema pellirubrum DSM 15624,Natrinema versiforme JCM 10478,Natronobacterium gregoryi SP2,Natronococcus amylolyticus DSM 10524,Natronococcus jeotgali DSM 18795,Natronococcus occultus SP4,Natronomonas moolapensis 8 8 11,Natronomonas pharaonis DSM 2160,Picrophilus torridus DSM 9790,Pyrococcus abyssi GE5,Pyrococcus furiosus COM1,Pyrococcus furiosus DSM 3638,Pyrococcus horikoshii OT3,Pyrococcus NA2,Pyrococcus ST04,Pyrococcus yayanosii CH1,Thermococcus 4557,Thermococcus AM4,Thermococcus barophilus MP,Thermococcus CL1,Thermococcus gammatolerans EJ3,Thermococcus kodakarensis KOD1,Thermococcus litoralis DSM 5473,Thermococcus onnurineus NA1,Thermococcus sibiricus MM 739,Thermococcus zilligii AN1,Thermoplasma acidophilum DSM 1728,Thermoplasma volcanium GSS1,Thermoplasmatales archaeon BRNA1,Thermoplasmatales archaeon N05,Thermoplasmatales archaeon SCGC AB 540 F20,Ferroplasma acidarmanus fer1,Haladaptatus paucihalophilus DX253,Halobiforma lacisalsi AJ5,Halobiforma nitratireducens JCM 10879,Halococcus hamelinensis 100A6,Halococcus morrhuae DSM 1307,Halococcus saccharolyticus DSM 5350,Halococcus salifodinae DSM 8989,Halococcus thailandensis JCM 13552,Halogranum salarium B 1,Halosarcina pallida JCM 14848,Halosimplex carlsbadense 2 9 1,Methanofollis liminatans DSM 4140,Methanolinea tarda NOBI 1,Methanomassiliicoccus Mx1 Issoire,Natronolimnobius innermongolicus JCM 12255,Natronorubrum bangense JCM 10635,Natronorubrum sulfidifaciens JCM 14089,Natronorubrum tibetense GA33,Salinarchaeum laminariae Harcht Bsk1 |
| Korarchaeota | Candidatus Korarchaeum cryptofilum OPF8 |
| Thaumarchaeota | Candidatus Nitrosopumilus AR2,Candidatus Nitrosopumilus koreensis AR1,Candidatus Nitrososphaera gargensis Ga9 2,Cenarchaeum symbiosum A,Nitrosopumilus maritimus SCM1,Nitrosopumilus MY1,Thaumarchaeota archaeon E09,Candidatus Caldiarchaeum subterraneum,Candidatus Nitrosoarchaeum limnia BG20,Candidatus Nitrosoarchaeum limnia SFB1,marine archaeal group 1 BD31 |
| Nanoarchaeota | Nanoarchaeum equitans Kin4 M |
| Nanohaloarchaeota | Candidatus Haloredivivus G17 |
| Alveolates | Cryptosporidium parvum,Plasmodium falciparum,Plasmodium falciparum OLD,Theileria annulata,Theileria parva |
| Amoeboflagellate | Dictyostelium discoideum |
| Euglenozoa | Leishmania braziliensis,Leishmania donovani,Leishmania infantum,Leishmania major,Trypanosoma brucei |
| Microsporidians | Encephalitozoon cuniculi,Encephalitozoon intestinalis ATCC 50506 |
| Ascomycetes | Aspergillus fumigatus,Aspergillus nidulans FGSC A4,Aspergillus niger CBS 513 88,Aspergillus oryzae RIB40,Candida albicans,Candida dubliniensis CD36,Candida glabrata,Candida glabrata CBS138,Debaryomyces hansenii CBS767,Eremothecium cymbalariae DBVPG 7215,Eremothecium gossypii,Kazachstania africana CBS 2517,Kluyveromyces lactis NRRL Y-1140,Lachancea thermotolerans CBS 6340,Magnaporthe grisea 70-15,Myceliophthora thermophila ATCC 42464,Naumovozyma castellii CBS 4309,Naumovozyma dairenensis CBS 421,Neurospora crassa,Pichia pastoris GS115,Pichia stipitis CBS 6054,Podospora anserina DSM 980,Saccharomyces cerevisiae,Schizosaccharomyces pombe,Schizosaccharomyces pombe OLD,Tetrapisispora phaffii CBS 4417,Thielavia terrestris NRRL 8126,Torulaspora delbrueckii CBS 1146,Yarrowia lipolytica CLIB122,Zygosaccharomyces rouxii CBS 732,Gibberella zeae PH-1 |
| Basidiomycetes | Cryptococcus gattii WM276,Cryptococcus neoformans var JEC21 |
| Eudicots | Arabidopsis thaliana,Populus trichocarpa |
| Monocots | Oryza sativa OLD |
| Nematodes | Caenorhabditis elegans |
| Arthropods | Anopheles gambiae,Culex nigripalpus NPV,Drosophila melanogaster,Drosophila pseudoobscura,Megachile rotundata,Metaseiulus occidentalis,Tribolleum castaneum |
| Chordates | Branchiostoma floridae,Taeniopygia guttata,Takifugu rubripes,Ceratotherium simum simum,Xiphophorus maculatus,Geospiza fortis,Maylandia zebra,Pseudopodoces humilis,Pundamilia nyererei,Zonotrichia albicollis |

*The species Thermobaculum terrenum ATCC BAA 798 is annotated as an unclassified Bacterium in the NCBI taxonomy database (at 5/18/2015). However, we considered it to be a member of the Chloroflexi phylum, following Kunisawa (**10.1099/ijs.0.026088-0**).
