# Supplementary material for: A survey of HK, HPt, and RR domains and their organization in two-component systems and phosphorelay proteins of organisms with fully sequenced genomes
Source: PeerJ. 2015 Aug 13;3:e1183. doi: 10.7717/peerj.1183 (PMC4558063; doi:10.7717/peerj.1183)
Supplement: Table S2 — Phylum abbreviations are given in Table 1. [file peerj-03-1183-s003.docx]

**Supplementary Table 2. Percentage of each TCS/PR protein type per phylum.** Phylum abbreviations are given in Table 1. Only phyla with proteins containing HK, RR or HPt domains are represented. Korarchaeota, Nanoarchaeota, Nanohaloarchaeota and phyla from the animal kingdom do not appear in the table because we have not found any protein containing HK, RR or HPt domains in the surveyed species classified in these phyla.

| Phylum | RR | HK | HKRR | HPt | HKRRHPt | HK_1_RRHK_2_ | HKRR_1_HPtRR_2_ | HK_1_RR_1_HK_2_RR_2_ |
| --- | --- | --- | --- | --- | --- | --- | --- | --- |
| At | 55.04 | 43.23 | 1.00 | 0.30 | 0.04 | 0.01 | 0.05 | 0.00 |
| Aq | 55.80 | 38.41 | 0.00 | 1.81 | 0.00 | 0.00 | 0.00 | 0.00 |
| Ar | 61.54 | 30.77 | 3.85 | 0.00 | 0.00 | 0.00 | 0.00 | 0.00 |
| Ba | 51.36 | 36.04 | 9.16 | 1.04 | 0.91 | 0.08 | 0.15 | 0.00 |
| Cb | 38.37 | 33.06 | 18.37 | 0.82 | 0.82 | 0.00 | 2.24 | 0.00 |
| Cd | 42.86 | 50.00 | 7.14 | 0.00 | 0.00 | 0.00 | 0.00 | 0.00 |
| Cm | 33.19 | 66.18 | 0.00 | 0.21 | 0.42 | 0.00 | 0.00 | 0.00 |
| L | 42.50 | 42.50 | 7.50 | 5.00 | 2.50 | 0.00 | 0.00 | 0.00 |
| V | 46.21 | 29.66 | 15.07 | 0.99 | 1.18 | 0.20 | 0.39 | 0.20 |
| Cf | 50.14 | 39.02 | 7.00 | 0.32 | 0.72 | 0.00 | 0.05 | 0.00 |
| Cr | 57.29 | 32.29 | 4.17 | 0.00 | 1.04 | 0.00 | 1.04 | 0.00 |
| Cy | 44.97 | 31.94 | 13.53 | 1.03 | 1.80 | 0.26 | 0.88 | 0.20 |
| Df | 51.25 | 34.69 | 6.88 | 0.31 | 2.81 | 0.00 | 0.63 | 0.00 |
| Dt | 54.14 | 41.77 | 1.28 | 0.23 | 0.00 | 0.47 | 0.00 | 0.00 |
| Dc | 50.00 | 46.43 | 3.57 | 0.00 | 0.00 | 0.00 | 0.00 | 0.00 |
| El | 50.00 | 41.67 | 8.33 | 0.00 | 0.00 | 0.00 | 0.00 | 0.00 |
| Ac | 53.43 | 33.04 | 8.79 | 0.87 | 0.10 | 0.48 | 0.39 | 0.29 |
| Fb | 48.00 | 16.00 | 16.00 | 4.00 | 0.00 | 0.00 | 4.00 | 0.00 |
| Fi | 54.90 | 42.69 | 0.44 | 0.60 | 0.08 | 0.12 | 0.04 | 0.00 |
| Fu | 59.74 | 37.65 | 1.09 | 0.87 | 0.22 | 0.00 | 0.00 | 0.00 |
| Ge | 47.58 | 32.26 | 14.52 | 0.81 | 2.42 | 0.00 | 0.00 | 0.00 |
| Ni | 53.42 | 24.66 | 15.07 | 2.74 | 0.00 | 0.00 | 1.37 | 0.00 |
| Nt | 56.99 | 29.72 | 3.50 | 1.40 | 1.40 | 0.00 | 1.40 | 0.00 |
| Pl | 53.67 | 25.84 | 10.84 | 1.47 | 1.89 | 0.00 | 1.14 | 0.00 |
| A | 53.36 | 31.75 | 7.66 | 1.28 | 1.15 | 0.03 | 0.24 | 0.07 |
| B | 54.56 | 34.34 | 5.83 | 0.54 | 1.43 | 0.04 | 0.51 | 0.05 |
| D | 49.17 | 30.06 | 11.86 | 1.39 | 1.63 | 0.23 | 0.68 | 0.10 |
| E | 59.14 | 33.36 | 0.83 | 0.65 | 4.07 | 0.00 | 0.07 | 0.00 |
| G | 52.58 | 34.48 | 3.99 | 0.91 | 4.36 | 0.02 | 0.27 | 0.00 |
| Z | 46.07 | 22.47 | 26.97 | 0.00 | 2.25 | 0.00 | 1.12 | 0.00 |
| S | 49.08 | 32.84 | 9.93 | 1.40 | 0.33 | 0.91 | 0.09 | 0.00 |
| Sy | 57.14 | 34.43 | 1.10 | 1.83 | 2.20 | 0.00 | 0.73 | 0.00 |
| T | 54.31 | 40.10 | 0.00 | 5.58 | 0.00 | 0.00 | 0.00 | 0.00 |
| Th | 50.00 | 31.25 | 11.25 | 1.25 | 2.50 | 0.00 | 0.00 | 0.00 |
| Tt | 53.73 | 39.55 | 0.25 | 0.00 | 1.49 | 0.00 | 0.75 | 0.00 |
| C | 63.64 | 30.30 | 0.00 | 6.06 | 0.00 | 0.00 | 0.00 | 0.00 |
| Eu | 38.20 | 47.45 | 11.49 | 0.08 | 0.15 | 0.02 | 0.05 | 0.00 |
| Ta | 59.12 | 36.82 | 0.34 | 2.36 | 0.00 | 0.00 | 0.00 | 0.00 |
| Av | 0.00 | 50.00 | 0.00 | 50.00 | 0.00 | 0.00 | 0.00 | 0.00 |
| Am | 22.73 | 0.00 | 40.91 | 9.09 | 0.00 | 0.00 | 0.00 | 4.55 |
| Eg | 6.06 | 93.94 | 0.00 | 0.00 | 0.00 | 0.00 | 0.00 | 0.00 |
| Mi | 66.67 | 33.33 | 0.00 | 0.00 | 0.00 | 0.00 | 0.00 | 0.00 |
| As | 28.10 | 18.60 | 15.29 | 19.83 | 0.00 | 6.61 | 0.00 | 2.48 |
| Bs | 18.18 | 4.55 | 45.45 | 9.09 | 0.00 | 9.09 | 0.00 | 13.64 |
| Ed | 60.12 | 7.36 | 13.50 | 16.56 | 0.00 | 0.61 | 0.00 | 0.00 |
| M | 0.00 | 63.64 | 0.00 | 36.36 | 0.00 | 0.00 | 0.00 | 0.00 |
