# Supplementary material for: A survey of HK, HPt, and RR domains and their organization in two-component systems and phosphorelay proteins of organisms with fully sequenced genomes
Source: PeerJ. 2015 Aug 13;3:e1183. doi: 10.7717/peerj.1183 (PMC4558063; doi:10.7717/peerj.1183)
Supplement: Table S3 — Phylum abbreviations are given in Table 1. [file peerj-03-1183-s004.docx]

**Supplementary Table 3. Percentage of HK genes and RR genes that are neighbors in the genome to other TCS/PR genes.** Phylum abbreviations are given in Table 1. Only phyla with HK and RR genes are represented. Alveolates are omitted because we found only 1 HK protein and 1 HPt protein in 5 species surveyed.

| Phylum | Orphan HK | HK next to RR | HK next to RR and HK_2_ | HK next to RR, HK_2_ and RR_2_ | Orphan RR | RR next to HK | RR next to HPt | RR next to HKRRHPt | RR next to HKRRHK |
| --- | --- | --- | --- | --- | --- | --- | --- | --- | --- |
| At | 20,64 | 75,82 | 0,33 | 0,40 | 34,72 | 59,55 | 0,08 | 0,01 | 0,00 |
| Aq | 37,74 | 58,49 | 0,94 | 0,00 | 46,10 | 40,26 | 0,00 | 0,00 | 0,00 |
| Ar | 50,00 | 50,00 | 0,00 | 0,00 | 62,50 | 25,00 | 0,00 | 0,00 | 0,00 |
| Ba | 31,33 | 54,98 | 1,53 | 0,61 | 47,78 | 38,57 | 0,10 | 0,94 | 0,05 |
| Cb | 37,04 | 40,74 | 3,09 | 0,00 | 31,38 | 35,11 | 0,53 | 0,00 | 0,00 |
| Cd | 28,57 | 71,43 | 0,00 | 0,00 | 0,00 | 83,33 | 0,00 | 0,00 | 0,00 |
| Cm | 65,62 | 34,38 | 0,00 | 0,00 | 27,04 | 68,55 | 0,00 | 0,63 | 0,00 |
| L | 41,18 | 52,94 | 0,00 | 0,00 | 35,29 | 52,94 | 0,00 | 0,00 | 0,00 |
| V | 24,92 | 59,14 | 0,66 | 0,00 | 36,46 | 37,95 | 0,43 | 0,64 | 0,00 |
| Cf | 32,41 | 51,50 | 1,04 | 1,16 | 36,04 | 40,09 | 0,09 | 0,27 | 0,00 |
| Cr | 48,39 | 16,13 | 0,00 | 3,23 | 70,91 | 9,09 | 0,00 | 0,00 | 0,00 |
| Cy | 56,50 | 25,99 | 0,47 | 0,06 | 50,66 | 18,46 | 0,10 | 0,46 | 0,06 |
| Df | 36,04 | 53,15 | 1,80 | 0,90 | 39,63 | 35,98 | 0,00 | 3,66 | 0,00 |
| Dt | 31,01 | 60,89 | 1,12 | 0,84 | 37,93 | 46,98 | 0,00 | 0,00 | 0,22 |
| Dc | 30,77 | 61,54 | 0,00 | 0,00 | 42,86 | 57,14 | 0,00 | 0,00 | 0,00 |
| El | 60,00 | 20,00 | 0,00 | 0,00 | 66,67 | 16,67 | 0,00 | 0,00 | 0,00 |
| Ac | 28,65 | 58,48 | 0,00 | 1,46 | 41,41 | 36,17 | 0,18 | 0,18 | 0,18 |
| Fb | 0,00 | 75,00 | 0,00 | 0,00 | 33,33 | 25,00 | 0,00 | 0,00 | 0,00 |
| Fi | 12,15 | 84,82 | 0,34 | 0,37 | 29,32 | 65,96 | 0,08 | 0,06 | 0,14 |
| Fu | 10,40 | 86,99 | 0,29 | 0,00 | 40,80 | 54,83 | 0,55 | 0,18 | 0,00 |
| Ge | 20,00 | 65,00 | 0,00 | 2,50 | 28,81 | 44,07 | 0,00 | 3,39 | 0,00 |
| Ni | 33,33 | 61,11 | 0,00 | 0,00 | 46,15 | 28,21 | 0,00 | 0,00 | 0,00 |
| Nt | 11,76 | 61,18 | 2,35 | 1,18 | 30,06 | 31,90 | 0,00 | 1,23 | 0,00 |
| Pl | 37,91 | 48,90 | 0,37 | 0,18 | 53,53 | 23,54 | 0,88 | 1,41 | 0,00 |
| A | 32,43 | 51,81 | 0,84 | 2,84 | 42,09 | 30,83 | 0,72 | 0,76 | 0,01 |
| B | 13,04 | 77,21 | 0,46 | 0,94 | 27,85 | 48,59 | 0,20 | 1,31 | 0,02 |
| D | 35,58 | 39,04 | 1,12 | 0,83 | 40,22 | 23,87 | 0,46 | 1,02 | 0,00 |
| E | 24,20 | 72,50 | 0,37 | 0,31 | 48,85 | 40,90 | 0,02 | 6,68 | 0,00 |
| G | 11,86 | 81,24 | 0,41 | 0,25 | 26,41 | 53,27 | 0,46 | 3,28 | 0,02 |
| Z | 10,00 | 90,00 | 0,00 | 0,00 | 21,95 | 43,90 | 0,00 | 0,00 | 0,00 |
| S | 35,93 | 45,26 | 0,09 | 0,02 | 50,51 | 30,28 | 0,04 | 0,13 | 0,00 |
| Sy | 21,28 | 75,53 | 0,00 | 1,06 | 39,74 | 45,51 | 0,64 | 1,92 | 0,00 |
| T | 1,27 | 98,73 | 0,00 | 0,00 | 27,10 | 72,90 | 0,00 | 0,00 | 0,00 |
| Th | 36,00 | 60,00 | 0,00 | 0,00 | 37,50 | 37,50 | 0,00 | 0,00 | 0,00 |
| Tt | 38,36 | 58,49 | 0,00 | 0,63 | 42,59 | 43,06 | 0,00 | 2,31 | 0,00 |
| C | 50,00 | 50,00 | 0,00 | 0,00 | 76,19 | 23,81 | 0,00 | 0,00 | 0,00 |
| Eu | 69,37 | 18,61 | 0,43 | 0,11 | 51,56 | 23,12 | 0,00 | 0,04 | 0,00 |
| Ta | 35,78 | 40,37 | 0,92 | 0,00 | 50,86 | 25,14 | 0,00 | 0,00 | 0,00 |
| Eg | 45,16 | 0,00 | 0,00 | 0,00 | 100,00 | 0,00 | 0,00 | 0,00 | 0,00 |
| Mi | 100,00 | 0,00 | 0,00 | 0,00 | 100,00 | 0,00 | 0,00 | 0,00 | 0,00 |
| As | 100,00 | 0,00 | 0,00 | 0,00 | 100,00 | 0,00 | 0,00 | 0,00 | 0,00 |
| Bs | 100,00 | 0,00 | 0,00 | 0,00 | 100,00 | 0,00 | 0,00 | 0,00 | 0,00 |
| Ed | 100,00 | 0,00 | 0,00 | 0,00 | 59,18 | 0,00 | 0,00 | 0,00 | 0,00 |
|  |  |  |  |  |  |  |  |  |  |
