# Supplementary material for: A survey of HK, HPt, and RR domains and their organization in two-component systems and phosphorelay proteins of organisms with fully sequenced genomes
Source: PeerJ. 2015 Aug 13;3:e1183. doi: 10.7717/peerj.1183 (PMC4558063; doi:10.7717/peerj.1183)
Supplement: Table S5 — Only species with HK and RR genes are taken into account in the percentages. Phylum abbreviations are given in Table 1. [file peerj-03-1183-s007.docx]

**Supplementary Table 5. Odds ratios (ratio between the observed and the randomly expected frequency) of HK genes located in the genome next to RR, HK_2_ and RR_2_ genes.** Only species with HK and RR genes are taken into account in the percentages. Alveolates and Monocots do not appear in the table because we have not found RR proteins in the surveyed species belonging to these phyla. Amoeboflagellates do not appear because we have not found HK proteins in the surveyed species classified in this phylum. Phylum abbreviations are given in Table 1.

| Phylum | % of species with 2<odds ratio<10 | % of species with 10<odds ratio<50 | % of species with 50<odds ratio<100 | % of species with odds ratio>100 |
| --- | --- | --- | --- | --- |
| At | 0.00 | 0.00 | 0.00 | 11.96 |
| Aq | 0.00 | 0.00 | 0.00 | 0.00 |
| Ar | 0.00 | 0.00 | 0.00 | 0.00 |
| Ba | 0.00 | 0.48 | 0.00 | 20.77 |
| Cb | 0.00 | 0.00 | 0.00 | 14.29 |
| Cd | 0.00 | 0.00 | 0.00 | 0.00 |
| Cm | 0.00 | 0.00 | 0.00 | 0.00 |
| L | 0.00 | 0.00 | 0.00 | 0.00 |
| V | 0.00 | 0.00 | 0.00 | 20.00 |
| Cf | 0.00 | 0.00 | 8.70 | 34.78 |
| Cr | 0.00 | 0.00 | 0.00 | 100.00 |
| Cy | 0.00 | 0.00 | 0.00 | 10.17 |
| Df | 0.00 | 0.00 | 0.00 | 25.00 |
| Dt | 0.00 | 0.00 | 0.00 | 30.00 |
| Dc | 0.00 | 0.00 | 0.00 | 0.00 |
| El | 0.00 | 0.00 | 0.00 | 0.00 |
| Ac | 0.00 | 0.00 | 0.00 | 66.67 |
| Fb | 0.00 | 0.00 | 0.00 | 0.00 |
| Fi | 0.00 | 0.10 | 0.20 | 7.53 |
| Fu | 0.00 | 0.00 | 0.00 | 0.00 |
| Ge | 0.00 | 0.00 | 0.00 | 100.00 |
| Ni | 0.00 | 0.00 | 0.00 | 0.00 |
| Nt | 0.00 | 0.00 | 0.00 | 50.00 |
| Pl | 0.00 | 0.00 | 0.00 | 25.00 |
| A | 0.00 | 0.00 | 0.24 | 61.22 |
| B | 0.00 | 0.28 | 0.28 | 26.99 |
| D | 0.00 | 2.56 | 7.69 | 62.82 |
| E | 0.00 | 0.24 | 0.00 | 18.09 |
| G | 0.00 | 0.00 | 0.00 | 16.60 |
| Z | 0.00 | 0.00 | 0.00 | 0.00 |
| S | 0.00 | 0.00 | 0.00 | 2.02 |
| Sy | 0.00 | 0.00 | 0.00 | 20.00 |
| T | 0.00 | 0.00 | 0.00 | 0.00 |
| Th | 0.00 | 0.00 | 0.00 | 0.00 |
| Tt | 0.00 | 0.00 | 0.00 | 5.88 |
| C | 0.00 | 0.00 | 0.00 | 0.00 |
| Eu | 0.00 | 0.00 | 0.00 | 6.57 |
| Ta | 0.00 | 0.00 | 0.00 | 0.00 |
| Eg | 0.00 | 0.00 | 0.00 | 0.00 |
| Mi | 0.00 | 0.00 | 0.00 | 0.00 |
| As | 0.00 | 0.00 | 0.00 | 0.00 |
| Bs | 0.00 | 0.00 | 0.00 | 0.00 |
| Ed | 0.00 | 0.00 | 0.00 | 0.00 |
