# Supplementary material for: A survey of HK, HPt, and RR domains and their organization in two-component systems and phosphorelay proteins of organisms with fully sequenced genomes
Source: PeerJ. 2015 Aug 13;3:e1183. doi: 10.7717/peerj.1183 (PMC4558063; doi:10.7717/peerj.1183)
Supplement: Table S6 — Only species with HKRR genes are taken into account in the percentages. Phyla without this type of protein (Aquificae, Tenericutes, Chlamydiae, Crenarchaeota, Korarchaeota, Nanoarchaeota, Nanohaloarchaeota, Alveolates, Euglenozoa, Microsporidians and Monocots) do not appear in the table. Phylum abbreviations are given in Table 1. [file peerj-03-1183-s008.docx]

**Supplementary Table 6. Odds ratios (ratio between the observed and the randomly expected frequency) of HKRR genes located in the genome next to HK_2_ and RR_2_ genes.** Only species with HKRR genes are taken into account in the percentages. Phyla without this type of protein (Aquificae, Chlamydiae , Tenericutes, Crenarchaeota, Alveolates, Euglenozoa, Microsporidians and Monocots) do not appear in the table. Amoeboflagellates do not appear because we have not found HK proteins in the surveyed species classified in this phylum. Phylum abbreviations are given in Table 1.

| Phylum | % of species with 2<odds ratio<10 | % of species with 10<odds ratio<50 | % of species with 50<odds ratio<100 | % of species with odds ratio>100 |
| --- | --- | --- | --- | --- |
| At | 0.00 | 0.00 | 0.72 | 9.42 |
| Ar | 0.00 | 0.00 | 0.00 | 0.00 |
| Ba | 0.00 | 8.18 | 4.40 | 8.18 |
| Cb | 0.00 | 14.29 | 7.14 | 0.00 |
| Cd | 0.00 | 0.00 | 0.00 | 0.00 |
| L | 0.00 | 0.00 | 0.00 | 0.00 |
| V | 14.29 | 14.29 | 0.00 | 0.00 |
| Cf | 0.00 | 25.00 | 5.00 | 10.00 |
| Cr | 0.00 | 100.00 | 0.00 | 0.00 |
| Cy | 1.10 | 34.07 | 8.79 | 4.40 |
| Df | 0.00 | 25.00 | 0.00 | 0.00 |
| Dt | 0.00 | 0.00 | 0.00 | 0.00 |
| Dc | 0.00 | 0.00 | 0.00 | 0.00 |
| El | 0.00 | 0.00 | 0.00 | 100.00 |
| Ac | 0.00 | 33.33 | 0.00 | 44.44 |
| Fb | 0.00 | 0.00 | 0.00 | 0.00 |
| Fi | 0.00 | 1.17 | 2.34 | 4.68 |
| Fu | 0.00 | 0.00 | 0.00 | 0.00 |
| Ge | 0.00 | 0.00 | 0.00 | 0.00 |
| Ni | 0.00 | 0.00 | 100.00 | 0.00 |
| Nt | 0.00 | 50.00 | 0.00 | 0.00 |
| Pl | 0.00 | 5.26 | 26.32 | 26.32 |
| A | 0.00 | 8.94 | 8.94 | 12.57 |
| B | 0.00 | 9.31 | 11.27 | 25.00 |
| D | 16.88 | 42.86 | 2.60 | 2.60 |
| E | 0.00 | 9.09 | 9.09 | 9.09 |
| G | 0.00 | 2.98 | 2.09 | 17.65 |
| Z | 0.00 | 0.00 | 0.00 | 0.00 |
| S | 0.00 | 1.27 | 6.36 | 31.36 |
| Sy | 0.00 | 0.00 | 0.00 | 0.00 |
| Th | 0.00 | 0.00 | 0.00 | 0.00 |
| Tt | 0.00 | 0.00 | 0.00 | 0.00 |
| Eu | 0.00 | 3.77 | 8.49 | 14.15 |
| Ta | 0.00 | 0.00 | 0.00 | 0.00 |
| As | 0.00 | 0.00 | 0.00 | 0.00 |
| Bs | 0.00 | 0.00 | 0.00 | 0.00 |
| Ed | 0.00 | 0.00 | 0.00 | 0.00 |
