# Supplementary material for: A survey of HK, HPt, and RR domains and their organization in two-component systems and phosphorelay proteins of organisms with fully sequenced genomes
Source: PeerJ. 2015 Aug 13;3:e1183. doi: 10.7717/peerj.1183 (PMC4558063; doi:10.7717/peerj.1183)
Supplement: Table S7 — Only species with HKRRHPt proteins are taken into account in the percentages. Prokaryotic phyla without this type of protein (Aquificae, Tenericutes, Deinococcus-Thermus, Fibrobacteres, Elusimicrobia, Armatimonadetes, Nitrospinae, Crenarchaeota, Korarchaeota, Thaumarchaeota, Nanoarchaeota and Nanohaloarchaeota) do not appear in the table. Eukaryotes are not included in the table since we have not found any HKRRHPt protein in this domain. Phylum abbreviations are given in Table 1. [file peerj-03-1183-s009.docx]

**Supplementary Table 7. Odds ratios (ratio between the observed and the randomly expected frequency) of HKRRHPt genes located in the genome next to RR_2_ genes.** Only species with HKRRHPt proteins are taken into account in the percentages. Prokaryotic phyla without this type of protein (Aquificae, Armatimonadetes, Caldiserica, Deinococcus-Thermus, Dictyoglomi, Elusimicrobia, Fibrobacteres, Nitrospinae, Tenericutes, Crenarchaeota and Thaumarchaeota) do not appear in the table. Eukaryotes are not included in the table since we have not found any HKRRHPt protein in this domain. Phylum abbreviations are given in Table 1.

| Phylum | % of species with 2<odds ratio<10 | % of species with 10<odds ratio<50 | % of species with 50<odds ratio<100 | % of species with odds ratio>100 |
| --- | --- | --- | --- | --- |
| At | 0.00 | 85.71 | 14.29 | 0.00 |
| Ba | 1.59 | 33.33 | 25.40 | 4.76 |
| Cb | 0.00 | 0.00 | 0.00 | 0.00 |
| L | 0.00 | 0.00 | 0.00 | 0.00 |
| V | 0.00 | 80.00 | 20.00 | 0.00 |
| Cf | 0.00 | 44.44 | 0.00 | 0.00 |
| Cr | 0.00 | 0.00 | 0.00 | 0.00 |
| Cy | 15.38 | 32.31 | 3.08 | 0.00 |
| Df | 25.00 | 25.00 | 0.00 | 0.00 |
| Ac | 0.00 | 100.00 | 0.00 | 0.00 |
| Fi | 0.00 | 51.02 | 22.45 | 2.04 |
| Fu | 0.00 | 0.00 | 0.00 | 100.00 |
| Ge | 0.00 | 100.00 | 0.00 | 0.00 |
| Nt | 0.00 | 25.00 | 50.00 | 0.00 |
| Pl | 0.00 | 62.50 | 0.00 | 0.00 |
| A | 3.42 | 48.63 | 21.92 | 0.00 |
| B | 1.86 | 60.25 | 26.09 | 0.00 |
| D | 16.92 | 64.62 | 0.00 | 0.00 |
| E | 0.00 | 1.82 | 95.32 | 1.30 |
| G | 5.37 | 68.49 | 1.87 | 0.05 |
| Z | 0.00 | 100.00 | 0.00 | 0.00 |
| S | 14.29 | 35.71 | 14.29 | 0.00 |
| Sy | 0.00 | 100.00 | 0.00 | 0.00 |
| Th | 0.00 | 50.00 | 0.00 | 0.00 |
| Tt | 0.00 | 0.00 | 0.00 | 0.00 |
| Eu | 0.00 | 12.50 | 12.50 | 12.50 |
