# Supplementary material for: A survey of HK, HPt, and RR domains and their organization in two-component systems and phosphorelay proteins of organisms with fully sequenced genomes
Source: PeerJ. 2015 Aug 13;3:e1183. doi: 10.7717/peerj.1183 (PMC4558063; doi:10.7717/peerj.1183)
Supplement: Appendix S1 — File including all figures and tables redone to include hypothetical proteins. Results are similar to those obtained for the dataset where these proteins are excluded. [file peerj-03-1183-s011.zip › plus hypothetical and partial/Supplementary Table 2.docx]

**Supplementary Table 2. Percentage of each TCS/PR protein type per phylum.** Phylum abbreviations are given in Table 1. Only species with proteins containing HK, RR or HPt domains are represented. Korarchaeota, Nanoarchaeota, Nanohaloarchaeota and phyla from the animal kingdom do not appear in the table because we have not found any protein containing HK, RR or HPt domains in the surveyed species classified in these phyla.

| Phylum | RR | HK | HKRR | HPt | HKRRHPt | HK_1_RRHK_2_ | HKRR_1_HPtRR_2_ | HK_1_RR_1_HK_2_RR_2_ |
| --- | --- | --- | --- | --- | --- | --- | --- | --- |
| At | 53.09 | 45.16 | 1.00 | 0.29 | 0.04 | 0.01 | 0.05 | 0.00 |
| Aq | 53.85 | 40.56 | 0.00 | 1.75 | 0.00 | 0.00 | 0.00 | 0.00 |
| Ar | 59.26 | 33.33 | 3.70 | 0.00 | 0.00 | 0.00 | 0.00 | 0.00 |
| Ba | 46.25 | 39.75 | 10.54 | 0.94 | 1.01 | 0.08 | 0.14 | 0.00 |
| Cb | 37.68 | 34.27 | 18.04 | 0.80 | 0.80 | 0.00 | 2.20 | 0.00 |
| Cd | 42.86 | 50.00 | 7.14 | 0.00 | 0.00 | 0.00 | 0.00 | 0.00 |
| Cm | 31.99 | 66.60 | 0.40 | 0.20 | 0.80 | 0.00 | 0.00 | 0.00 |
| L | 39.53 | 44.19 | 9.30 | 4.65 | 2.33 | 0.00 | 0.00 | 0.00 |
| V | 45.98 | 30.00 | 15.00 | 0.98 | 1.18 | 0.20 | 0.39 | 0.20 |
| Cf | 49.25 | 40.06 | 6.92 | 0.31 | 0.71 | 0.00 | 0.04 | 0.00 |
| Cr | 56.12 | 33.67 | 4.08 | 0.00 | 1.02 | 0.00 | 1.02 | 0.00 |
| Cy | 44.27 | 32.87 | 13.40 | 1.04 | 1.77 | 0.26 | 0.86 | 0.19 |
| Df | 46.59 | 37.22 | 8.52 | 0.28 | 3.41 | 0.00 | 0.57 | 0.00 |
| Dt | 54.09 | 41.75 | 1.27 | 0.23 | 0.00 | 0.46 | 0.00 | 0.00 |
| Dc | 50.00 | 46.43 | 3.57 | 0.00 | 0.00 | 0.00 | 0.00 | 0.00 |
| El | 50.00 | 41.67 | 8.33 | 0.00 | 0.00 | 0.00 | 0.00 | 0.00 |
| Ac | 53.12 | 33.37 | 8.72 | 0.86 | 0.10 | 0.48 | 0.38 | 0.29 |
| Fb | 46.15 | 19.23 | 15.38 | 3.85 | 0.00 | 0.00 | 3.85 | 0.00 |
| Fi | 51.36 | 45.95 | 0.62 | 0.58 | 0.09 | 0.13 | 0.04 | 0.00 |
| Fu | 53.17 | 44.05 | 1.44 | 0.77 | 0.19 | 0.00 | 0.00 | 0.00 |
| Ge | 46.46 | 33.86 | 14.17 | 0.79 | 2.36 | 0.00 | 0.00 | 0.00 |
| Ni | 48.15 | 28.40 | 14.81 | 2.47 | 0.00 | 0.00 | 3.70 | 0.00 |
| Nt | 54.88 | 30.98 | 4.71 | 1.35 | 1.35 | 0.00 | 1.35 | 0.00 |
| Pl | 52.37 | 27.04 | 10.82 | 1.43 | 1.93 | 0.00 | 1.11 | 0.00 |
| A | 51. 83 | 33.21 | 7. 74 | 1.29 | 1.15 | 0.04 | 0.26 | 0.07 |
| B | 53.46 | 35. 15 | 6.00 | 0.57 | 1.47 | 0.03 | 0.52 | 0.05 |
| D | 47.23 | 31.62 | 12.22 | 1.34 | 1.60 | 0.26 | 0.68 | 0.11 |
| E | 58.08 | 34.39 | 0.93 | 0.66 | 4.01 | 0.00 | 0.07 | 0.00 |
| G | 51.59 | 35.21 | 4.20 | 0.93 | 4.36 | 0.02 | 0.30 | 0.00 |
| Z | 44.57 | 23.91 | 27.17 | 0.00 | 2.17 | 0.00 | 1.09 | 0.00 |
| S | 47.81 | 34.12 | 9.80 | 1.39 | 0.32 | 0.93 | 0.08 | 0.00 |
| Sy | 52.70 | 37.50 | 2.03 | 1.69 | 2.03 | 0.00 | 0.68 | 0.00 |
| T | 51.69 | 43.00 | 0.00 | 5.31 | 0.00 | 0.00 | 0.00 | 0.00 |
| Th | 49.38 | 32.10 | 11.11 | 1.23 | 2.47 | 0.00 | 0.00 | 0.00 |
| Tt | 52.55 | 40.88 | 0.24 | 0.00 | 1.46 | 0.00 | 0.73 | 0.00 |
| C | 46.67 | 48.89 | 0.00 | 4.44 | 0.00 | 0.00 | 0.00 | 0.00 |
| Eu | 37.23 | 48.46 | 11.44 | 0.13 | 0.15 | 0.02 | 0.05 | 0.00 |
| Ta | 58.71 | 37.42 | 0.32 | 2.26 | 0.00 | 0.00 | 0.00 | 0.00 |
| Av | 0.00 | 58.33 | 0.00 | 8.33 | 0.00 | 0.00 | 0.00 | 0.00 |
| Am | 22.73 | 0.00 | 40.91 | 9.09 | 0.00 | 0.00 | 0.00 | 4.55 |
| Eg | 4.65 | 95.35 | 0.00 | 0.00 | 0.00 | 0.00 | 0.00 | 0.00 |
| Mi | 66.67 | 33.33 | 0.00 | 0.00 | 0.00 | 0.00 | 0.00 | 0.00 |
| As | 15.74 | 31.02 | 24.54 | 11.11 | 0.00 | 6.48 | 0.00 | 2.08 |
| Bs | 14.81 | 18.52 | 37.04 | 7.41 | 0.00 | 7.41 | 0.00 | 14.81 |
| Ed | 54.44 | 15.00 | 13.33 | 15.00 | 0.00 | 0.56 | 0.00 | 0.00 |
| M | 0.00 | 63.64 | 0.00 | 36.36 | 0.00 | 0.00 | 0.00 | 0.00 |
