# Supplementary material for: A survey of HK, HPt, and RR domains and their organization in two-component systems and phosphorelay proteins of organisms with fully sequenced genomes
Source: PeerJ. 2015 Aug 13;3:e1183. doi: 10.7717/peerj.1183 (PMC4558063; doi:10.7717/peerj.1183)
Supplement: Appendix S1 — File including all figures and tables redone to include hypothetical proteins. Results are similar to those obtained for the dataset where these proteins are excluded. [file peerj-03-1183-s011.zip › plus hypothetical and partial/Supplementary Table 3.docx]

**Supplementary Table 3. Percentage of HK genes and RR genes that are neighbors in the genome to other TCS/PR genes.** Phylum abbreviations are given in Table 1. Only species with HK or RR genes are represented.

| Phylum | Orphan HK | HK next to RR | HK next to RR and HK_2_ | HK next to RR, HK_2_ and RR_2_ | Orphan RR | RR next to HK | RR next to HPt | RR next to HKRRHPt | RR next to HKRRHK |
| --- | --- | --- | --- | --- | --- | --- | --- | --- | --- |
| At | 23.53 | 73.55 | 1.72 | 1.07 | 34.68 | 65.16 | 0.15 | 0.05 | 0.01 |
| Aq | 40.52 | 56.90 | 1.72 | 0.00 | 55.84 | 43.51 | 0.65 | 0.00 | 0.00 |
| Ar | 44.44 | 55.56 | 0.00 | 0.00 | 68.75 | 31.25 | 0.00 | 0.00 | 0.00 |
| Ba | 37.89 | 53.74 | 5.59 | 2.55 | 46.93 | 51.00 | 0.61 | 1.43 | 0.08 |
| Cb | 42.11 | 46.78 | 9.36 | 1.75 | 51.06 | 47.87 | 1.06 | 0.00 | 0.00 |
| Cd | 28.57 | 71.43 | 0.00 | 0.00 | 16.67 | 83.33 | 0.00 | 0.00 | 0.00 |
| Cm | 66.77 | 33.23 | 0.00 | 0.00 | 29.56 | 69.18 | 0.00 | 1.26 | 0.00 |
| L | 47.37 | 52.63 | 0.00 | 0.00 | 35.29 | 64.71 | 0.00 | 0.00 | 0.00 |
| V | 30.07 | 64.38 | 4.25 | 1.31 | 53.30 | 44.35 | 0.43 | 1.92 | 0.21 |
| Cf | 37.87 | 54.37 | 4.76 | 2.99 | 49.10 | 50.09 | 0.18 | 0.72 | 0.00 |
| Cr | 51.52 | 15.15 | 12.12 | 21.21 | 80.00 | 20.00 | 0.00 | 0.00 | 0.00 |
| Cy | 64.40 | 32.14 | 2.65 | 0.70 | 73.41 | 25.43 | 0.19 | 0.85 | 0.19 |
| Df | 41.22 | 51.91 | 4.58 | 2.29 | 48.17 | 45.12 | 0.61 | 6.10 | 0.00 |
| Dt | 31.77 | 62.15 | 3.04 | 3.04 | 47.33 | 52.45 | 0.00 | 0.00 | 0.21 |
| Dc | 38.46 | 61.54 | 0.00 | 0.00 | 42.86 | 57.14 | 0.00 | 0.00 | 0.00 |
| El | 60.00 | 40.00 | 0.00 | 0.00 | 83.33 | 16.67 | 0.00 | 0.00 | 0.00 |
| Ac | 29.89 | 60.34 | 4.60 | 5.17 | 54.87 | 44.04 | 0.54 | 0.18 | 0.36 |
| Fb | 20.00 | 80.00 | 0.00 | 0.00 | 58.33 | 33.33 | 8.33 | 0.00 | 0.00 |
| Fi | 13.73 | 82.40 | 2.34 | 1.43 | 22.77 | 76.78 | 0.17 | 0.11 | 0.17 |
| Fu | 18.95 | 78.87 | 2.18 | 0.00 | 33.39 | 65.70 | 0.54 | 0.36 | 0.00 |
| Ge | 27.91 | 62.79 | 4.65 | 4.65 | 44.07 | 50.85 | 0.00 | 5.08 | 0.00 |
| Ni | 30.43 | 69.57 | 0.00 | 0.00 | 56.41 | 43.59 | 0.00 | 0.00 | 0.00 |
| Nt | 14.13 | 70.65 | 8.70 | 5.43 | 48.47 | 47.85 | 1.84 | 1.84 | 0.00 |
| Pl | 40.89 | 53.66 | 2.56 | 2.39 | 67.11 | 29.99 | 1.32 | 1.67 | 0.00 |
| A | 35. 37 | 54. 71 | 3.68 | 6.01 | 56.76 | 40. 66 | 1.00 | 1.65 | 0.06 |
| B | 14.42 | 80.85 | 1.71 | 2.65 | 41.09 | 56.21 | 0.57 | 2.20 | 0.03 |
| D | 43.34 | 44.91 | 5.20 | 5.60 | 60.35 | 36.79 | 0.99 | 2.18 | 0.03 |
| E | 24.70 | 68.82 | 3.37 | 2.96 | 48.97 | 44.26 | 0.07 | 6.74 | 0.00 |
| G | 12.32 | 81.97 | 3.09 | 1.81 | 35.25 | 60.00 | 0.91 | 4.00 | 0.02 |
| Z | 18.18 | 81.82 | 0.00 | 0.00 | 53.66 | 43.90 | 0.00 | 2.44 | 0.00 |
| S | 50.39 | 47.09 | 0.78 | 0.25 | 65.64 | 33.90 | 0.23 | 0.21 | 0.04 |
| Sy | 24.32 | 72.07 | 0.00 | 3.60 | 41.67 | 53.85 | 0.64 | 3.85 | 0.00 |
| T | 12.36 | 87.64 | 0.00 | 0.00 | 27.10 | 72.90 | 0.00 | 0.00 | 0.00 |
| Th | 34.62 | 65.38 | 0.00 | 0.00 | 52.50 | 45.00 | 0.00 | 2.50 | 0.00 |
| Tt | 42.26 | 56.55 | 0.00 | 1.19 | 52.78 | 44.91 | 0.00 | 2.31 | 0.00 |
| C | 77.27 | 22.73 | 0.00 | 0.00 | 76.19 | 23.81 | 0.00 | 0.00 | 0.00 |
| Eu | 76.49 | 19.95 | 2.72 | 0.71 | 71.33 | 28.41 | 0.13 | 0.13 | 0.00 |
| Ta | 43.10 | 42.24 | 6.90 | 1.72 | 66.48 | 30.22 | 3.30 | 0.00 | 0.00 |
| Av | 100.00 | 0.00 | 0.00 | 0.00 | - | - | - | - | 0.00 |
| Am | - | - | - | - | 100.00 | 0.00 | 0.00 | 0.00 | 0.00 |
| Eg | 100.00 | 0.00 | 0.00 | 0.00 | 100.00 | 0.00 | 0.00 | 0.00 | 0.00 |
| Mi | 100.00 | 0.00 | 0.00 | 0.00 | 100.00 | 0.00 | 0.00 | 0.00 | 0.00 |
| As | 100.00 | 0.00 | 0.00 | 0.00 | 100.00 | 0.00 | 0.00 | 0.00 | 0.00 |
| Bs | 100.00 | 0.00 | 0.00 | 0.00 | 100.00 | 0.00 | 0.00 | 0.00 | 0.00 |
| Ed | 100.00 | 0.00 | 0.00 | 0.00 | 100.00 | 0.00 | 0.00 | 0.00 | 0.00 |
| M | 100.00 | 0.00 | 0.00 | 0.00 | - | - | - | - | - |
