# Supplementary material for: A survey of HK, HPt, and RR domains and their organization in two-component systems and phosphorelay proteins of organisms with fully sequenced genomes
Source: PeerJ. 2015 Aug 13;3:e1183. doi: 10.7717/peerj.1183 (PMC4558063; doi:10.7717/peerj.1183)
Supplement: Appendix S1 — File including all figures and tables redone to include hypothetical proteins. Results are similar to those obtained for the dataset where these proteins are excluded. [file peerj-03-1183-s011.zip › plus hypothetical and partial/Supplementary Table 4.docx]

**Supplementary Table 4. Odds ratios (ratio between the observed and the randomly expected frequency) of HK genes located next to RR and**

**HK_2_ genes in the genome.** Only species with HK and RR genes are taken into account in the percentages. Alveolates and Monocots do not appear in the table because we have not found RR proteins in the surveyed species belonging to these phyla. Amoeboflagellates do not appear because we have not found HK proteins in the surveyed species classified in this phylum. Phylum abbreviations are given in Table 1.

| Phylum | % of species with 2<odds ratio<10 | % of species with 10<odds ratio<50 | % of species with 50<odds ratio<100 | % of species with odds ratio>100 |
| --- | --- | --- | --- | --- |
| At | 1.26 | 7.56 | 2.52 | 7.09 |
| Aq | 0.00 | 0.00 | 7.69 | 0.00 |
| Ar | 0.00 | 0.00 | 0.00 | 0.00 |
| Ba | 2.33 | 18.60 | 10.23 | 6.05 |
| Cb | 0.00 | 7.14 | 0.00 | 21.43 |
| Cd | 0.00 | 0.00 | 0.00 | 0.00 |
| Cm | 0.00 | 0.00 | 0.00 | 0.00 |
| L | 0.00 | 0.00 | 0.00 | 0.00 |
| V | 0.00 | 20.00 | 10.00 | 20.00 |
| Cf | 13.04 | 39.13 | 0.00 | 0.00 |
| Cr | 0.00 | 100.00 | 0.00 | 0.00 |
| Cy | 6.78 | 16.10 | 1.69 | 3.39 |
| Df | 0.00 | 25.00 | 0.00 | 0.00 |
| Dt | 0.00 | 15.00 | 0.00 | 0.00 |
| Dc | 0.00 | 0.00 | 0.00 | 0.00 |
| El | 0.00 | 0.00 | 0.00 | 0.00 |
| Ac | 0.00 | 55.56 | 0.00 | 0.00 |
| Fb | 0.00 | 0.00 | 0.00 | 0.00 |
| Fi | 2.33 | 10.57 | 3.59 | 3.44 |
| Fu | 0.00 | 0.00 | 0.00 | 13.16 |
| Ge | 0.00 | 100.00 | 0.00 | 0.00 |
| Ni | 0.00 | 0.00 | 0.00 | 0.00 |
| Nt | 0.00 | 50.00 | 0.00 | 0.00 |
| Pl | 0.00 | 20.00 | 20.00 | 0.00 |
| A | 1.14 | 18.22 | 9.57 | 10.93 |
| B | 4.18 | 11.98 | 1.39 | 1.67 |
| D | 27.16 | 30.86 | 0.00 | 1.23 |
| E | 1.46 | 1.22 | 0.24 | 16.83 |
| G | 1.18 | 16.64 | 19.55 | 8.37 |
| Z | 0.00 | 0.00 | 0.00 | 0.00 |
| S | 0.38 | 1.91 | 3.05 | 3.05 |
| Sy | 0.00 | 0.00 | 0.00 | 0.00 |
| T | 0.00 | 0.00 | 0.00 | 0.00 |
| Th | 0.00 | 0.00 | 0.00 | 0.00 |
| Tt | 0.00 | 0.00 | 0.00 | 0.00 |
| C | 0.00 | 0.00 | 0.00 | 0.00 |
| Eu | 0.73 | 8.03 | 8.03 | 8.76 |
| Ta | 0.00 | 25.00 | 12.50 | 12.50 |
| Eg | 0.00 | 0.00 | 0.00 | 0.00 |
| Mi | 0.00 | 0.00 | 0.00 | 0.00 |
| As | 0.00 | 0.00 | 0.00 | 0.00 |
| Bs | 0.00 | 0.00 | 0.00 | 0.00 |
| Ed | 0.00 | 0.00 | 0.00 | 0.00 |
