# Supplementary material for: A survey of HK, HPt, and RR domains and their organization in two-component systems and phosphorelay proteins of organisms with fully sequenced genomes
Source: PeerJ. 2015 Aug 13;3:e1183. doi: 10.7717/peerj.1183 (PMC4558063; doi:10.7717/peerj.1183)
Supplement: Appendix S1 — File including all figures and tables redone to include hypothetical proteins. Results are similar to those obtained for the dataset where these proteins are excluded. [file peerj-03-1183-s011.zip › plus hypothetical and partial/Supplementary Table 8.docx]

**Supplementary Table 8. Odds ratios (ratio between the observed and the randomly expected frequency) of HKRRHK_2_ genes located in the genome next to RR_2_ genes.** Only species with HKRRHK_2_ proteins are taken into account in the percentages. Phyla without this type of protein (Aquificae, Armatimonadetes, Chlorobi, Caldiserica, Chlamydiae, Lentisphaerae, Chloroflexi, Chrysiogenetes, Deferribacteres, Dictyoglomi, Elusimicrobia, Fibrobacteres, Fusobacteria, Gemmatimonadetes, Nitrospinae, Nitrospirae, Planctomycetes, Epsilonproteobacteria, Zetaproteobacteria, Synergistetes, Tenericutes, Thermodesulfobacteria, Thermotogae, Crenarchaeota, Thaumarchaeota, Alveolates, Amoeboflagellate, Euglenozoa, Microsporidians and Monocots) do not appear in the table. Eukaryotic phyla with HKRRHK_2_ genes are not included in this statistics because none of those HKRRHK_2_ genes have been found neighboring an RR gene. Phylum abbreviations are given in Table 1.

| Phylum | % of species with 2<odds ratio<10 | % of species with 10<odds ratio<50 | % of species with 50<odds ratio<100 | % of species with odds ratio>100 |
| --- | --- | --- | --- | --- |
| At | 0.00 | 0.00 | 0.00 | 25.00 |
| Ba | 0.00 | 22.22 | 0.00 | 11.11 |
| V | 0.00 | 50.00 | 0.00 | 0.00 |
| Cy | 0.00 | 38.89 | 0.00 | 0.00 |
| Dt | 0.00 | 33.33 | 0.00 | 0.00 |
| Ac | 0.00 | 33.33 | 0.00 | 0.00 |
| Fi | 1.43 | 44.29 | 40.00 | 0.00 |
| A | 0.00 | 60.00 | 10.00 | 0.00 |
| B | 0.00 | 22.22 | 22.22 | 0.00 |
| D | 7.69 | 7.69 | 0.00 | 0.00 |
| G | 0.00 | 20.00 | 3.33 | 30.00 |
| S | 0.00 | 3.37 | 0.00 | 0.00 |
| Eu | 0.00 | 0.00 | 0.00 | 0.00 |
