# Supplementary material for: A survey of HK, HPt, and RR domains and their organization in two-component systems and phosphorelay proteins of organisms with fully sequenced genomes
Source: PeerJ. 2015 Aug 13;3:e1183. doi: 10.7717/peerj.1183 (PMC4558063; doi:10.7717/peerj.1183)
Supplement: Appendix S1 — File including all figures and tables redone to include hypothetical proteins. Results are similar to those obtained for the dataset where these proteins are excluded. [file peerj-03-1183-s011.zip › plus hypothetical and partial/Table 1.docx]

**Table 1. Percentage of species in each phylum with TCS/PR proteins.**

| **Domain** | **Phylum** | **Abbreviaton** | **nº of species surveyed** | **% of species with**  **HK and RR domains** | **% of species with HPt domains** |
| --- | --- | --- | --- | --- | --- |
| Bacteria | Actinobacteria | At | 635 | 100.00 | 14.80 |
| Bacteria | Aquificae | Aq | 13 | 100.00 | 76.92 |
| Bacteria | Armatimonadetes | Ar | 1 | 100.00 | 100.00 |
| Bacteria | Bacteroidetes | Ba | 235 | 92.77 | 53.62 |
| Bacteria | Chlorobi | Cb | 14 | 100.00 | 71.43 |
| Bacteria | Caldiserica | Cd | 1 | 100.00 | 0.00 |
| Bacteria | Chlamydiae | Cm | 108 | 98.15 | 2.78 |
| Bacteria | Lentisphaerae | L | 1 | 100.00 | 100.00 |
| Bacteria | Verrucomicrobia | V | 10 | 100.00 | 80.00 |
| Bacteria | Chloroflexi | Cf | 23 | 100.00 | 65.22 |
| Bacteria | Chrysiogenetes | Cr | 1 | 100.00 | 100.00 |
| Bacteria | Cyanobacteria | Cy | 118 | 100.00 | 75.42 |
| Bacteria | Deferribacteres | Df | 4 | 100.00 | 0.00 |
| Bacteria | Deinococcus-Thermus | Dt | 20 | 100.00 | 35.00 |
| Bacteria | Dictyoglomi | Dc | 2 | 100.00 | 0.00 |
| Bacteria | Elusimicrobia | El | 1 | 100.00 | 0.00 |
| Bacteria | Acidobacteria | Ac | 9 | 100.00 | 100.00 |
| Bacteria | Fibrobacteres | Fb | 1 | 100.00 | 100.00 |
| Bacteria | Firmicutes | Fi | 2066 | 100.00 | 39.06 |
| Bacteria | Fusobacteria | Fu | 38 | 100.00 | 28.95 |
| Bacteria | Gemmatimonadetes | Ge | 1 | 100.00 | 100.00 |
| Bacteria | Nitrospinae | Ni | 1 | 100.00 | 100.00 |
| Bacteria | Nitrospirae | Nt | 4 | 100.00 | 100.00 |
| Bacteria | Planctomycetes | Pl | 20 | 100.00 | 100.00 |
| Bacteria | Alphaproteobacteria | A | 451 | 99.78 | 58.54 |
| Bacteria | Betaproteobacteria | B | 366 | 98.63 | 60.38 |
| Bacteria | Deltaproteobacteria | D | 82 | 100.00 | 98.78 |
| Bacteria | Epsilonproteobacteria | E | 410 | 100.00 | 99.27 |
| Bacteria | Gammaproteobacteria | G | 2246 | 98.84 | 95.68 |
| Bacteria | Zetaproteobacteria | Z | 1 | 100.00 | 100.00 |
| Bacteria | Spirochaetes | S | 274 | 100.00 | 99.64 |
| Bacteria | Synergistetes | Sy | 11 | 100.00 | 63.64 |
| Bacteria | Tenericutes | T | 111 | 16.22 | 7.21 |
| Bacteria | Thermodesulfobacteria | Th | 2 | 100.00 | 100.00 |
| Bacteria | Thermotogae | Tt | 17 | 100.00 | 100.00 |
| Archaea | Crenarchaeota | C | 51 | 9.80 | 3.92 |
| Archaea | Euryarchaeota | Eu | 179 | 87.71 | 66.48 |
| Archaea | Korarchaeota | K | 1 | 0.00 | 0.00 |
| Archaea | Thaumarchaeota | Ta | 11 | 90.91 | 63.64 |
| Archaea | Nanoarchaeota | N | 1 | 0.00 | 0.00 |
| Archaea | Nanohaloarchaeota | Nh | 1 | 0.00 | 0.00 |
| Eukarya | Alveolates | Av | 5 | 0.00 | 20.00 |
| Eukarya | Amoeboflagellates | Am | 1 | 100.00 | 100.00 |
| Eukarya | Euglenozoa | Eg | 5 | 40.00 | 0.00 |
| Eukarya | Microsporidians | Mi | 2 | 50.00 | 0.00 |
| Eukarya | Ascomycetes | As | 31 | 80.65 | 96.77 |
| Eukarya | Basidiomycetes | Bs | 2 | 100.00 | 100.00 |
| Eukarya | Eudicots | Ed | 2 | 100.00 | 100.00 |
| Eukarya | Monocots | M | 1 | 0.00 | 100.00 |
| Eukarya | Nematodes | - | 1 | 0.00 | 0.00 |
| Eukarya | Arthropods | - | 7 | 57.14 | 42.86 |
| Eukarya | Chordates | - | 10 | 90.00 | 90.00 |
