# Supplementary material for: A survey of HK, HPt, and RR domains and their organization in two-component systems and phosphorelay proteins of organisms with fully sequenced genomes
Source: PeerJ. 2015 Aug 13;3:e1183. doi: 10.7717/peerj.1183 (PMC4558063; doi:10.7717/peerj.1183)
Supplement: Appendix S1 — File including all figures and tables redone to include hypothetical proteins. Results are similar to those obtained for the dataset where these proteins are excluded. [file peerj-03-1183-s011.zip › plus hypothetical and partial/Table 2.docx]

**Table 2. Types of TCS/PR proteins found in the 7609 surveyed species**. The protein identifier describes the type (HK, HPt, or RR) and number of TCS/PR domains fused in each protein.

| **Protein type** | **Total number of proteins found** | **Percentage of proteomes with this type of protein** | **Number of species with this type of protein** | **Average number of proteins/organism** |
| --- | --- | --- | --- | --- |
| RR | 220873 | 97,21 | 7397 | 29,86 |
| HK | 165470 | 97,33 | 7406 | 22,34 |
| HKRR | 19905 | 50,64 | 3853 | 5,17 |
| HKRRHPt | 9381 | 41,52 | 3159 | 2,97 |
| HKHPt | 5646 | 43,13 | 3282 | 1,72 |
| HPt | 3682 | 28,68 | 2182 | 1,69 |
| RR_1_RR_2_ | 2049 | 17,72 | 1348 | 1,52 |
| HKRR_1_RR_2_ | 2228 | 14,57 | 1109 | 2,01 |
| HKRR_1_HPtRR_2_ | 1072 | 8,86 | 674 | 1,59 |
| HK_1_RR_1_RR_2_RR_3_ | 607 | 6,83 | 520 | 1,17 |
| HK_1_HK_2_ | 552 | 5,01 | 381 | 1,45 |
| HK_1_RRHK_2_ | 438 | 3,55 | 270 | 1,62 |
| RRHPt | 340 | 3,77 | 287 | 1,18 |
| HKRRHPt_1_HPt_2_HPt_3_ | 154 | 2,02 | 154 | 1,00 |
| RR_1_RR_2_HPt | 131 | 1,46 | 111 | 1,18 |
| HKRRHPt_1_HPt_2_HPt_3_HPt_4_ | 109 | 1,43 | 109 | 1,00 |
| HK_1_RR_1_HK_2_RR_2_ | 98 | 0,85 | 65 | 1,51 |
| RR_1_RR_2_RR_3_HPt | 72 | 0,51 | 39 | 1,85 |
| HKRRHPt_1_HPt_2_HPt_3_HPt_4_HPt_5_ | 67 | 0,88 | 67 | 1,00 |
| HKRRHPt_1_HPt_2_ | 62 | 0,78 | 59 | 1,05 |
| HK_1_HK_2_RRHPt | 42 | 0,54 | 41 | 1,02 |
| HK_1_HK_2_HPt | 40 | 0,51 | 39 | 1,03 |
| HKHPt_1_HPt_2_ | 38 | 0,49 | 37 | 1,03 |
| RR_1_RR_2_RR_3_ | 38 | 0,37 | 28 | 1,36 |
| HKRR_1_RR_2_RR_3_HPt | 34 | 0,38 | 29 | 1,17 |
| HPt_1_HPt_2_ | 26 | 0,24 | 18 | 1,44 |
| HKHPt_1_HPt_2_HPt_3_ | 16 | 0,20 | 15 | 1,07 |
| HK_1_HK_2_RR_1_RR_2_RR_3_ | 9 | 0,12 | 9 | 1,00 |
| HK_1_HK_2_HK_3_ | 9 | 0,04 | 3 | 3,00 |
| HKRR_1_RR_2_RR_3_RR_4_RR_5_HPt | 7 | 0,09 | 7 | 1,00 |
| HKRRHPt_1_HPt_2_HPt_3_HPt_4_HPt_5_HPt_6_HPt_7_ | 7 | 0,09 | 7 | 1,00 |
| HKRR_1_RR_2_RR_3_RR_4_ | 6 | 0,08 | 6 | 1,00 |
| HK_1_HK_2_HK_3_HK_4_RR_1_RR_2_ | 6 | 0,08 | 6 | 1,00 |
| HK_1_HK_2_RRHPt_1_HPt_2_ | 5 | 0,07 | 5 | 1,00 |
| HKRR_1_RR_2_RR_3_RR_4_HPt | 5 | 0,07 | 5 | 1,00 |
| RR_1_RR_2_RR_3_RR_4_ | 2 | 0,03 | 2 | 1,00 |
| HK_1_HK_2_RR_1_RR_2_HPt_1_HPt_2_ | 2 | 0,03 | 2 | 1,00 |
| HK_1_HK_2_RR_1_RR_2_RR_3_RR_4_ | 2 | 0,03 | 2 | 1,00 |
| HK_1_HK_2_HK_3_HK_4_ | 2 | 0,03 | 2 | 1,00 |
| HK_1_HK_2_HPt_1_HPt_2_ | 2 | 0,03 | 2 | 1,00 |
| HKRR_1_RR_2_HPt_1_HPt_2_ | 2 | 0,03 | 2 | 1,00 |
| HK_1_HK_2_HK_3_RR | 1 | 0,01 | 1 | 1,00 |
| HPt_1_HPt_2_HPt_3_ | 2 | 0,03 | 2 | 1,00 |
| HK_1_HK_2_RRHPt_1_HPt_2_HPt_3_ | 1 | 0,01 | 1 | 1,00 |
| HKRR_1_RR_2_RR_3_HPt_1_HPt_2_HPt_3_ | 1 | 0,01 | 1 | 1,00 |
| HPt_1_HPt_2_HPt_3_HPt_4_ | 3 | 0,04 | 3 | 1,00 |
| HKRR_1_RR_2_HPt_1_HPt_2_HPt_3_ | 1 | 0,01 | 1 | 1,00 |
| HK_1_HK_2_RR_1_RR_2_HPt | 1 | 0,01 | 1 | 1,00 |
| HK_1_HK_2_RR_1_RR_2_RR_3_RR_4_RR_5_RR_6_HPt | 1 | 0,01 | 1 | 1,00 |
| HKRRHPt_1_HPt_2_HPt_3_HPt_4_HPt_5_HPt_6_ | 1 | 0,01 | 1 | 1,00 |
| HK_1_HK_2_RR_1_RR_2_RR_3_RR_4_RR_5_RR_6_ | 2 | 0.03 | 2 | 1,00 |
| HPt_1_HPt_2_HPt_3_HPt_4_HPt_5_ | 2 | 0.03 | 2 | 1,00 |
