# Supplementary material for: A survey of HK, HPt, and RR domains and their organization in two-component systems and phosphorelay proteins of organisms with fully sequenced genomes
Source: PeerJ. 2015 Aug 13;3:e1183. doi: 10.7717/peerj.1183 (PMC4558063; doi:10.7717/peerj.1183)
Supplement: Appendix S1 — File including all figures and tables redone to include hypothetical proteins. Results are similar to those obtained for the dataset where these proteins are excluded. [file peerj-03-1183-s011.zip › plus hypothetical and partial/Table 3.docx]

**Table 3. Total number of HKRRHPt and HKRRHK proteins found in prokaryotic phyla.** Phyla in bold are from the bacterial domain. Italicized phyla are from the archaeal domain.

| **Phylum** | **Number of HKRRHPt/HK_1_RRHK_2_ proteins found** | **Number of HKRRHPt/HK_1_RRHK_2_ genes with a neighboring RR gene** | **% of HKRRHPt / HK_1_RRHK_2_ genes with a neighboring RR gene** |
| --- | --- | --- | --- |
| Actinobacteria | 12/4 | 9/1 | 75.00/25.00 |
| Aquificae | 0/0 | 0/0 | -/- |
| Armatimonadetes | 0/0 | 0/0 | -/- |
| Bacteroidetes | 133/10 | 86/5 | 64.66/50.00 |
| Chlorobi | 4/0 | 0/0 | 0.00/- |
| Caldiserica | 0/0 | 0/0 | -/- |
| Chlamydiae | 4/0 | 2/0 | 50.00/- |
| Lentisphaerae | 1/0 | 0/0 | 0.00/- |
| Verrucomicrobia | 12/2 | 9/1 | 75.00/50.00 |
| Chloroflexi | 16/0 | 8/0 | 50.00/- |
| Chrysiogenetes | 1/0 | 0/0 | 0.00/- |
| Cyanobacteria | 193/28 | 40/9 | 20.73/32.14 |
| Deferribacteres | 12/0 | 10/0 | 83.33/- |
| Deinococcus-Thermus | 0/4 | 0/1 | -/25.00 |
| Dictyoglomi | 0/0 | 0/0 | -/- |
| Elusimicrobia | 0/0 | 0/0 | -/- |
| Acidobacteria | 1/5 | 1/2 | 100.00/40.00 |
| Fibrobacteres | 0/0 | 0/0 | -/- |
| Firmicutes | 76/114 | 51/79 | 67.11/69.30 |
| Fusobacteria | 2/0 | 2/0 | 100.00/- |
| Gemmatimonadetes | 3/0 | 3/0 | 100.00/- |
| Nitrospinae | 0/0 | 0/0 | -/- |
| Nitrospirae | 4/0 | 3/0 | 75.00/- |
| Planctomycetes | 42/0 | 18/0 | 42.86/- |
| Alphaproteobacteria | 347/11 | 237/7 | 68.30/63.64 |
| Betaproteobacteria | 384/9 | 292/4 | 76.04/44.44 |
| Deltaproteobacteria | 213/35 | 135/2 | 63.38/5.71 |
| Epsilonproteobacteria | 402/0 | 392/0 | 97.51/- |
| Gammaproteobacteria | 7440/30 | 3443/16 | 46.28/53.33 |
| Zetaproteobacteria | 2/0 | 1/0 | 50.00/- |
| Spirochaetes | 54/154 | 17/3 | 31.48/1.95 |
| Synergistetes | 6/0 | 6/0 | 100.00/- |
| Tenericutes | 0/0 | 0/0 | -/- |
| Thermodesulfobacteria | 2/0 | 1/0 | 50.00/- |
| Thermotogae | 6/0 | 5/0 | 83.33/- |
| *Crenarchaeota* | 0/0 | 0/0 | -/- |
| *Euryarchaeota* | 9/1 | 3/0 | 33.33/0.00 |
| *Thaumarchaeota* | 0/0 | 0/0 | -/- |
| **Total** | **9381/407** | **4774/130** | **50.89/31.94** |
